# Cell deformations generated by stochastic actomyosin waves drive *in vivo* random-walk swimming migration

**DOI:** 10.1242/jcs.263787

**Published:** 2025-05-21

**Authors:** Cyril Andrieu, Bren Hunyi Lee, Anna Franz

**Affiliations:** Department of Cell and Developmental Biology, University College London, London, WC1E 6BT, UK

**Keywords:** Swimming cell migration, Adipocytes, Actin, *Drosophila*

## Abstract

Amoeboid cell migration drives many developmental and disease-related processes, including immune responses and cancer metastasis. Swimming migration is a subtype of amoeboid migration that is observed in cells in suspension *ex vivo.* However, the mechanism underlying swimming migration *in vivo* is unknown. Using *Drosophila* fat body cells (FBCs) as a model, we show that FBCs actively swim to patrol the pupa by random walk. Their migration is powered through actomyosin waves that exert compressive forces as they travel to the cell rear, causing cell deformations. Unlike in other types of amoeboid migration, Rho1 (the *Drosophila* orthologue of RhoA), Cdc42 and Rac1 are all required for regulation of formin-driven actin polymerization during FBC migration. We find that Rho1 at the cell rear induces actomyosin contractions via Rho kinase and myosin II. We show that contractile actin waves display a stochastic behaviour, inducing either cell elongation or rounding, suggesting that non-reciprocal cell deformations drive locomotion. Importantly, our work in a physiological system reveals that stochastic actomyosin waves promote random-walk swimming migration to enable fast, long-range cell dispersal. We propose that this individualist migration behaviour collectively allows patrolling of the pupal body.

## Teaser

**Highlighted Article:** Using *Drosophila* adipocytes as an *in vivo* model of swimming migration, a subtype of amoeboid migration, we show that cell deformations generated by stochastic actomyosin waves drive random-walk swimming migration.

## INTRODUCTION

Cell motility is key to many biological processes such as embryonic development, immune responses and cancer invasion. Cells can use two main modes of cell migration: mesenchymal migration or amoeboid migration (Paluch et al., 2016). Mesenchymal migration has been studied extensively, resulting in a detailed understanding of the mechanisms regulating force generation and force transmission. Mesenchymal migration is generally characterized by a slow migration speed and elongated cell shape. A large flat lamellipodium containing branched actin extends at the cell front. The cell is anchored to the substrate via integrin-dependent focal adhesions. The cell rear then retracts through actomyosin contractions that are accompanied by the dissolution of focal adhesions, thereby propelling the cell body forward (Lauffenburger and Horwitz, 1996; Ridley et al., 2003).

Whereas cells *ex vivo* on flat adhesive substrates usually migrate by mesenchymal migration, cells in more complex three-dimensional (3D) environments *ex vivo* and *in vivo* often use alternative migration modes. These have in common that they do not involve lamellipodia, and they are collectively referred to as amoeboid migration (Paluch et al., 2016). A range of cell types use amoeboid migration, including embryonic cells (Ruprecht et al., 2015; Lin et al., 2022; Kardash et al., 2010), immune cells (Aoun et al., 2020; Garcia-Seyda et al., 2021; Lammermann et al., 2008; Liu et al., 2015; O'Neill et al., 2018) and some types of cancer cells (Bergert et al., 2015; Poincloux et al., 2011; Ullo and Logue, 2021). Despite the wide range of amoeboid migration mechanisms employed by these different cell types in various environments, the common features are a rounded cell shape, fast migration speed, low or no cell–substrate adhesion, and the dependence on myosin-dependent cortical actin flows (Paluch et al., 2016). Since amoeboid migration does not feature lamellipodia or filopodia, it is thought to be mostly independent of Rac1, Cdc42 or Arp2/3-driven branched actin formation (Garcia-Arcos et al., 2024a), which mediate the generation of these front protrusions in mesenchymal migration (Ridley, 2001; 2015). Indeed, amoeboid migration of *Drosophila* germ cells (Kunwar et al., 2003; Lin et al., 2020) and *in vitro* swimming migration of macrophages upon optogenetic activation of RhoA (O'Neill et al., 2018) do not rely on Rac1 function. However, amoeboid blebbing migration relies on actin polymerization controlled by Rac1 (Kardash et al., 2010).

Although lacking lamellipodia, cells using amoeboid migration can use other types of protrusions, including blebs (Bergert et al., 2015; Blaser et al., 2006) or giant blebs (Ruprecht et al., 2015; Liu et al., 2015; Bergert et al., 2015; Ullo and Logue, 2021; Logue et al., 2015), or can completely lack protrusions (O'Neill et al., 2018; Poincloux et al., 2011). Together, these characteristics give plasticity to amoeboid migratory cells to adapt and move in different 3D environments, in confined conditions and even in solution.

Sperm, bacteria and many protists can migrate by swimming through liquids using cilia or flagella (Elgeti et al., 2015; Ginger et al., 2008). Moreover, it has recently been reported that other cells that lack cilia or flagella can also swim *in vitro* when placed in solution, including the amoeba *Dictyostelium* (Barry and Bretscher, 2010; Howe et al., 2013; Van Haastert, 2011) and mammalian immune cells such as neutrophils (Garcia-Seyda et al., 2021; Barry and Bretscher, 2010), primary human T lymphocytes (Aoun et al., 2020) as well as RAW 264.7 macrophages upon optogenetic RhoA activation (O'Neill et al., 2018). In the case of macrophages and T lymphocytes, force generation during swimming migration is powered by a continuous rearward flow of cortical actin (O'Neill et al., 2018; Aoun et al., 2020). In both cell types, force transmission during swimming is mediated by coupling the cortical actin flow to the rearward treadmilling of transmembrane proteins at the cell surface to exert viscous forces on the surrounding liquid (O'Neill et al., 2018; Aoun et al., 2020). In addition to membrane treadmilling, a computational model has proposed an alternative mechanism for force transmission in amoeboid blebbing migration in which non-reciprocal cell shape changes can theoretically drive force transmission (Lim et al., 2013). However, no evidence supporting this alternative mechanism was found for the *ex vivo* swimming of macrophages and T lymphocytes (O'Neill et al., 2018; Aoun et al., 2020). Overall, these studies have provided important first insights into the mechanisms driving swimming migration *ex vivo*, especially with regards to force transmission. However, it is still unknown whether these cells, or any other cell types, can migrate by swimming in the highly complex 3D environment found *in vivo* and, if so, what mechanism of force generation and force transmission cells might use to swim *in vivo*.

In previous work (Franz et al., 2018), we discovered that *Drosophila* fat body cell (FBC) migration exhibits key hallmarks of swimming migration, demonstrating that this mode of migration is used by cells *in vivo* and making FBCs the first physiological model system in which this migration mode can be studied. We showed that FBCs, the *Drosophila* equivalent of vertebrate adipocytes, use directed swimming cell migration to actively move across the hemolymph, the body fluid of the fly, to reach skin wounds (Franz et al., 2018). We found that in pupae, these giant spherical, polyploid cells use actin waves to actively swim with high directional persistence to reach wounds, where they play several local roles in wound healing (Franz et al., 2018). However, apart from the involvement of actin waves, the mechanism underlying force generation and force transmission during *in vivo* swimming migration under physiological conditions remains unknown.

Here, we establish a new high-throughput model of *in vivo* non-directed swimming migration to study the mechanism underlying this unusual migration mode. We show that in absence of a wound, instead of using directed cell migration, FBCs use non-directed, random-walk swimming migration to patrol the whole pupa. By combining our newly developed high-throughput genetic screening protocol with high- and super-resolution *in vivo* live imaging, we show that swimming migration is powered by dynamic cortical actomyosin waves that contract at the cell rear. Actin wave generation is regulated by the small Rho GTPases Rho1 (the *Drosophila* RhoA orthologue), Rac1 and Cdc42, as well as their key downstream effectors Dia, Arp2/3, Rho kinase and myosin II, to induce actin polymerization and actomyosin contraction. Moreover, our analysis links swimming migration to dynamic cell deformations, including active cell elongation as well as active rounding. This supports a model of non-reciprocal cell shape changes driving force transmission during *in vivo* swimming migration.

## RESULTS

### FBCs use active non-directed, random-walk migration to patrol the pupa during development

**Fig. 1.**
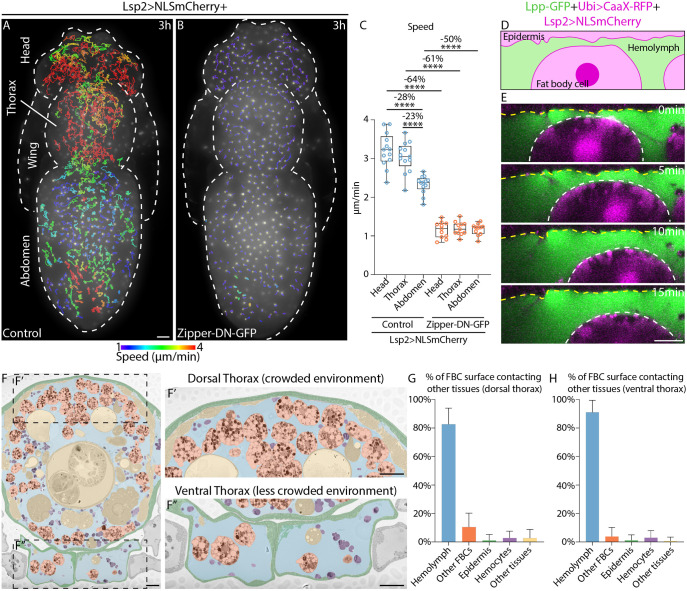
**FBCs patrol the pupa by swimming migration.** (A,B) Widefield time-lapse images of the dorsal view of pupae expressing Lsp2-Gal4 and UAS-NLS-mCherry (Lsp2>NLSmCherry) either in the control background (A) or with UAS-Zipper-DN-GFP (B). mCherry fluorescence is shown along with migration tracks (1.5–3 h long), which are colour-coded according to mean speed. Pupal structures are outlined with dashed lines. See Movie 1. (C) Quantification of mean FBC speed from A and B in the head, thorax and abdomen for control (*n*=13 pupae) and Zipper-DN-GFP (*n*=11 pupae). The median is plotted as a line inside the box. The box extends from the 25th to the 75th percentile, and the whole dataset is shown by the whiskers and dots. Percentage reductions in speed are shown for the indicated comparisons. ****P<0.0001 (ordinary one-way multiple comparisons ANOVA using a Šidák's test). (D,E) Schematic (D) and *XZ* view of confocal time-lapse images (E) of an FBC in a pupa expressing Lpp-GFP, CaaX-RFP under a ubiquitin promoter, Lsp2-Gal4 and UAS-NLS-mCherry (Lpp-GFP+Ubi>CaaX-RFP+Lsp2>NLSmCherry). Hemolymph, green (Lpp-GFP); epidermis, magenta (above yellow dashed line; CaaX-RFP); FBCs, faint magenta with bright magenta nuclei (white-dotted outline; NLS-mCherry). See Movie 2. Images shown are representative of ten cells. (F–F″) Methylene Blue-stained, transverse section of the thorax with false-coloured FBCs (orange), hemolymph (blue), epidermis (green), hemocytes (purple) and other tissues (yellow). Dashed boxes in F indicated regions shown as magnified views in F′ and F″. (G,H) Quantification of percentage of FBC surface contacting other tissues in dorsal (G; *n*=424 cell outlines from six pupae) and ventral (H; *n*=265 cell outlines from eight pupae) thorax as shown in F′ and F″. Data are presented as the mean+s.d. Scale bars: 100 µm (A,B), 20 µm (E), 50 µm (F–F″).

To establish a high-throughput model of non-directed swimming cell migration, we first characterized the general migration behaviour of FBCs in the pupal body during normal development. For this, we focussed on the population of FBCs derived from the mesoderm during embryogenesis – the ‘larval fat body population’ – rather than the adult fat body population, which undergoes a distinct, developmental migration during metamorphosis (Lei et al., 2023; Tsuyama et al., 2023). We expressed mCherry with a nuclear localization signal (NLS; UAS-NLS-mCherry) exclusively in FBCs using the FBC-specific Lsp2-Gal4 driver (Deutsch et al., 1989) and automatically tracked the large polyploid nuclei of these giant cells in two dimensions (2D) using *Z* projections of 3 h-long movies acquired on a widefield microscope. This new automatic nuclear tracking assay allowed us to assess the migration behaviour (including track path, speed, directionality and total displacement) of ∼300 cells per pupa in dozens of pupae per condition by analysing continuous tracks of 1.5–3 h length. We also used this to measure the mean FBC speed for each pupa from the mean speed of all tracked FBCs. We found that in pupae at 16 h after pupa formation (APF), in the absence of any wound, FBCs were highly migratory (Fig. 1A; Movie 1A). In the head and thorax, FBCs migrated at a high speed (mean speed of 3.2 µm/min and 3 µm/min, respectively; Fig. 1A,C). This fast migration led to the displacement of FBCs inside the pupa over very long distances of up to 153 µm in 3 h (Fig. S1A). In contrast, in the abdomen, FBCs migrated with a significantly lower speed (mean speed of 2.3 µm/min; Fig. 1A,C; Movie 1A) and lower straightness (Fig. S1B) than in the head and thorax. The mean speed for individual FBC tracks was highly variable, ranging from 1.2 µm/min to 5.3 µm/min in the head and thorax (Fig. S1C). Moreover, individual FBC tracks frequently alternated between periods of slower and faster migration (Fig. S1D–F; mean maximum speed of 6–7 µm/min, highest maximum speed of 11–13 µm/min). This resembles a stop-and-go or run-and-tumble mode of migration resulting in random walk (Zhang et al., 2023; Miller et al., 2002).

Next, we wanted to determine whether this motility is indeed due to active migration rather than passive movement, such as that generated by potential hemolymph flows. In wild-type pupae, neighbouring FBCs usually moved independently in different directions (Movie 1A). This observation argues against the presence of strong hemolymph flows. To assess further to what extent the observed cell movement is active, we expressed dominant-negative (DN) GFP-tagged Zipper (Zipper-DN-GFP) specifically in FBCs. Zipper is the heavy chain of myosin II, known to be essential for actomyosin contractions and cell migration in other cell types (Majumder et al., 2012; Yolland et al., 2019). Expression of Zipper-DN-GFP in FBCs led to a strong, significant reduction in migration speed (50–64% lower) in all three compartments (Fig. 1A–C; Movie 1). We suspect that the remaining movement might be due to incomplete block of gene function or/and hemolymph flows. This suggests that the observed movement of FBCs in all areas of the pupal body is largely driven by active cell migration.

### FBCs migrate in the hemolymph by swimming migration

Having found that there are many fast migrating FBCs at a high density in the dorsal head and thorax, we decided to use this region to study the mechanism of FBC migration further using confocal microscopy. To see whether FBCs use contact-independent swimming migration in the crowded regions of the head and thorax, we first visualized the migration of FBCs in relation to the epidermis [labelled with ubiquitously expressed RFP fused to a CaaX motif (CaaX-RFP)] and the hemolymph [labelled with an Lpp (also known as Apolpp)-GFP fusion protein]. We observed that in most cases, FBCs (labelled strongly with Lsp2-Gal4 and UAS-NLS-mCherry and weakly with CaaX-RFP) did not make any close contact with the epidermis during migration (Fig. 1D,E; Movie 2). The cells always kept their spherical shape rather than flattening, and moved within the hemolymph, confirming that FBCs swim independently of cell–epidermis contacts. Moreover, we quantified in Methylene Blue-stained serial sections of the thorax how much of the FBC surface was in contact with hemolymph versus various tissues. In the dorsal thorax, 82% of the surface of FBCs was surrounded by hemolymph, with only limited contact to other FBCs, the epidermis, hemocytes (*Drosophila* macrophages) or other pupal tissues (Fig. 1F,F′ and G). It is possible that these transient, minor cell–cell contacts might contribute to FBC migration in these crowded regions. However, in the much less crowded ventral thorax, where many FBCs are exclusively surrounded by hemolymph (Fig. 1F″,H), FBCs migrate at speeds that are very similar (mean speed of ∼3.5 µm/min; Franz et al., 2018) to that of FBCs in the head and thorax (Fig. 1C). Taken together, our results indicate that the migration of FBCs in the dorsal head and thorax is by swimming migration with limited cell–cell contact.

### Random-walk FBC migration is driven by periodic cortical actin waves at the cell rear

In amoeboid migration and *in vitro* swimming migration, rearward cortical actin flows usually generate the main force to power migration (Ruprecht et al., 2015; Lin et al., 2022, 2015; Aoun et al., 2020; Bergert et al., 2015). We previously described that directed migration of FBCs towards wounds in the ventral thorax involves periodic cortical actin waves (Franz et al., 2018). We next tested whether, in the absence of a wound, non-directed migration of FBCs in the dorsal head and thorax is associated with similar actin waves using confocal microscopy. Unfortunately, light scattering due to the high lipid content of FBCs prevented us from imaging the bottom half of the cells. Hence, we generally only imaged the top half of FBCs when using confocal microscopy. Although this limited the analysis to 2D, this is not a major problem because we imaged FBCs located under the epidermis, which mainly move in the *XY* axis. Moreover, we usually excluded cells that had significant movement within the *Z* axis.

**Fig. 2.**
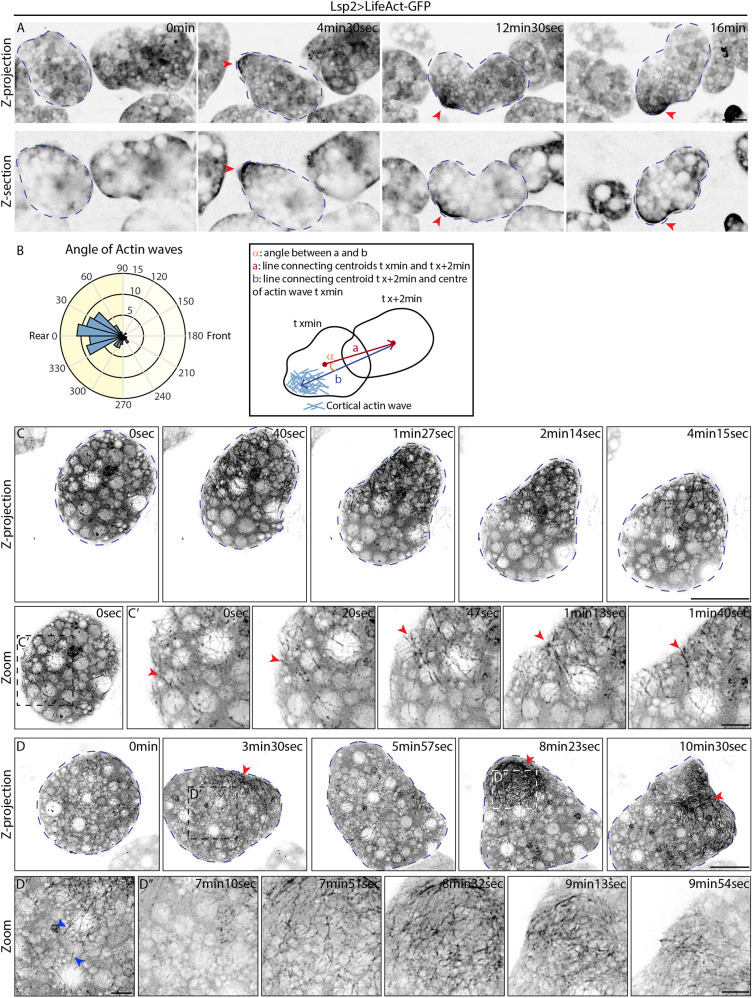
**Cortical actin waves in the cell rear are involved in FBC migration.** (A) Confocal time-lapse images of FBCs expressing Lsp2-Gal4 and UAS-LifeAct-GFP (Lsp2>LifeAct-GFP). LifeAct-GFP is shown in inverted greyscale. Actin waves are marked by red arrowheads; an FBC is indicated by blue dashed outlines. See Movie 4. (B) Rose plot of the location of actin waves within cells, measured as the angle between the line connecting the wave centre to the cell centroid at peak time point and the line of the cell rear–front axis (that is, the line through centroids at time point of wave peak and 2 min later; see diagram on the right). *n*=64 waves from 14 cells in five pupae. (C–D″) Super-resolution time-lapse images of FBCs expressing Lsp2-Gal4 and UAS-LifeAct-GFP. LifeAct-GFP is shown in inverted greyscale. In C and D, FBCs are indicated by blue dashed outlines. Dashed boxes indicate the location of magnified views shown in C′, D′ and D″. Red arrowheads in C′ highlight actin flow. Red arrowheads in D indicate actin mesh. Blue arrowheads in D′ highlight long actin bundles. Images shown are representative of 15 cells. See Movie 6A,B. Scale bars: 20 µm (A,C,D), 5 µm (C′,D′,D″).

To visualize actin dynamics, we performed *in vivo* live imaging using various actin markers. Several previous studies, including one in *Drosophila* nurse cells (Spracklen et al., 2014), have shown that the expression of some actin markers can negatively affect the actin network. Similarly, we found that expression of the actin probes GMA-GFP (Edwards et al., 1997) and Utrophin-GFP (Burkel et al., 2007), whilst labelling the actin network efficiently (Movie 3), impacted FBC migration speed (Fig. S2A,C–E). However, speed was not significantly altered by expression of LifeAct-GFP (Fig. S2A,B,E). Using LifeAct-GFP to study actin dynamics in swimming FBCs, we found that periodic cortical actin waves were present in all FBCs in the pupal head (*n*=194 cells from 12 pupae imaged over 60 min; Fig. 2A and Movie 4, red arrowheads point to actin waves). The presence of fluorescent signal in non-cortical areas made it challenging to identify cortical actin waves by assessing changes in overall mean actin intensity. Hence, we found it more reliable to detect actin waves visually in the *Z* projections, noticeable as a local increase in cortical actin intensity followed by a decrease. Most times, these actin waves travelled a certain distance over the surface of the cell (86% travelling waves, 14% local pulses, *n*=342; for simplicity, travelling waves and pulses are hereafter jointly referred to as ‘actin waves’; Fig. S3A,B and Movie 5). We found that the waves consistently peaked at the rear of the cell (Fig. 2B; 91% of waves in rear half, front–rear axis determined from centroid displacement vector). This resulted in cell locomotion in the opposite direction.

To study the structure of the actin network in the waves in more detail, next we performed super-resolution *in vivo* live imaging. This revealed that waves were composed of a fibrous cortical actin network that sometimes clearly moved from the front towards the rear, resembling a rearward actin flow (Fig. 2C,C′, red arrowheads; Movie 6A). This was accompanied by a cell shape change from round to pear-shaped. In other cases, we observed a more complex behaviour of the actin network. Overall, we noticed two main features of the actin network. First, large parts of the network consisted of a meshwork of actin filaments that appeared to be highly dynamic as it travelled over the cell surface (Fig. 2D,D″, red arrowheads; Movie 6B). Second, we noticed very long actin bundles of up to 20 µm that connected actin networks on opposite sides of the cell to join them up (Fig. 2D,D′, blue arrowheads; Movie 6B). This resulted in the belt-like squeezing of the cell centre causing the other two sides of the cell that contained less actin to bulge outwards. Sometimes bulged-out cell areas were later retracted through new actin waves (Movie 6B).

Overall, the meshwork of actin bundles seemed highly dynamic and usually did not cover the entire cortex of the cell, with parts of the surface being seemingly devoid of actin bundles. Actin waves moved along the cell surface to nearby areas previously devoid of actin bundles (Fig. 2D″; Movie 6B). In contrast, Utrophin-GFP expression – which, as we mentioned before, reduced migration speed (Fig. S2A,D,E) – resulted in an unusually stable, dense actin meshwork. This meshwork covered most of the cortex, contained many thick actin bundles, and some actin was also found in circular structures deeper inside the cell (Fig. S2H; Movie 3C), which were not observed with LifeAct-GFP or GMA-GFP (Fig. S2F,G; Movie 3A,B). Taken together, these observations suggest that the FBC actin network has to be dynamic to allow efficient FBC migration.

Mesenchymal and amoeboid migration often involves the formation of protrusions in the cell front such as lamellipodia and blebs, respectively. We never observed any actin-rich lamellipodia in the cell front during FBC migration (Movies 4–6). Instead, swimming FBCs usually maintained a rounded cell shape at the cell front. We only very rarely saw small blebs forming on the cell surface in random locations, and these blebs did not appear to be related to the overall migration (Movies 4–6).

### The small Rho GTPase Rho1 is crucial for FBC migration by promoting actin wave formation

**Fig. 3.**
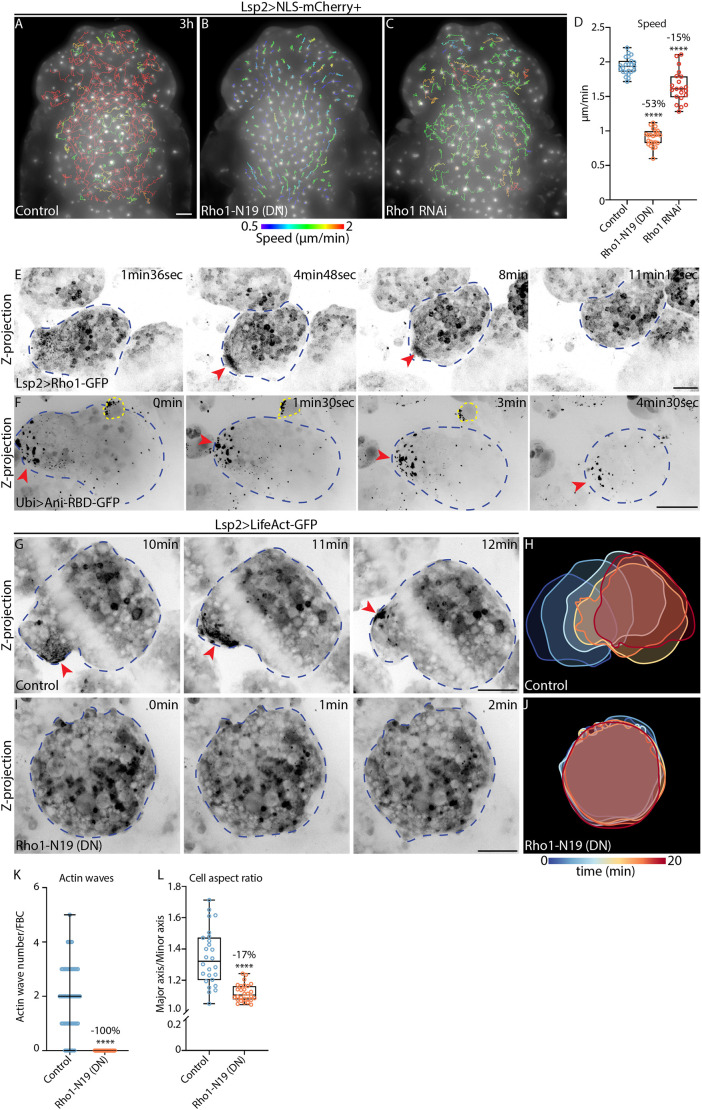
**Rho1 is needed for actin wave formation and cell deformations during swimming.** (A–C) Widefield time-lapse images of the dorsal head and thorax of pupae expressing Lsp2-Gal4 and UAS-NLS-mCherry (Lsp2>NLS-mCherry) either in the control background control (A) or with UAS-Rho1-N19 (B) or UAS-Rho1-RNAi (C). mCherry fluorescence is shown along with migration tracks (1.5–3 h long), which are colour-coded according to mean speed. See Movie 7A,B. (D) Quantification of mean FBC speed from A–C for control (*n*=23 pupae), Rho1-N19 (*n*=23 pupae) and Rho1-RNAi (*n*=21 pupae). Percentage reductions in speed relative to the control are shown. ****P<0.0001 (ordinary one-way multiple comparisons ANOVA using a Dunnett's test). (E,F) Confocal time-lapse images of FBCs in pupae expressing Lsp2-Gal4 and UAS-Rho1-GFP (E; Lsp2>Rho1-GFP), or Ani-RBD-GFP under control of the ubiquitin promoter (F; Ubi>Ani-RBD-GFP). GFP signal is shown in inverted greyscale. Red arrowheads highlight accumulation of Rho1-GFP (E) or Ani-RBD-GFP (F) at the cell rear. Blue dashed outlines mark FBCs; yellow dashed outline marks a hemocyte. Images are representative of 15 cells (E) or nine cells (F). See Movie 8A,B. (G–J) Confocal time-lapse images (G,I) and colour-coded time projections of cell outlines (H,J) from FBCs expressing Lsp2-Gal4 and UAS-LifeAct-GFP (Lsp2>LifeAct-GFP) in either the control background (G,H) or with UAS-Rho1-N19 (I,J). LifeAct-GFP is shown in inverted greyscale. Red arrowheads highlight an actin wave. Blue dashed outlines mark FBCs. Note the shadow of a cuticle fold seen in G. See Movie 9. (K) Quantification of actin wave numbers in FBCs from 20 min-long movies as in G and I. Control, *n*=59 cells from six pupae; Rho1-N19, *n*=52 cells from six pupae. Lines indicate median values and error bars show the range. Percentage reduction in wave number relative to the control is indicated. ****P<0.0001 (two-tailed Mann–Whitney test)*.* (L) Quantification of mean aspect ratio of FBCs as in G and I. Control, *n*=26 cells from six pupae); Rho1-N19, *n*=28 cells from six pupae. Percentage reduction in aspect ratio relative to the control is indicated. ****P<0.0001 (two-tailed unpaired *t*-test). Scale bars: 100 µm (A–C), 20 µm (E–G,I). For box and whisker plots in D and L, the median is plotted as a line inside the box. The box extends from the 25th to the 75th percentile, and the whole dataset is shown by the whiskers and dots.

Having found that the actin cytoskeleton likely plays a key role in swimming migration, we next decided to investigate potential regulators. For this, we took advantage of our newly developed automatic nuclear tracking assay, which allows assessment of the migration speed of thousands of cells. We started with the small Rho GTPase Rho1, *w*hich, like its mammalian orthologue RhoA, plays a key role during amoeboid migration by controlling the formation of a retrograde actin flow and inducing contractility at the cell rear (Lin et al., 2022; Kardash et al., 2010; Poincloux et al., 2011). Expression of a DN Rho1 (Rho1-N19) or RNA interference (RNAi) of Rho1 (Rho1-RNAi) specifically in FBCs resulted in a significant 53% or 15% reduction in FBC migration speed, respectively [Fig. 3A–D; Movie 7A,B; please note that, to allow higher throughput analysis, these pupae and those reported hereafter were imaged every 5 min instead of every 1.5 min (as was the case for pupae shown in Fig. 1A–C), hence resulting in lower mean speed values]. This suggests that Rho1 plays a key role in the swimming migration of FBCs.

We next studied the subcellular localization of Rho1 by expressing GFP-tagged Rho1 (UAS-Rho1-GFP) in FBCs using the Lsp2-Gal4 driver. We observed an accumulation of Rho1-GFP in the rear of migrating FBCs (Fig. 3E; Movie 8A) where actin waves were usually found. We also tested the Anillin (also known as Scraps) Rho1-GTP binding domain fused to GFP (Ani-RBD-GFP) under the control of the ubiquitin promoter, which has previously been used to assess the localization of active Rho1 (Munjal et al., 2015). Ani-RBD-GFP also accumulated in the rear of migrating FBCs (Fig. 3F; Movie 8B). This suggests that during FBC migration, Rho1 accumulates and gets activated at the cell rear, where it might control actin waves.

Next, we tested whether blocking of Rho1 function affects actin wave formation and cell deformations. In control FBCs, an average of two actin waves formed in 20 min (Fig. 3G,K; Movie 9A). In contrast, Rho1-N19 expression completely abolished actin wave formation (Fig. 3I,K; Movie 9B). Moreover, whereas control FBCs were more deformed with undulating circular cell shapes and displayed dynamic cell deformations (Fig. 3H,L; Movie 9A), Rho1-N19-expressing FBCs were more circular throughout the length of the movies and had a significantly lower mean aspect ratio (Fig. 3J,L; Movie 9B; mean aspect ratio calculated for each cell over 20 min). Thus, Rho1 is a key regulator of FBC swimming migration by controlling the formation of actin waves and regulating cell deformations.

### Actomyosin contractility drives FBC deformations during swimming migration

**Fig. 4.**
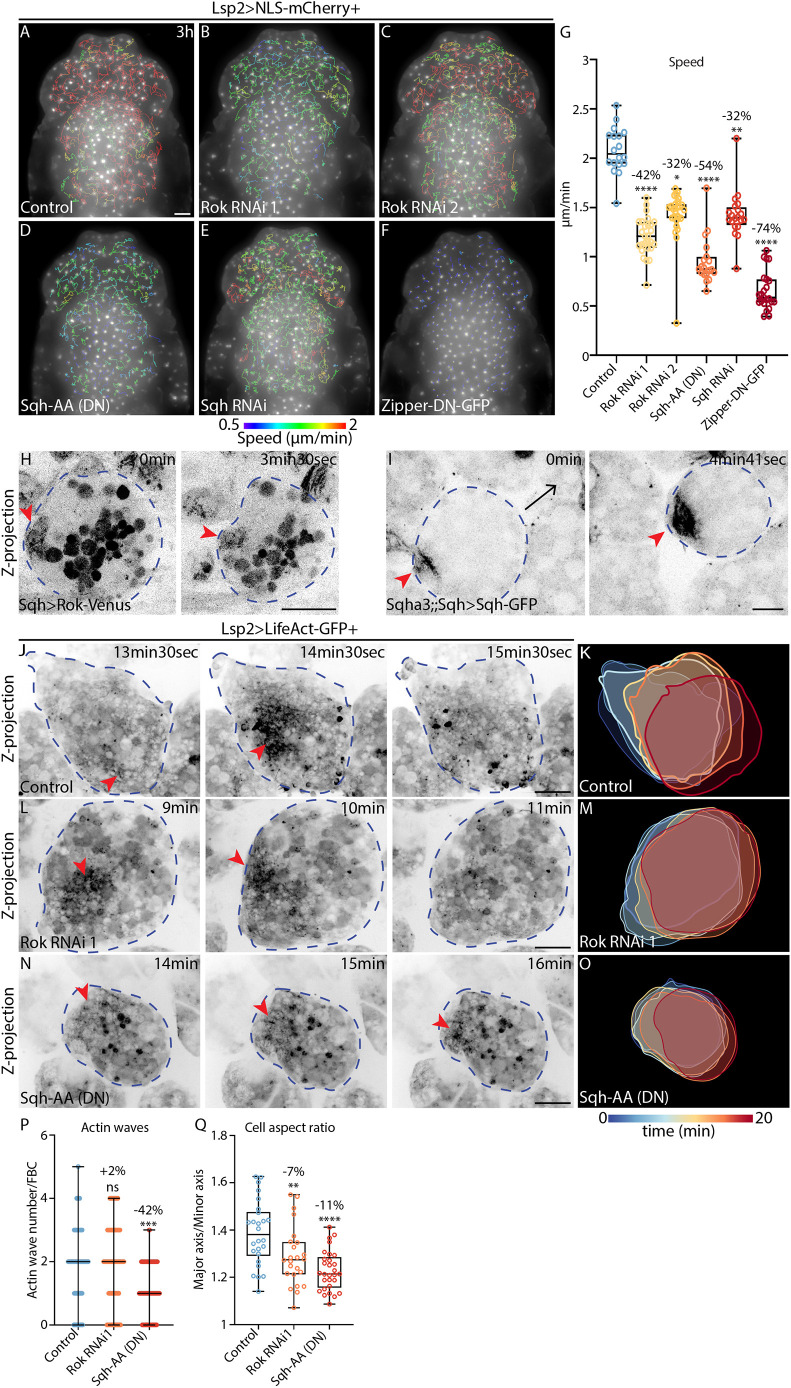
**Actomyosin contractility is essential for FBC deformations during swimming.** (A-F) Widefield time-lapse images of the dorsal head and thorax of pupae expressing Lsp2-Gal4 and UAS-NLS-mCherry (Lsp2>NLS-mCherry) either in the control background (A) or with UAS-Rok-RNAi 1 (B), UAS-Rok-RNAi 2 (C), UAS-Sqh-AA (DN) (D), UAS-Sqh-RNAi (E) or UAS-Zipper-DN-GFP (F). mCherry fluorescence is shown along with migration tracks (1.5–3 h long), which are colour-coded according to mean speed. See Movie 7C,D. (G) Quantification of mean FBC speed from A–F for control (*n*=18 pupae), Rok-RNAi 1 (*n*=27 pupae), Rok-RNAi 2 (*n*=27 pupae), Sqh-AA (DN) (*n*=19 pupae), Sqh-RNAi (*n*=20 pupae) and Zipper-DN-GFP (*n*=23 pupae). Percentage reductions in speed relative to the control are shown. *P=0.0128, **P=0.0061 and ****P<0.0001 (multiple comparisons Kruskal–Wallis test using Dunn's test). (H,I) Confocal time-lapse images of FBCs in pupae with expression of Rok^K116A^-Venus (H; Sqh>Rok^K116A^-Venus) or Sqh-GFP (I; Sqh^AX3^+Sqh>Sqh-GFP) driven by the *sqh* promoter. Fluorescence signals are shown in inverted greyscale. Blue dashed outlines mark FBCs. Red arrowheads highlight accumulation of Rok^K116A^-Venus (H) or Sqh-GFP (I) at the cell rear. Arrow in I shows the direction of migration. Images are representative of four cells (H) or 14 cells (I). See Movie 10A,B. (J–O) Confocal time-lapse images (J,L,N) and colour-coded time projections of cell outlines (K,M,O) from FBCs expressing Lsp2-Gal4 with UAS-LifeAct-GFP (Lsp2>LifeAct-GFP) either in the control background (J,K) or with UAS-Rok-RNAi 1 (L,M) or UAS-Sqh-AA (N,O). LifeAct-GFP is shown in inverted greyscale. Red arrowheads highlight actin waves. Blue dashed outlines mark FBCs. See Movie 11. (P) Quantification of actin wave numbers in FBCs from 20 min-long movies as in J,L and N. Control, *n*=55 cells from five pupae; Rok-RNAi 1, *n*=62 cells from five pupae; Sqh-AA (DN), *n*=64 cells from six pupae. Lines indicate median values and error bars show the range. Percentage reduction in wave number relative to the control is indicated. ***P=0.0006; ns, *P*>0.9999 (multiple comparisons Kruskal–Wallis test using a Dunn's test). (Q) Quantification of mean aspect ratio of FBCs as in J, L and N. Control, *n*=26 cells from five pupae; Rok-RNAi 1, *n*=24 cells from five pupae; Sqh-AA (DN), *n*=29 cells from six pupae. Percentage reduction in aspect ratio relative to the control is indicated. **P=0.0075, ****P<0.0001 (ordinary one-way multiple comparisons ANOVA using a Dunnett's test). Scale bars: 100 µm (A–F), 20 µm (H–J,L,N). For box and whisker plots in G and Q, the median is plotted as a line inside the box. The box extends from the 25th to the 75th percentile, and the whole dataset is shown by the whiskers and dots.

RhoA is generally known to regulate actomyosin contractility through Rho kinases (ROCKs) and myosin II (Ridley, 2001, 2015). We observed a strong reduction in FBC migration speed when we blocked Rok, the *Drosophila* homologue of ROCKs (using two different RNAi lines, Rok-RNAi 1 and 2); myosin II light chain [using DN Sqh (Sqh-AA) and an RNAi line (Sqh-RNAi)]; and myosin II heavy chain (using Zipper-DN-GFP) (Fig. 4A–G; Movie 7C,D). This suggests that Rok and myosin II play an important role in FBC migration and points further at the importance of Rho1 in FBC migration.

Next, we studied the subcellular localization of Rok and myosin II during FBC migration. We used lines in which expression of Rok^K116A^-Venus or Sqh-GFP is driven in several cell types by the *sqh* promoter. Rok^K116A^ is a mutant variant with a disrupted catalytic activity to avoid high levels of Rok activity in the cell. The Sqh-GFP line used has a *sqh* mutant background to avoid potential negative side effects caused by Sqh overexpression. Strikingly, Rok^K116A^-Venus and Sqh-GFP accumulated in the cortex at the rear of migrating FBCs (Fig. 4H,I; Movie 10), where we usually also found actin waves.

We then examined whether blocking Rok and Sqh function affects actin wave formation in FBCs. Interestingly, in both cases FBCs still had actin waves (Fig. 4J,L,N,P; Movie 11). In contrast to the effects of Rok-RNAi, the expression of Sqh-AA significantly decreased the number of actin waves by 42% (Fig. 4P). This difference between Rok-RNAi and Sqh-AA, both in reducing speed (Fig. 4G) and in reducing actin waves, could be explained either by myosin II being activated by Rok as well as by another kinase, or by the loss-of-function effect of Sqh-AA being stronger than that of Rok-RNAi. Taken together, these data suggest that actomyosin contraction, regulated by myosin II, at least in part, contributes to the regulation of actin waves.

Next, we hypothesized that myosin-driven contraction of the cortical actin during actin wave formation would be particularly important for inducing cell deformations by locally pulling the cell surface inwards. Indeed, we found that loss of function of Rok and myosin II significantly reduced the aspect ratio of FBCs (Fig. 4K,M,O,Q; Movie 11).

In summary, actomyosin contractions in the cell rear regulated by myosin II are crucial for FBC deformations and contribute, to a lesser extent, to actin wave regulation during swimming migration.

### Actin wave formation during *in vivo* swimming migration requires Dia-driven actin polymerization

**Fig. 5.**
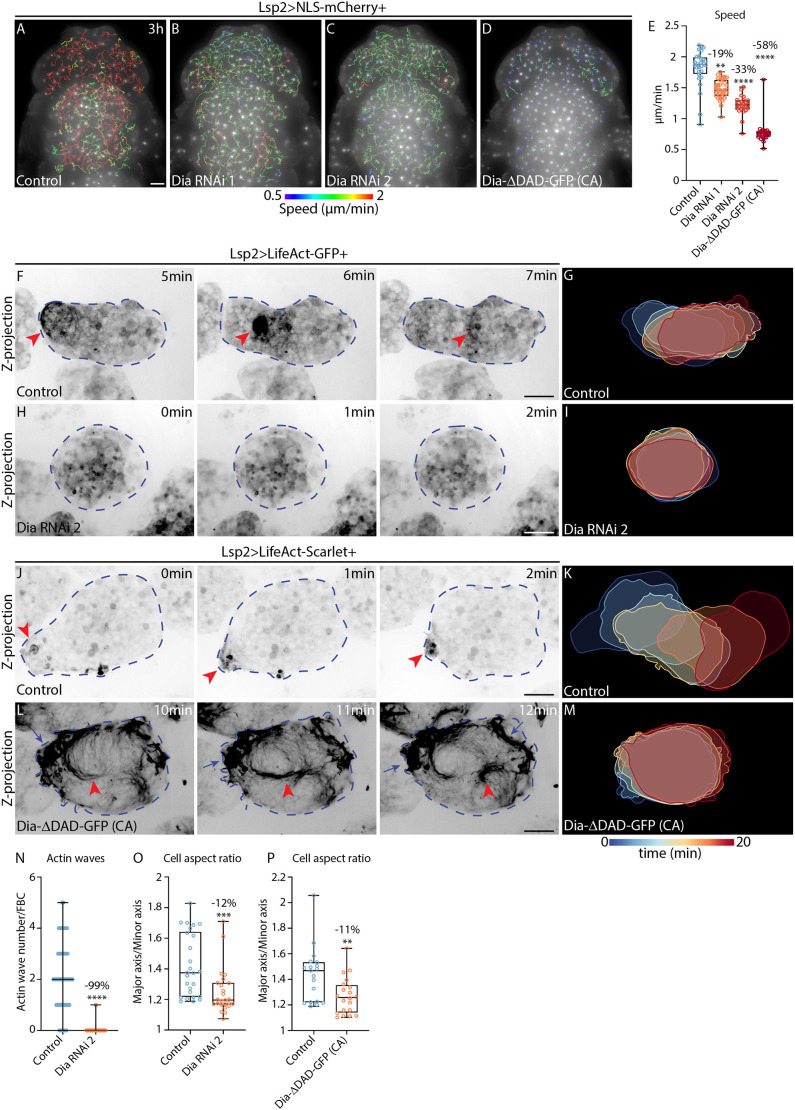
**Actin wave formation in FBC swimming is regulated by Dia.** (A–D) Widefield time-lapse images of the dorsal head and thorax of pupae expressing Lsp2-Gal4 and UAS-NLS-mCherry (Lsp2>NLS-mCherry) either in the control background (A) or with UAS-Dia-RNAi 1 (B), UAS-Dia-RNAi 2 (C) or UAS-Dia-ΔDAD-GFP (CA) (D). mCherry fluorescence is shown along with migration tracks (1.5–3 h long), which are colour-coded according to mean speed. See Movie 7E,F. (E) Quantification of mean FBC speed from A–D for control (*n*=28 pupae), Dia-RNAi 1 (*n*=35 pupae), Dia-RNAi 2 (*n*=25 pupae) and Dia-ΔDAD-GFP (CA) (*n*=26 pupae). Percentage reductions in speed relative to the control are shown. **P=0.0065, ****P<0.0001 (multiple comparisons Kruskal–Wallis test using a Dunn's test). (F–M) Confocal time-lapse images (F,H,J,L) and colour-coded time projections of cell outlines (G,I,K,M) from FBCs expressing Lsp2-Gal4 and UAS-LifeAct-GFP (Lsp2>LifeAct-GFP) in the control background (F,G) or with UAS-Dia-RNAi 2 (H,I), or expressing Lsp2-Gal4 and UAS-LifeAct-Scarlet (Lsp2>LifeAct-Scarlet) in the control background (J,K) or with UAS-Dia-ΔDAD-GFP (L,M). LifeAct-GFP (F,H) and LifeAct-Scarlet (J,L) are shown in inverted greyscale. Red arrowheads highlight actin waves (F,J) or dynamic actin swirls (L). Blue arrows highlight cell surface protrusions containing concentrated actin (L). Blue dashed outlines mark FBCs. See Movies 12 and 13. (N) Quantification of actin wave numbers in FBCs from 20 min-long movies as in F and H. Control, *n*=55 cells from five pupae; Dia-RNAi 2, *n*=59 cells from six pupae. Lines indicate median values and error bars show the range. Percentage reduction in wave number relative to the control is indicated. ****P<0.0001 (two-tailed Mann–Whitney test). (O,P) Quantification of mean aspect ratio of FBCs as in F and H (O) or as in J and L (P). Control, *n*=26 cells from five pupae; Dia-RNAi 2, *n*=25 cells from six pupae (O). Control, *n*=19 cells from four pupae; Dia-ΔDAD-GFP, *n*=20 cells from four pupae (P). Percentage reduction in aspect ratio relative to the control is indicated. **P=0.0083, ***P=0.0002 (two-tailed Mann–Whitney test). Scale bars, 100 µm (A–D), 20 µm (F,H,J,L). For box and whisker plots in E, O and P, the median is plotted as a line inside the box. The box extends from the 25th to the 75th percentile, and the whole dataset is shown by the whiskers and dots.

In mesenchymal and amoeboid cell migration, in addition to regulating actomyosin contraction in the cell rear, RhoA is known to induce actin polymerization via Dia-related formins (Ridley, 2001, 2015). Indeed, altering Dia function through FBC-specific expression of two different RNAi lines (Dia-RNAi 1 and 2) or of constitutively active (CA) Dia (Dia-ΔDAD-GFP) resulted in a significant moderate-to-strong reduction in FBC migration speed (Fig. 5A–E; Movie 7E,F). This suggested that Dia is required for FBC migration. Moreover, Dia-RNAi strongly reduced actin wave numbers (Fig. 5F,H,N; Movie 12) and resulted in rounder cell shapes (Fig. 5G,I,O). In contrast, Dia overactivation resulted in a strongly increased cortical actin meshwork covering most of the cortex and containing many swirls of aligned actin bundles (Fig. 5J,L; Movie 13). These highly dynamic actin swirls moved along the whole FBC cortex and appeared different to actin waves in the control (Movie 13). Dia overactivation also induced the formation of some type of cell surface protrusions containing strongly concentrated actin (Fig. 5L). The overall cell shape was also impacted upon Dia overactivation, with FBCs being more circular throughout time and deforming less than in the control (Fig. 5K,M,P).

These data suggest that actin polymerization regulated by Dia is crucial for actin wave formation in migrating FBCs. Moreover, our Dia overactivation data are consistent with Utrophin-GFP expression stabilizing the actin network and reducing migration speed (Fig. S2A,D–F,H; Movie 3). This suggests that the cortical actin mesh needs to be dynamic and tightly controlled to allow normal FBC migration.

### Cdc42 and Rac1 are key regulators of FBC swimming migration

**Fig. 6.**
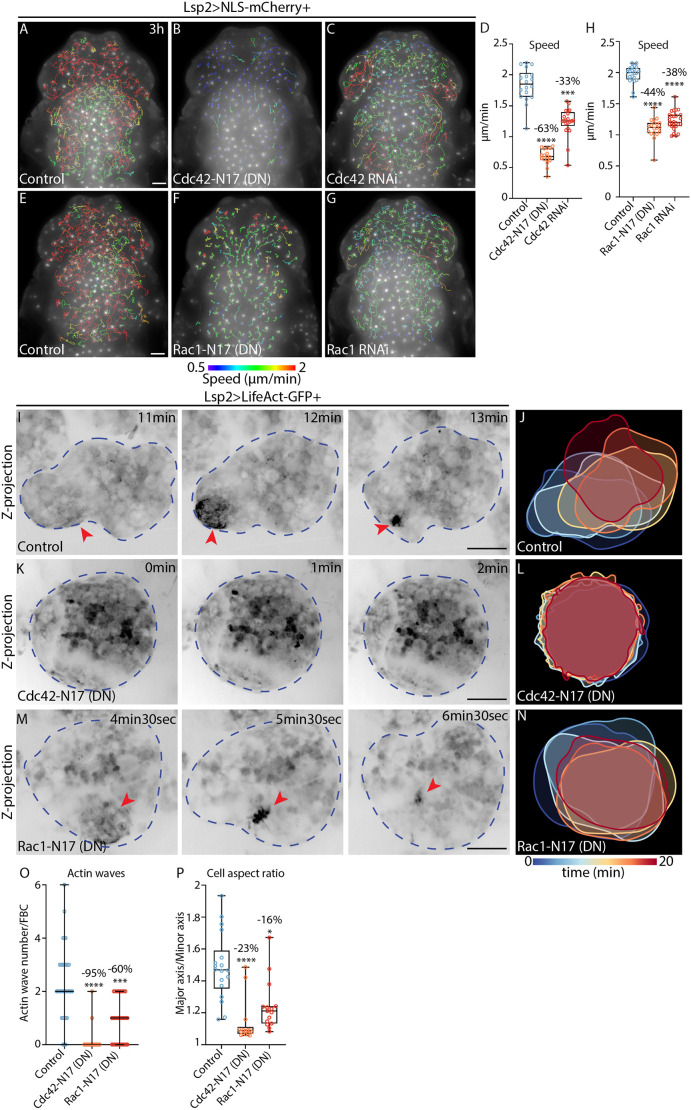
**Cdc42 and Rac1 control FBC migration through the regulation of actin waves and cell deformations.** (A–C,E–G) Widefield time-lapse images of the dorsal head and thorax of pupae expressing Lsp2-Gal4 and UAS-NLS-mCherry (Lsp2>NLS-mCherry) in the control background (A,E) or with UAS-Cdc42-N17 (DN) (B), UAS-Cdc42-RNAi (C), UAS-Rac1-N17 (DN) (F) or UAS-Rac1-RNAi (G). mCherry fluorescence is shown along with migration tracks (1.5–3 h long), which are colour-coded according to mean speed. See Movie 7G,H. (D,H) Quantification of mean FBC speed from A–C (D) and E–G (H). Control, *n*=18 pupae; Cdc42-N17, *n*=17 pupae; Cdc42-RNAi, *n*=21 pupae (D). Control, *n*=22 pupae; Rac1-N17, *n*=19 pupae; Rac1-RNAi, *n*=26 pupae (H). Percentage reductions in speed relative to the control are shown. ***P=0.0008, ****P<0.0001 (multiple comparisons Kruskal–Wallis test using a Dunn's test). (I–N) Confocal time-lapse images (I,K,M) and colour-coded time projections of cell outlines (J,L,N) from FBCs expressing Lsp2-Gal4 with UAS-LifeAct-GFP (Lsp2>LifeAct-GFP) in the control background (I,J) or with either UAS-Cdc42-N17 (K,L) or UAS-Rac1-N17 (M,N). LifeAct-GFP is shown in inverted greyscale. Red arrowheads highlight actin waves. Blue dashed outlines mark FBCs. See Movie 14. (O) Quantification of actin wave numbers in FBCs from 20 min-long movies as in I,K,M. Control, *n*=34 cells from four pupae; Cdc42-N17, *n*=26 cells from three pupae; Rac1-N17, *n*=36 cells from three pupae. Lines indicate median values and error bars show the range. Percentage reduction in wave number relative to the control is indicated. ***P=0.0001, ****P<0.0001 (multiple comparisons Kruskal–Wallis test using a Dunn's test). (P) Quantification of mean aspect ratio of FBCs as in I,K,M. Control, *n*=18 cells from four pupae; Cdc42-N17, *n*=14 cells from three pupae; Rac1-N17, *n*=15 cells from three pupae. Percentage reduction in aspect ratio relative to the control is indicated. *P=0.0141, ****P<0.0001 (multiple comparisons Kruskal–Wallis test using a Dunn's test). Scale bars: 100 µm (A–G), 20 µm (I,K,M). For box and whisker plots in D, H and P, the median is plotted as a line inside the box. The box extends from the 25th to the 75th percentile, and the whole dataset is shown by the whiskers and dots.

Since amoeboid migration usually does not involve lamellipodia or filopodia, it is generally believed that Rac1, Cdc42 and branched actin formation are mostly dispensable. Interestingly, we found that FBC-specific expression of DN Cdc42 (Cdc42-N17) and Rac1 (Rac1-N17), as well as RNAi of Cdc42 (Cdc42-RNAi) and Rac1 (Rac1-RNAi), strongly decreased migration speed of FBCs (Fig. 6A–H; Movie 7G,H). This suggests, that, in addition to Rho1, Cdc42 and Rac1 are also key regulators of the swimming migration of FBCs.

Moreover, we found that Cdc42-N17 expression (Fig. 6I,K,O; Movie 14A,B) and Cdc42-RNAi (Fig. S4A) decreased the number of actin waves in FBCs dramatically, which led to cell rounding (Fig. 6J,L,P; Fig. S4B). Rac1-N17 expression or Rac1-RNAi also resulted in a moderate reduction in the number of actin waves (Fig. 6I,M,O; Movie 14A,C and Fig. S4A) and cell rounding (Fig. 6J,N,P; Fig. S4B), although the effects were less severe than upon Cdc42-N17 expression or Cdc42 RNAi. Thus, Cdc42 and Rac1 are both major regulators of actin wave formation and cell shape during FBC migration.

In mesenchymal migration, Cdc42 and Rac1 are known to regulate Arp2/3-driven branched actin formation via Wasp (also known as WAS in mammals) and the Scar/Wave complex (Ridley, 2001, 2015). Moreover, we had noticed that when observed using super-resolution microscopy, the actin network in FBCs appeared to be formed by a mesh (Fig. 2C,D). In agreement with this, we found that RNAi of Wasp as well as of Arp2 or Arp3 led to a significant decrease in FBC migration speed (Fig. S5A–I). Moreover, RNAi of Wasp RNAi and of Arp2 reduced the number of actin waves (Fig. S5J,K). This suggests that Wasp and the Arp2/3 complex are involved in FBC migration.

### Stochastic contractile actin waves at the rear can induce cell elongation or cell rounding

**Fig. 7.**
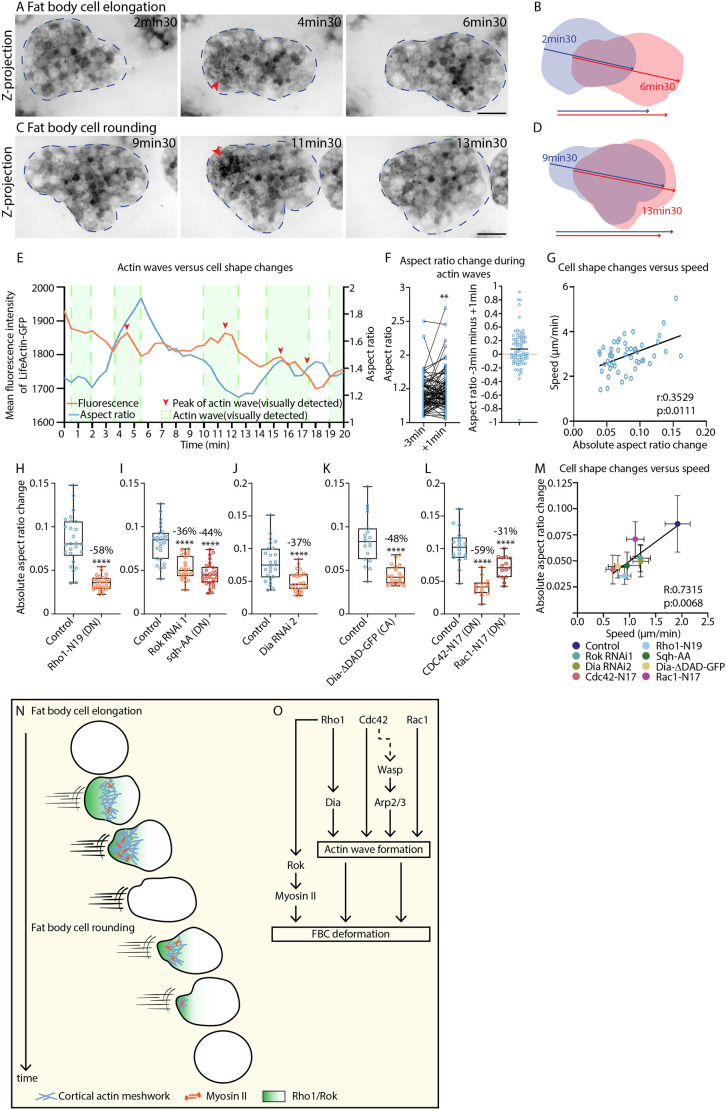
**Cell deformations induced by actin waves at the rear are the key driver of swimming migration.** (A–D) Representative confocal time-lapse images of FBCs expressing Lsp2-Gal4 and UAS-LifeAct-GFP (A,C), and corresponding wave-induced cell shape changes (B,D). A and B show an example of cell elongation; C and D show an example of cell rounding through rear retraction. LifeAct-GFP is shown in inverted greyscale. Red arrowheads highlight actin waves. Blue dashed outlines mark FBCs. Blue and red arrows indicate the front–rear axis before and after the wave, respectively. Scale bars: 20 µm. See Movie 15. (E) Quantification of mean fluorescence intensity of LifeAct-GFP and cell aspect ratio of the FBC from Movie 15. Visually detected actin waves and peaks are marked by green areas and red arrowheads, respectively. (F) Aspect ratio changes of FBCs expressing Lsp2-Gal4 and UAS-LifeAct-GFP, from 3 min before wave peak to 1 min after peak, measured from confocal time-lapse series (*n*=83 waves from 20 cells across six pupae). The left graph shows aspect ratio at −3 min and 1 min and the right graph shows the aspect ratio difference (aspect ratio +1 min minus aspect ratio −3 min) for each wave. Horizontal line marks the mean. **P=0.0096 (two-tailed Wilcoxon test). (G) Mean absolute aspect ratio changes as a function of mean speed of FBCs expressing Lsp2-Gal4 and UAS-LifeAct-GFP, taken from confocal time-lapse series (mean for each cell from 20 min tracking at 1 min time intervals; *n*=51 cells from 10 pupae). Line shows the simple linear regression. Spearman correlation coefficient (r) and *P*-value (p) are shown. (H–L) Quantification of absolute aspect ratio change over time of FBCs expressing Lsp2-Gal4 and UAS-LifeAct-GFP (H) in the control background (*n*=26 cells from six pupae) or with UAS-Rho1-N19 (*n*=52 cells from six pupae); (I) in the control background (*n*=26 cells from five pupae) or with UAS-Rok-RNAi 1 (*n*=24 cells from five pupae) or UAS-Sqh-AA (DN) (*n*=29 cells from six pupae); (J) in the control background (*n*=26 cells from five pupae) or with Dia-RNAi 2 (*n*=25 cells from six pupae); (K) in the control background (*n*=19 cells from four pupae) or with Dia-ΔDAD-GFP (*n*=20 cells from four pupae); (L) or in the control background (*n*=18 cells from four pupae) or with Cdc42-N17 (*n*=14 cells from three pupae) or Rac1-N17 (*n*=15 cells from three pupae). Percentage aspect ratio changed compared to control is shown. The median is plotted as a line inside the box. The box extends from the 25th to the 75th percentile, and the whole dataset is shown by the whiskers and dots. ****P<0.0001 (two-tailed unpaired *t*-test in H,J and K; ordinary one-way multiple comparisons ANOVA with a Dunnett's test in I and L). (M) Absolute cell aspect ratio changes as a function of mean speed. Mean FBC speed values of pupae expressing Lsp2-Gal4 and UAS-NLS-mCherry in the control background (pooled from Figs 3D, 4G, 5E, 6D and 6H) or with UAS-Rho1-N19 (from Fig. 3D), UAS-Rok RNAi 1 (from Fig. 4G), UAS-Sqh-AA (from Fig. 4G), UAS-Dia RNAi 2 (from Fig. 5E), UAS-Dia-ΔDAD-GFP (from Fig. 5E), UAS-Cdc42-N17 (from Fig. 6D) or UAS-Rac1-N17 (from Fig. 6H). Absolute aspect ratio change values of pupae expressing Lsp2-Gal4 and UAS-LifeAct-GFP, or Lsp2-Gal4 and UAS-LifeAct-Scarlet, in the control background (pooled from H–L) or with UAS-Rho1-N19 (from H), UAS-Rok RNAi1 (from I), UAS-Sqh-AA (from I), UAS-Dia RNAi 2 (from J), UAS-Dia-ΔDAD-GFP (from K), UAS-Cdc42-N17 (from L) or UAS-Rac1-N17 (from L). Line shows simple linear regression. Error bars show s.d. Pearson correlation coefficient (R) and *P*-value (p) are shown. (N,O) Proposed model for FBC swimming migration (N) and molecular regulation (O). A contractile actomyosin wave induces cell elongation, resulting in propulsion. This is followed by a second wave retracting the rear, resulting in cell rounding.

Having found that actin waves are key for FBC migration, we next wanted to investigate how these waves drive cell locomotion by looking at the effects that waves have on cell shapes. Although actin waves formed very consistently at the rear of FBCs, resulting in locomotion in the opposite direction (Fig. 2B), the effects that actin waves had on cell shape appeared to be very complex and highly variable. Sometimes waves led to a local, belt-like compression of the cell surface that moved in a peristaltic manner to the rear [Movie 15, wave at 16:00–17:30 (min:s)]. Some waves induced a cell elongation [increase in aspect ratio; Fig. 7A,B,E and Movie 15, wave at 3:30–5:30 (min:s); and Movie 4 and Fig. S6A, waves at 4:00–5:30, 9:00–10:30, 13:30–15:00 and 16:00–17.30 (min:s)]. Other waves had the opposite effect and led to cell rounding [decrease in aspect ratio; Fig. 7C–E and Movie 15, wave at 10:00–12:30 (min:s); and Movie 4 and Fig. S6A, waves at 6:30–7:30 and 12:00–13:00 (mins:s)].

We initially hypothesized that contractile actin waves at the cell rear might cause cell elongations in the opposite direction – resulting in cell propulsion – which would finally be reverted through passive cell rounding. To test whether aspect ratio was indeed correlated with speed (elongated cells being faster), we performed quantitative analysis of cell shape and speed dynamics. We tracked and segmented Lifeact-GFP-expressing cells over 20–60 min, visually identified actin waves, and measured cell aspect ratio and speed every minute. When using all measurements (irrespective of timing compared to actin waves), there was only a very poor correlation between speed and aspect ratio (Fig. S6B). Moreover, to test more specifically whether actin waves induce cell elongation, we synchronized the measurements for all waves (actin wave peaks set to 0 min) and compared aspect ratio measurements from 5 min before the peak (−5 min) to 5 min after the peak (+5 min). This revealed only a small gradual increase in mean aspect ratio from −3 min to +1 min (Fig. S6C). Moreover, when we compared how the aspect ratio changed from −3 min to +1 min for each wave, we found that only 54% of waves led to an increase in aspect ratio, whereas 46% led to a decrease (Fig. 7F). Surprisingly, these results suggest that actin waves are almost as likely to induce cell elongation as cell rounding. However, the extent of the increases in aspect ratio induced by waves was higher than the extent of the induced decreases (39% of waves induced an increase in aspect ratio of over 0.15, whereas only 12% induced a decrease of over 0.15, *n*=83). This explains why we find that on average actin waves induce a slight net increase in aspect ratio.

### Cell deformations induced by actin waves at the rear are the key driver of swimming migration

Next, we hypothesized that actin waves might induce either active cell elongation or active cell rounding, both of which might be important for FBC migration. Interestingly, when looking at all our measurements, irrespective of timing compared to actin waves, we found that increases in aspect ratio showed a weak positive correlation with speed, whereas decreases in aspect ratio showed a weak negative correlation with speed (Fig. S6D,E). This suggests that positive and negative aspect ratio changes might be equally important for FBC migration. Indeed, we found that mean changes in absolute aspect ratio (calculated for each cell from the absolute changes in aspect ratio every minute over 20 min) showed a moderately strong positive correlation with mean speed (Fig. 7G). This suggests that the cells undergoing repetitive cycles of stronger cell elongation and stronger cell rounding are faster than cells undergoing cycles of weaker cell elongation and weaker cell rounding. This also means that cell deformations in general are key for driving FBC swimming migration.

Finally, we tested whether these cell deformations were caused by contractile actin waves. Indeed, we found that disrupting the gene function of the key regulators of actin wave formation and contraction – Rho1, Rok, myosin II, Dia, Rac1 and Cdc42 – strongly reduced mean absolute aspect ratio changes (Fig. 7H–L; Fig. S4C). Moreover, in all of these genetic manipulations that affect FBC migration, mean absolute aspect ratio changes directly correlated with migration speed (Fig. 7M). Taken together, these observations suggest that contractile actin waves at the cell rear induce cell deformations, including both cell elongation and cell rounding, which are a major driver of FBC migration. Interestingly, a computational model of amoeboid blebbing migration has suggested that non-reciprocal cell shape changes can theoretically drive force transmission in amoeboid migration (Lim et al., 2013). Our *in vivo* data provide some support of this model.

## DISCUSSION

In this study, we use *Drosophila* FBCs as the first physiological model system of swimming migration to dissect the molecular mechanism underlying swimming. For this, we combine high-throughput cell tracking with high- and super-resolution microscopy, as well as quantitative analysis of cell shape and speed dynamics. We show that, in the absence of wounds, pupal FBCs actively swim by non-directed random walk to patrol the pupal body at high speed. Swimming migration is driven by the stochastic formation of actin waves that travel to the cell rear. This requires Dia-driven actin polymerization, Arp2/3-driven actin branching as well as Rho1, Rac1 and Cdc42. Rok- and myosin-dependent contraction of these actin waves generates forces at the cell rear that lead to cell surface compression and cell deformation (Fig. 7N,O). Overall, waves induce dynamic cell shape changes via repetitive cycles of active cell elongation and active rounding, thereby driving cell body propulsion and swimming (Fig. 7N).

What is the function of fat body migration during metamorphosis? Blocking FBC migration through the various genetic manipulations used in our study resulted in a strong reduction in speed. Yet, this did not result in lethality, since animals survived into adulthood, suggesting that FBC migration is not essential for pupal development. However, we would expect migration defects to result in defects in recruitment of FBCs to wounds, as we have shown previously for expression of DN Zip (UAS-DN-Zip-YFP with an Lpp-Gal4 driver; Franz et al., 2018).

The generation of periodic contractile waves we find in swimming FBCs differs from *in vitro* swimming of leukocytes, which is driven by a constant retrograde actin flow that is regulated mainly by RhoA rather than Rac1 and Cdc42 (O'Neill et al., 2018; Aoun et al., 2020). Interestingly, we also see some evidence of a retrograde actin flow within the waves when using super-resolution microscopy. This raises several questions regarding how the dynamic actin network is organized. How are actin polymerization, actin branching, actin depolymerization and F-actin turnover regulated locally to produce travelling waves? Does self-organization via ‘advected percolation’ play a role in here, as has recently been described for blebbing amoeboid migration (Garcia-Arcos et al., 2024b)? What determines the site of actomyosin contraction and hence direction of migration? What is the crosstalk between the Rho GTPases to regulate actin wave formation and contraction? These will be interesting areas for future studies.

Confinement is usually seen as a requirement to allow cell propulsion in the absence of cell–substrate adhesion in amoeboid migration. This is because strong cell confinement induces continuous actin flows to drive migration by causing an increase in myosin II activity (Ruprecht et al., 2015; Liu et al., 2015; Logue et al., 2015). In contrast, FBCs swimming *in vivo* do not appear to be under any such strong confinement. Even in the more crowded areas such as the dorsal head and thorax, we rarely observe any flattened cell surface areas indicative of the cell being compressed. Moreover, FBCs migrate with similar high speed and periodic actin waves in the much less crowded ventral thorax where the swimming FBCs are rarely in contact with a solid substrate (Franz et al., 2018). However, we cannot rule out that sometimes a more transient, local confinement might contribute to FBC swimming migration in the crowded areas. Instead of confinement, the viscosity of the hemolymph might dictate the biophysical properties of the environment and hence enable FBC migration. Interestingly, the lack of strong confinement in the pupa could explain why migratory FBCs produce retrograde cortical actin waves rather than a continuous actin flow. This also highlights that the environment is a key factor in dictating migration modes and that it is important to study cell migration under physiological conditions.

Most types of cell migration are driven by cell protrusions at the front. FBCs appear to migrate without any protrusions at the cell front such as blebs, lamellipodia or filopodia. Instead, their cell front maintains a round shape throughout migration. This is somewhat similar to embryonic progenitor cells in zebrafish, which use amoeboid stable-bleb cell migration (Ruprecht et al., 2015), although these cells maintain a stable pear-like shape throughout their migration, whereas FBCs undergo dynamic cell deformations. We do not consider the round cell front of FBCs during wave formation to be a giant bleb for several reasons. First, the cortical network of actin bundles in FBCs rarely ever covers the whole cell surface, not even before wave formation. Second, although the round front of FBCs usually lacks cortical actin in contrast to the rear during wave formation, the front area does not usually recover its cortical actin following a wave. That said, we do sometimes observe that as an actin wave contracts at the rear, a large cell area near the rear that lacks visible cortical actin bulges outwards and then often retracts through a consequent actin wave. These bulges share some resemblance with large blebs; however, they appear to drive the retraction of cell areas at the rear, which results in cell rounding. In contrast, in classic blebbing migration, blebs normally drive expansion at the front.

A surprising finding from our *in vivo* study is that FBCs display a high level of heterogeneity of cellular behaviour as they navigate the complex environment inside the pupal body. Conversely, during mesenchymal migration, cells display a very stereotypical cellular behaviour of cycles of: (1) lamellipodium formation and new adhesion at the front, (2) resolution of adhesion at the rear and (3) rear retraction. Throughout long periods, these cells maintain a clear front–rear polarity, which is constantly reinforced through cell–substrate adhesion. In contrast, swimming FBCs migrate by random walk. This is potentially due to a lack of cell–substrate adhesion that could reinforce polarity. Maybe because of this, FBCs show a strikingly stochastic cell behaviour. Consecutive actin waves can form repetitively on the same side or instead appear in seemingly random locations. Waves can either lead to cell elongation or to rounding. Consequently, FBCs display a great variety of cell shapes, from spheres to oddly deformed prolate spheroids.

This poses the question of how such a stochastic behaviour can lead to the efficient long-range migration achieved by these cells? Interestingly, it has been shown the actin pulses that drive apical constriction in the *Drosophila* ventral furrow appear very stochastic because they come in three subtypes: ratcheted, unratcheted or unconstricting pulses (Xie and Martin, 2015). Similarly, it might be that there are different subtypes of actin waves during FBC swimming migration. Some might compress the rear of a round cell, which we speculate may result in hydrostatic pressure-driven cell elongation and cell propulsion. This elongated cell shape might then sometimes become stabilized, for example through remodelling of the cytoskeleton. This could then enhance the effect that the next wave can have on inducing a deformation, similar to the ratcheted actin pulses. In contrast, other types of waves might not actively drive cell propulsion but rather serve to speed up cell rounding by retracting the cell rear or bulged-out cell areas. In fact, active cell rounding might be equally important as active elongation, since a more spherical shape could allow the next wave to perform a more efficient power stroke. Altogether, this might be similar to rowers actively pulling the oars towards them for a power stroke as well as actively pushing the oars back to their original position to prepare for the next power stroke.

An intriguing question that arises from swimming migration is how the forces that are generated inside the cell get transmitted to the aqueous environment to propel the cell body forward. In mesenchymal migration this force transmission is accomplished by cell–extracellular matrix adhesion to allow traction. However, swimming cell migration does not appear to involve cell–extracellular matrix adhesion. Two models of force transmission in swimming migration have been proposed: non-reciprocal cell shape changes and cell membrane treadmilling (Paluch et al., 2016). *In vitro* studies of swimming migration of lymphocytes and macrophages have shown that the cortical actin flow is coupled to a rearward flow of plasma membrane proteins (membrane treadmilling), which is critical for swimming migration (O'Neill et al., 2018; Aoun et al., 2020). However, these studies have not found evidence for non-reciprocal cell shape changes being important, since cell deformations appear to play only a minor role. In contrast, here we show that dynamic cell deformations driven by actin waves are crucial for *in vivo* swimming migration of FBCs. Indeed, we find a positive correlation between absolute aspect ratio changes (increases and decreases in aspect ratio) and speed. Interestingly, our data suggest that contractile actin waves are not only responsible for driving cell elongation but are also important for actively driving subsequent cell rounding rather than this happening passively. Overall, our results provide some evidence in support of the model of non-reciprocal cell shape changes driving force transmission in *in vivo* swimming migration. Whether cell membrane treadmilling also plays a role in force transmission here will be the focus of future studies. Finally, another mechanism that could theoretically contribute to force transmission during *in vivo* swimming is ‘chimneying’, which has been described for leukocytes when confined between a slide and a coverslip as they push against the opposing surfaces (Malawista and de Boisfleury Chevance, 1997). While we have never observed strongly confined FBCs sandwiched between two substrates, we do find that FBCs in crowded areas frequently make transient contacts with other cells, although mostly with other swimming FBCs. Hence, it is unlikely that chimneying induced by transient confinement through bilateral cell–cell contacts significantly contributes to force transmission.

Altogether, here we describe for the first time an *in vivo* swimming cell migration mode that allows the fast travel of FBCs within the aqueous environment of the pupa over long distances. We propose that the stochastic formation of contractile actin waves enables the random-walk behaviour of individual cells, which collectively allows effective patrolling of the pupal body. This raises the intriguing question of whether this swimming migration mode might enable other cells, not just in invertebrates but potentially also in vertebrates, to perform fast efficient spreading when encountering certain liquid-filled environments, such as confining conjunctive tissues, or liquid-rich infected areas, such as oedemas in chronic wounds. This might be particularly useful for cells that need to travel across different body parts, such as immune cells and cancer cells during cancer metastasis.

## MATERIALS AND METHODS

### Fly stocks and maintenance

*Drosophila melanogaster* stocks and crosses were maintained and performed on cornmeal molasses food (38.3 ml molasses, 5.7 g agar, 38.3 g maize flour, 15.8 g yeast, 1.2 g nipagin, 11.9 ml ethanol, 4 ml propionic acid adding water to make a 1 l batch) at 25°C. Stocks obtained from the Bloomington *Drosophila* Stock Center (BDSC; NIH P40OD018537) were used in this study. The following lines were used in this paper: w^67^ as a control, Lsp2-Gal4 (BDSC: 6357), UAS-NLS-mCherry (BSDC: 38424), UAS-LifeAct-GFP (Zanet et al., 2012), UAS-LifeAct-Scarlet (Serna-Morales et al., 2023), UAS-GMA-GFP (Dutta et al., 2002), UAS-Utrophin-GFP (Rauzi et al., 2010), UAS-Rho1-GFP (BSDC: 9393), Ubi>Anillin-RBD-GFP (Munjal et al., 2015), sqh^AX3^;sqh>Sqh-GFP (BSDC: 57144), sqh>Rok^K116A^-Venus (Bulgakova et al., 2013), Lpp-GFP [Vienna *Drosophila* Resource Center (VDRC): FTRG-318255], Ubi>CaaX-RFP [*Drosophila* Genomics Resource Center (DGRC): 109826], UAS-Zipper-DN-GFP (Monier et al., 2010), UAS-Rho1-N19 (BSDC: 7327), UAS-Rho1-RNAi (gift from Brian Stramer, Kings College London, London, UK), UAS-Rok-RNAi 1 (BSDC: 28797) and 2 (VDRC: KK-104675), UAS-Sqh-RNAi (VDRC: KK-109493), UAS-Sqh-AA (BSDC: 64114), UAS-Dia RNAi 1 (BSDC: 28541) and 2 (VDRC: KK-103914), UAS-Dia-ΔDAD-GFP (BSDC: 56752), UAS-CDC42-N17 (BSDC: 6288), UAS-CDC42-RNAi (BSDC: 29004), UAS-Rac1-N17 (BSDC: 6292), UAS-Rac1-RNAi (BSDC: 28985), UAS-WASp-RNAi 1 (BSDC: 25955) and 2 (VDRC: KK-109220), UAS-Arp2-RNAi 1 (VDRC: KK-101999) and 2 (VDRC: GD-29944) and UAS-Arp3-RNAi (VDRC: GD-35260). A list of genotypes used in the figures of this manuscript is provided in Table S1. We used FlyBase (https://flybase.org/; releases 2020_06–2024_02) to find information on phenotypes, function, stocks and gene expression.

### Microscopy

Animals were kept at 25°C. Pupae were marked at the white pre-pupa stage (0 h AFP) and dissected at 16 h APF by removing the pupal case (Weavers et al., 2018) and placed on a coverslip on their dorsal side or on their head for imaging.

Movies of whole pupae used to track FBC migration were collected on a Zeiss Cell discovery 7 widefield microscope with a 5× objective lens with a 0.5 optovar to give a 2.5× magnification for 3 h with a time interval of 1 min 30 s (Fig. 1A,B; Fig. S1; Movie 1) or 5 min (Figs 3A–D, 4A–G, 5A–E, 6A–H; Figs S2A–E and S5A–G; Movie 7). Movies to assess actin waves and cell aspect ratio (images from Figs 3G–I, 4J–O, 5F–L, 6I–M; Movies 9, 11, 12, 13 and 14) were acquired with a Nikon SoRa spinning-disk confocal microscope using a 40× silicone objective for 20 min with a time interval of 30 s with a 1× optovar. ‘Higher resolution’ movies (Fig. 3F; Figs S2F–H, S3; Movies 3, 5 and 8B) were acquired with the Nikon SoRa spinning disk confocal microscope with a 40× silicone objective using a magnification of 2.8. High-resolution movies (Figs 1E, 3E and 4F; Movies 2, 8A and 10A; and movies used for quantifications in Figs 2B and 7F,G, and Fig. S6B–E) were obtained using a Nikon AX-R NSPARC microscope with a Plan Apo Lambda S 40×1.25 NA silicone objective in confocal mode, employing the resonant scanner with 4-line averaging. The movies were deconvolved using the Nikon NIS-Elements imaging software with a Richardson–Lucy deconvolution method, applying 9–13 iterations.

Super-resolution movies (Fig. 2C–D″ and Movie 6) were acquired using a Nikon AX-R NSPARC microscope with a Plan Apo Lambda S 40×1.25 NA silicone objective in super-resolution mode. The resonant unidirectional scanner was used with four integrations in the Nikon Spatial Array Confocal (NSPARC) detector, achieving an image calibration of 0.07 µm/pixel. The movies were deconvolved using the Nikon NIS-Elements imaging software with a Blind deconvolution method, applying nine iterations. Movie 10B was acquired with a Zeiss LSM 980 confocal microscope using a 63× oil objective.

Methylene Blue-stained (Sigma 115943), semi-thick serial transverse sections of 1 µm thickness (Fig. 1F), were prepared as described previously (Franz et al., 2018), and a Leica MICA with a 20× objective was used to collect images.

Images and movies of confocal movies were generated with FIJI ImageJ (Schindelin et al., 2012) to create *Z* projections, *Z* sections and orthogonal view images. We used the same brightness and contrast adjustment for control and experimental conditions. Movies and images were organized and annotated with VSDC Video Editor (https://www.videosoftdev.com/) and Adobe Illustrator.

### Analysis

#### Migration tracking for speed and straightness measurements

Nuclear tracking of FBCs was performed automatically in an unsupervised manner using IMARIS software (Oxford Instruments) in the dorsal head, thorax and abdomen of pupae. The tracking was obtained in 2D using *Z* projections of the 3D movies to avoid artefacts created in the *Z* axis due to poorer *Z* resolution. Tracking was done every 1 min 30 s (Fig. 1A,B; Fig. S1; Movie 1) or every 5 min (Figs 3A–D, 4A–G, 5A–E, 6A–H; Figs S2A–E and S5A–I; Movie 7). Only tracks with a duration of 1.5–3 h were selected and analysed (∼100 per pupa). We then examined visually to see whether the tracks were correct. Incorrect tracks were either corrected or deleted. Average speed or straightness per pupa were obtained by averaging the mean speed or straightness of all individual tracks (mean over 1.5–3 h depending on track length). The straightness was calculated by dividing total track displacement (shortest path from the first time point to the last time point) by actual track length (total track length from the first to the last point). Tracks were colour-coded based on their average speed or duration or speed over time in IMARIS. Tracks are shown in images as whole tracks and in movies as Dragon Tail tracks.

#### Actin wave angle analysis

In FIJI ImageJ, we quantified the angle between the line connecting the centre of the actin wave (visually detected) at peak time (time *n*) with the cell centroid and the front–rear axis of the cell with respect to the migration direction (line crossing cell centroids at time *n* and time *n*+2 min).

#### Actin wave number and FBC deformation analysis

Confocal movies were analysed in FIJI ImageJ. Due to technical limitations caused by light scattering, we imaged only the top of half FBCs. Hence, all our quantification were done in 2D using maximum *Z* projections of the confocal movies.

We quantified the number of actin waves and cell aspect ratio only in FBCs that were fully in the field of view throughout the 20–60 min-long movies and that migrated mainly in the *XY* axis horizontally and excluded cells moving within the *Z* axis diagonally or up or down. Visual assessment of actin wave numbers was performed by researchers unaware of the sample identity (by masking the details of genotype for FBCs in the *Z* projections before analysis). For this, cells in *Z* projections were followed throughout the length of the movie, and actin waves appearing anywhere across the whole cell were counted, not distinguishing between travelling waves and local pulses but by counting all events of a local cortical increase in Lifeact-GFP intensity followed by a decrease.

For ‘mean aspect ratio’ and ‘mean absolute aspect ratio change’ measurements, we manually drew the outline around a particular FBC every minute throughout the length of the movie and measured the aspect ratio in FIJI ImageJ. Next, we calculated the mean aspect ratio over 20 min. Mean aspect ratio changes (as a parameter reflecting cell shape changes and cell deformations) were assessed by quantifying the absolute change in aspect ratio for each timepoint of a movie compared to the previous time point (absolute value of aspect ratio of time point *n*+1 min minus aspect ratio of time point *n*) and determining the mean over 20 min. All these quantifications were performed by researchers unaware of the sample identity. Using the same cell outlines, we also obtained speed measurements in FIJI ImageJ using centroid displacement from one time point to the next (1 min later) and used these to calculate mean speed over 20 min. To synchronize waves for the measurements in Fig. 7F and Fig. S6C, we set the time of actin wave peaks (visually assessed) to 0 min and compared aspect ratio measurements from 5 min before the peak (−5 min) to 5 min after the peak (+5 min).

For quantification of mean fluorescence intensity and aspect ratio (Fig. 7E and Fig. S6A), the mean fluorescence intensity of Lifeact-GFP and the cell aspect ratio were measured in FIJI ImageJ every 30 s in the outlined cells.

#### Quantification of contact sites of FBCs with other tissues

We quantified the length of the FBC border in contact with the hemolymph, other FBCs, epidermis, hemocytes and other pupal tissues using FIJI ImageJ. We analysed only FBCs that were fully contained within the 15–20 serial sections analysed. The percentage of FBC surface contacting other tissues was calculated for each section by dividing the length of the FBC border in contact with another tissue by the total length of the FBC outline, and the mean was then calculated from all sections for each cell.

#### Statistics

Statistics were performed using GraphPad Prism 10. Gaussian distribution was assessed to test normality of datasets. We used parametric or non-parametric tests depending on the Gaussian or non-Gaussian characteristics of the data distribution. *P*<0.05 was set as the significance threshold. For the box and whisker plots, the median is plotted as a line inside the box. The box extends from the 25th to the 75th percentile, and the whole dataset is shown by the whiskers and dots. In scatter dot plots, the line in the middle indicates the median and the error bars show the extents of the whole dataset.

## Supplementary Material



10.1242/joces.263787_sup1 Supplementary information
