# Supplementary material for: Cell deformations generated by stochastic actomyosin waves drive in vivo random-walk swimming migration
Source: J Cell Sci. 2025 May 21;138(10):jcs263787. doi: 10.1242/jcs.263787 (PMC12148041; doi:10.1242/jcs.263787)
Supplement: Supplementary information [file joces-138-263787-s1.pdf]

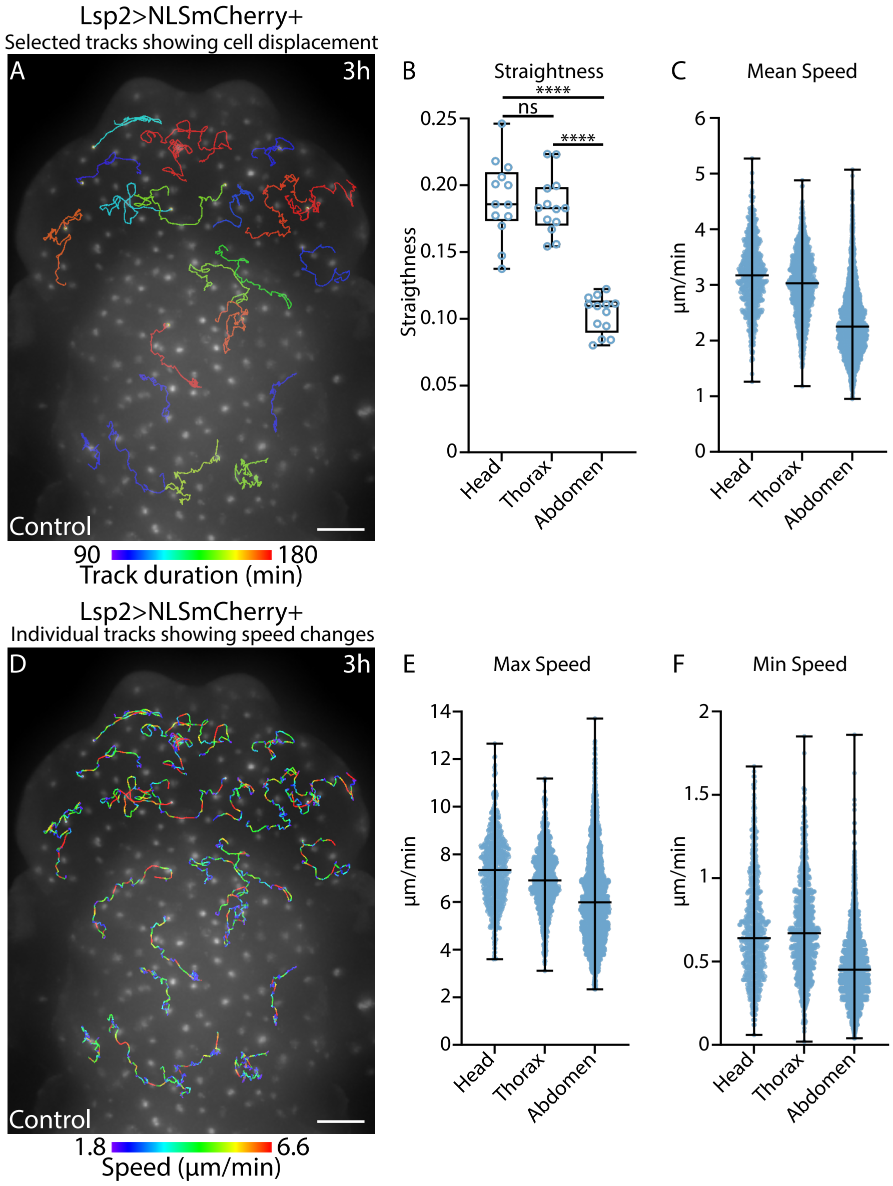

Fig. S1. Fat body cell speed varies during migration

(A) Widefield timelapse images of the dorsal head and thorax of *Drosophila* pupae expressing Lsp2-Gal4+UAS-NLS-mCherry+control from Figure 1A and Movie 1A showing only a selection of the 1h30-3h tracks (shown color-coded according to track duration) to visualise the total distance that individual FBCs travel inside the pupa.

(B) Quantification of mean straightness of the tracks of migrating FBC expressing Lsp2-Gal4+UAS-NLS-mCherry+control in the head, thorax and abdomen (n:13pupae). Showing mean straightness for each pupa calculated from the mean straightness of each of its 1h30-3h-long tracks. Ordinary one-way ANOVA test with multiple comparisons, ns p=0.9302 and **** p<0.0001.

Journal of Cell Science • Supplementary information

(C) Quantification of mean FBC speed in the head, thorax and abdomen of *Drosophila* pupae expressing Lsp2-Gal4+UAS-NLS-mCherry+control. Showing mean speed of all tracks of FBCs in the head (n:659cells), thorax (n:989cells) and abdomen (n:2366cells) from 13 pupae.

(D) Showing the same selection of 1h30-3hs tracks from (A) but color-coded by speed over time.

(E-F) Quantification of maximum speed (E) and minimum speed (F) from each FBC track in the head, thorax and abdomen of *Drosophila* pupae expressing Lsp2-Gal4+UAS-NLS-mCherry+control. Showing data from all tracks of FBCS in the head (n:659cells), thorax (n:989cells) and abdomen (n:2366cells) from 13 pupae.

Scale bars, 100 µm.

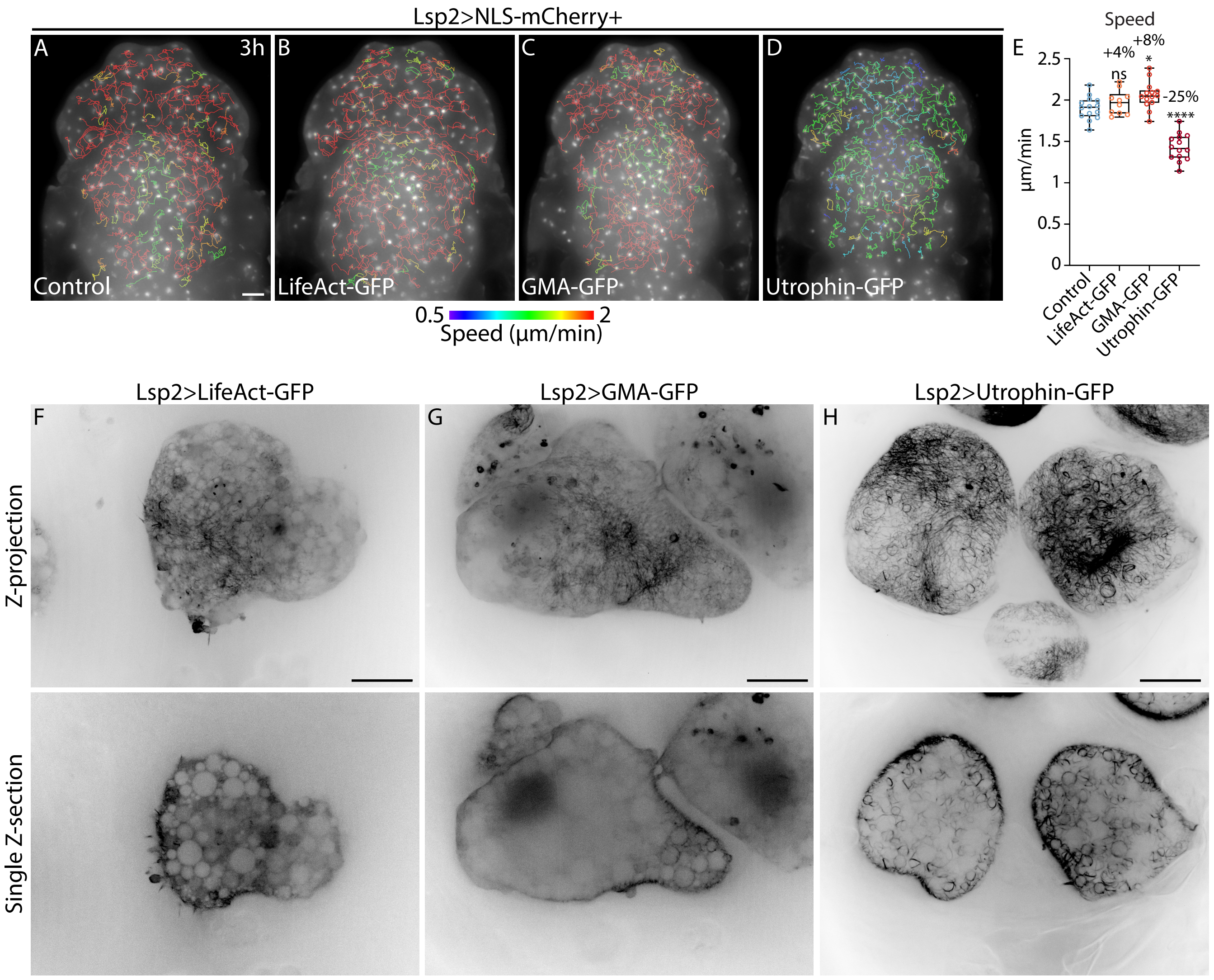

Fig. S2. LifeAct labels actin meshwork without affecting fat body cell migration.

(A-D) Widefield timelapse images of the dorsal head and thorax of *Drosophila* pupae expressing Lsp2-Gal4+UAS-NLS-mCherry+control (A), +UAS-LifeAct-GFP (B), +UAS-GMA-GFP (C) or +UAS-Utrophin-GFP (D). 1h30-3h-long migration tracks are shown color-coded according to their mean speed.

(E) Quantification of mean FBC speed from (A-D). Control (n:14pupae), LifeAct-GFP (n:10pupae), GMA-GFP (n:14pupae) and Utrophin-GFP (n:14pupae). Showing mean speed for each pupa calculated from the mean speed of each of its 1h30-3h-long tracks. One-way ANOVA test with multiple comparisons, ns p=0.591, *p=0.0293 and ****p<0.0001.

(F-H) Confocal time-lapse images of actin in FBCs expressing Lsp2-Gal4+UAS-LifeAct-GFP (F), +UAS-GMA-GFP (G) or +UAS-Utrophin-GFP (H). Z-projection in the top and Z-section in the bottom. See Movie 3.

Scale bars, 100 µm (A-D), 20 µm (F-H).

Journal of Cell Science • Supplementary information

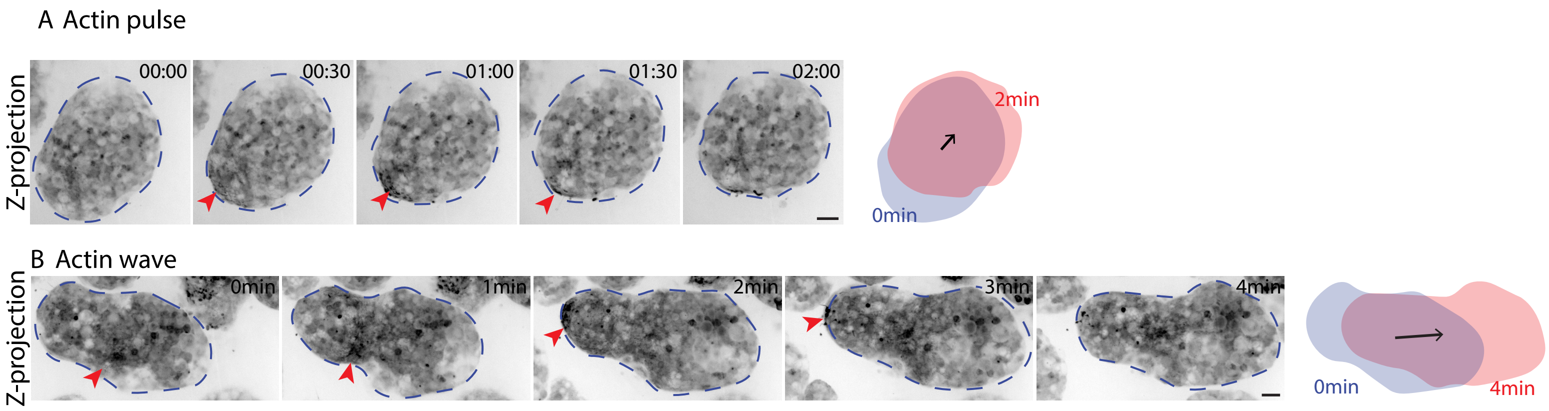

Fig. S3. Examples of actin pulse and actin wave.

(A) Confocal time-lapse images of Lsp2-Gal4+UAS-LifeAct-GFP-expressing FBCs. Actin pulse: red arrowheads; FBC: blue-dotted outline. See Movie 5A.

(B) Confocal time-lapse images of Lsp2-Gal4+UAS-LifeAct-GFP-expressing FBCs. Actin wave: red arrowheads; FBC: blue-dotted outline. See Movie 5B.

Scale bars, 10 µm

Journal of Cell Science • Supplementary information

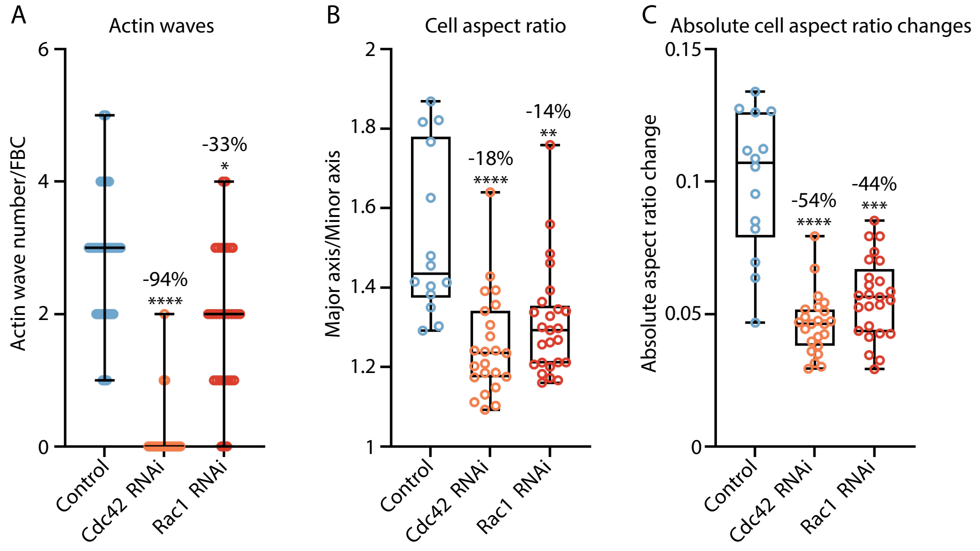

Fig. S4. Cdc42 and Rac1 RNAi lead to a reduction in actin wave numbers, aspect ratio and aspect ratio changes.

(A) Quantification of actin wave numbers from 20min-long movies in FBCs expressing Lsp2-Gal4+UAS-LifeAct-GFP +control (n:35cells/12pupae), +UAS-Cdc42 RNAi (n:23cells/5pupae) and +UAS-Rac1 RNAi (n:32cells/8pupae). Kruskal-Wallis test with multiple comparisons, ****$p<0.0001$ and *$p=0.0127$.

(B) Quantification of mean aspect ratio of FBCs expressing Lsp2-Gal4+UAS-LifeAct-GFP +control (n:14cells/4pupae), +UAS-Cdc42 RNAi (n:23cells/5pupae) and +UAS-Rac1 RNAi (n:25cells/6pupae). Kruskal-Wallis test with multiple comparisons, ****$p<0.0001$ and **$p=0.0022$.

(C) Quantification absolute aspect ratio change over time of FBCs expressing Lsp2-Gal4+UAS-LifeAct-GFP +control (n:14cells/4pupae), +UAS-Cdc42 RNAi (n:23cells/5pupae) and +UAS-Rac1 RNAi (n:25cells/6pupae). Kruskal-Wallis test with multiple comparisons, ****$p<0.0001$ and ***$p=0.0006$.

Journal of Cell Science • Supplementary information

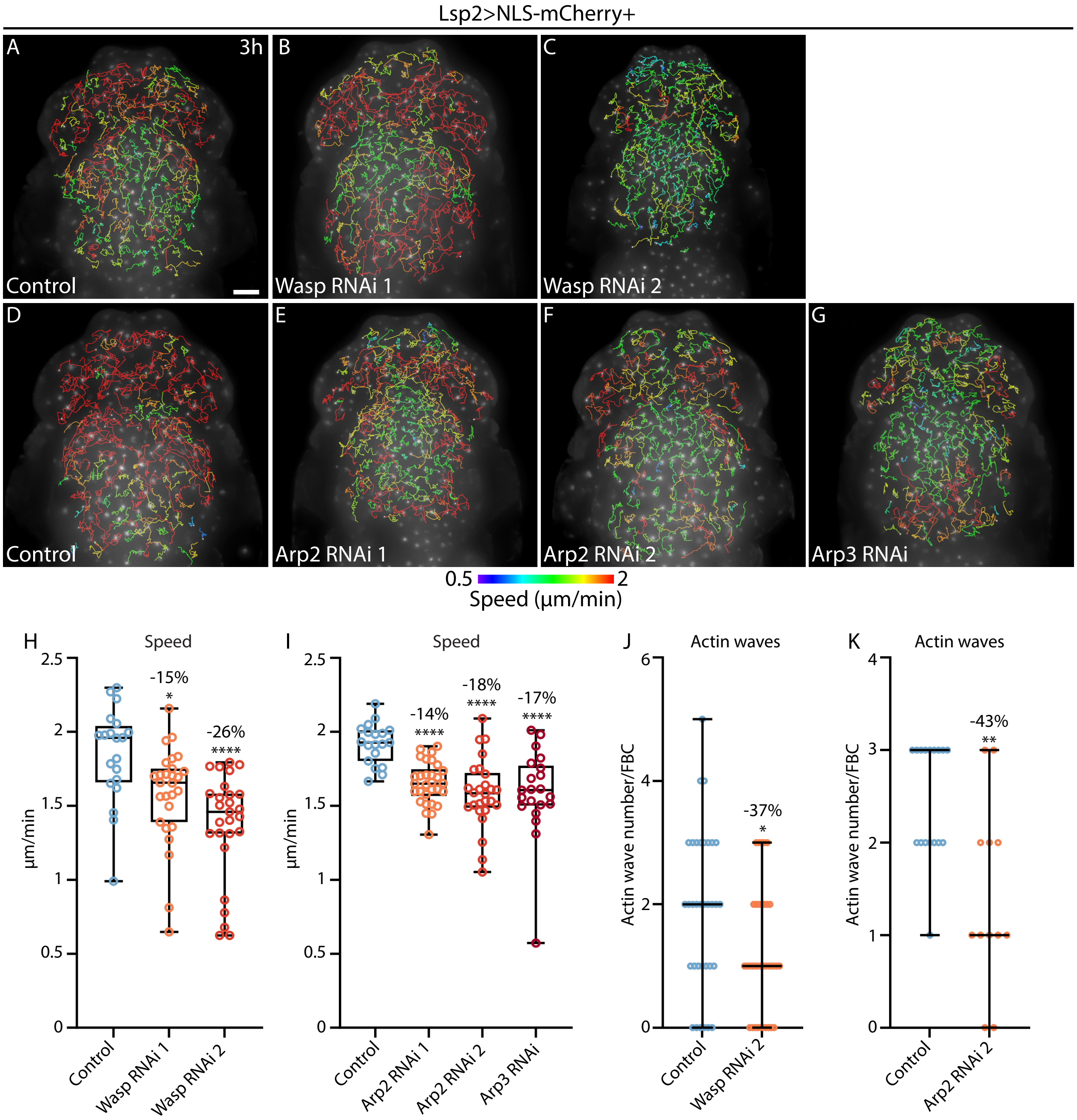

Fig. S5. Wasp and the Arp2/3 complex are involved in fat body cell migration and actin wave formation.

(A-G) Widefield timelapse images of the dorsal head and thorax of *Drosophila* pupae expressing Lsp2-Gal4+UAS-NLS-mCherry+control (A, D), +UAS-Wasp RNAi1 (B), +UAS-Wasp RNAi2 (C), +UAS-Arp2 RNAi1 (E), +UAS-Arp2 RNAi2 (F) and +UAS-Arp3 RNAi (G). 1h30-3h-long migration tracks are shown color-coded according to their mean speed.

(H, I) Quantification of mean FBC speed from (A-G). Control (n:20pupae), Wasp RNAi1 (n:27pupae) and Wasp RNAi2 (n:27pupae) (H) and control (n:19pupae), Arp2 RNAi1 (n:30pupae), Arp2 RNAi2 (n:26pupae) and Arp3 RNAi (n:21pupae) (I). Showing mean speed for each pupa calculated from the mean speed of each of its 1h30-3h-long tracks. Kruskal-Wallis test with multiple comparisons, *p = 0.0172 and ****p<0.0001.

(J, K) Quantification of actin wave numbers from 20min-long movies in FBCs expressing Lsp2-Gal4+UAS-LifeAct-GFP+control (n:34cells/3pupae (J) and n:17cells/5pupae (K)), +UAS-Wasp RNAi 2 (n:36cells/4pupae) (J) and +UAS-Arp2 RNAi 2 (n:12cells/4pupae) (K). Mann-Whitney test, *p=0.0219 (J) and **p=0.0035 (K).

Scale bar, 100 µm.

Journal of Cell Science • Supplementary information

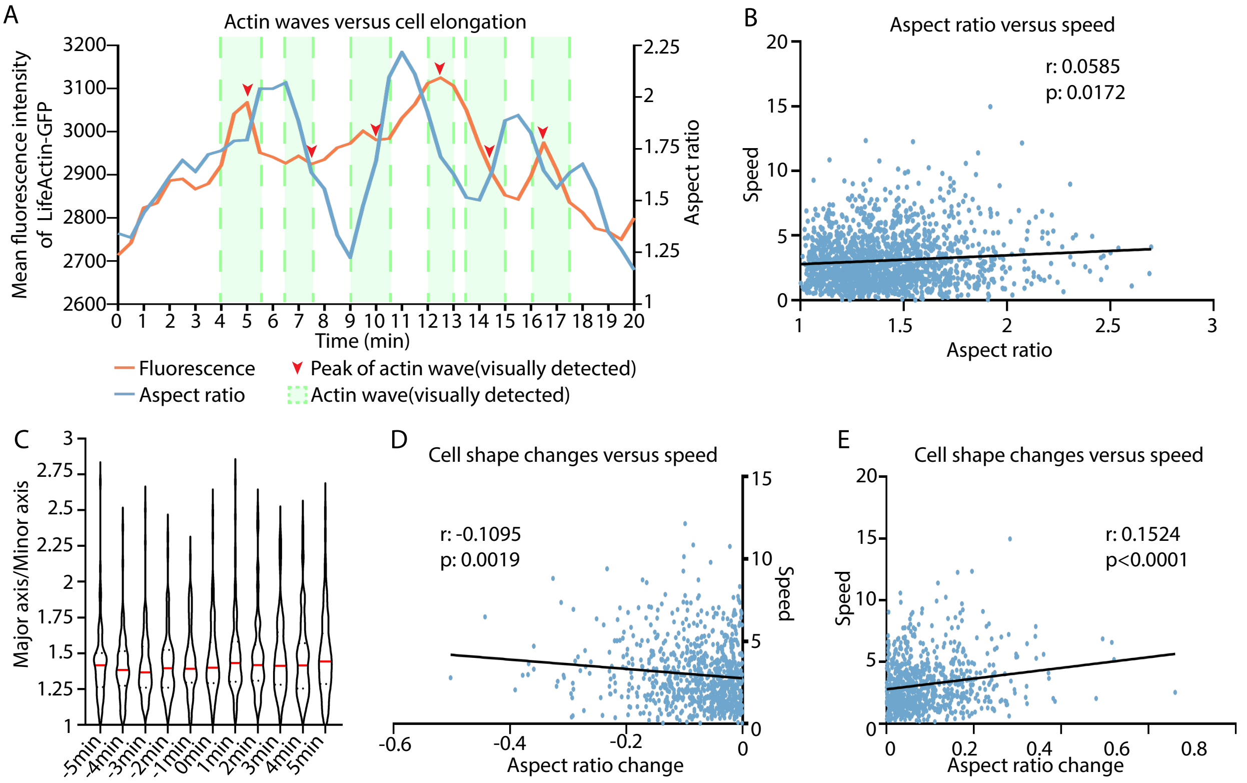

Fig. S6. Cell deformations are a major contributor to fat body cell migration.

(A) Quantification of mean fluorescence intensity of LifeAct-GFP and cell aspect ratio of FBC from movie 4 of Lsp2-Gal4+UAS-LifeAct-GFP-expressing FBC. Visually detected actin waves and peaks: green areas and red arrowheads, respectively. See Movie 4.

(B) Aspect ratio as a function of speed of Lsp2-Gal4+UAS-LifeAct-GFP-expressing FBCs of all timepoints from confocal time-lapse movies (n:1654). Spearman test.

(C) Aspect ratio of Lsp2-Gal4+UAS-LifeAct-GFP-expressing FBCs from 5 minutes before to 5 minutes after the visually detected peak of actin wave (n:83 waves).

(D) Negative aspect ratio changes as function of speed of Lsp2-Gal4+UAS-LifeAct-GFP-expressing FBCs of all timepoints from confocal time-lapses (n:804). Spearman test.

(E) Positive aspect ratio changes as function of speed of Lsp2-Gal4+UAS-LifeAct-GFP-expressing FBCs of all timepoints from confocal time-lapses (n:842). Spearman test.

Table S1. Genotypes used in figures Table listing the genotypes used in the various figures.

Figure	Genotype
1A	;; Lsp2-Gal4+UAS-NLS-mCherry/+
1B	; UAS-Zipper-DN-GFP/+ ; Lsp2-Gal4+UAS-NLS-mCherry/+
1C	;; Lpp-GFP+Ubi>CAAX-RFP/Lsp2-Gal4+UAS-NLS-mCherry
2	;; Lsp2-Gal4+UAS-LifeAct-GFP+UAS-NLS-mCherry/+
3A	;; Lsp2-Gal4+UAS-NLS-mCherry/+
3B	UAS-Rho1-N19/+ or Y ;; Lsp2-Gal4+UAS-NLS-mCherry/+
3C	;; Lsp2-Gal4+UAS-NLS-mCherry/UAS-Rho1 RNAi
3E	; UAS-Rho1-GFP/+ ; Lsp2-Gal4+UAS-NLS-mCherry/+
3F	; Ubi>Ani-RBD-GFP/Ubi>Ani-RBD-GFP ;
3G	;; Lsp2-Gal4+UAS-LifeAct-GFP+UAS-NLS-mCherry/+
3I	UAS-Rho1-N19/+ ;; Lsp2-Gal4+UAS-LifeAct-GFP+UAS-NLS-mCherry/+
4A	;; Lsp2-Gal4+UAS-NLS-mCherry/+
4B	;; Lsp2-Gal4+UAS-NLS-mCherry/UAS-Rok RNAi 1
4C	; UAS-Rok RNAi 2/+ ; Lsp2-Gal4+UAS-NLS-mCherry/+
4D	;; Lsp2-Gal4+UAS-NLS-mCherry/UAS-Sqh-AA
4E	; UAS-Sqh RNAi ; Lsp2-Gal4+UAS-NLS-mCherry/+
4F	; UAS-Zipper-DN-GFP/+ ; Lsp2-Gal4+UAS-NLS-mCherry/+
4H	;; Sqh>Rok^{K116A}-Venus/ Sqh>Rok^{K116A}-Venus
4I	Sqh^{Ax3};;Sqh>Sqh-GFP/Lsp2-Gal4+UAS-NLS-mCherry
4J	;; Lsp2-Gal4+UAS-LifeAct-GFP+UAS-NLS-mCherry/+
4L	;; Lsp2-Gal4+UAS-LifeAct-GFP+UAS-NLS-mCherry/UAS-Rok RNAi 1
4N	;; Lsp2-Gal4+UAS-LifeAct-GFP+UAS-NLS-mCherry/UAS-Sqh-AA
5A	;; Lsp2-Gal4+UAS-NLS-mCherry/+
5B	;; Lsp2-Gal4+UAS-NLS-mCherry/UAS-Dia RNAi 1
5C	; UAS-Dia RNAi 2/+ ; Lsp2-Gal4+UAS-NLS-mCherry/+
5D	;; Lsp2-Gal4+UAS-NLS-mCherry/UAS-DiaΔDAD-GFP
5F	;; Lsp2-Gal4+UAS-LifeAct-GFP+UAS-NLS-mCherry/+
5H	; UAS-Dia RNAi 2/+ ; Lsp2-Gal4+UAS-LifeAct-GFP+UAS-NLS-mCherry/+
5J	;; Lsp2-Gal4+UAS-LifeAct-Scarlet /+
5L	;; Lsp2-Gal4+UAS-LifeAct-Scarlet/UAS-DiaΔDAD-GFP
6A	;; Lsp2-Gal4+UAS-NLS-mCherry/+
6B	; UAS-Cdc42-N17/+ ; Lsp2-Gal4+UAS-NLS-mCherry/+
6C	UAS-Cdc42 RNAi/+ ;; Lsp2-Gal4+UAS-NLS-mCherry/+
6E	;; Lsp2-Gal4+UAS-NLS-mCherry/+
6F	;; Lsp2-Gal4+UAS-NLS-mCherry/UAS-Rac1-N17
6G	;; Lsp2-Gal4+UAS-NLS-mCherry/UAS-Rac1 RNAi
6I	;; Lsp2-Gal4+UAS-LifeAct-GFP+UAS-NLS-mCherry/+
6K	; UAS-CDC42-N17/+ ; Lsp2-Gal4+UAS-LifeAct-GFP+UAS-NLS-mCherry/+
6M	;; Lsp2-Gal4+UAS-LifeAct-GFP+UAS-NLS-mCherry/UAS-Rac1-N17
7	;; Lsp2-Gal4+UAS-LifeAct-GFP+UAS-NLS-mCherry/+

S1	*;; Lsp2-Gal4+UAS-NLS-mCherry/+*
S2A	*;; Lsp2-Gal4+UAS-NLS-mCherry/+*
S2B and F	*;; Lsp2-Gal4+UAS-NLS-mCherry/UAS-LifeAct-GFP*
S2C and G	*;; Lsp2-Gal4+UAS-NLS-mCherry/UAS-GMA-GFP*
S2D and H	*;; Lsp2-Gal4+UAS-NLS-mCherry/UAS-Utrophin-GFP*
S3	*;; Lsp2-Gal4+UAS-LifeAct-GFP+UAS-NLS-mCherry/+*
S5A	*;; Lsp2-Gal4+UAS-NLS-mCherry/+*
S5B	*;; Lsp2-Gal4+UAS-NLS-mCherry/UAS-Wasp RNAi 1*
S5C	*; UAS-Wasp RNAi 2 ; Lsp2-Gal4+UAS-NLS-mCherry/+*
S5D	*;; Lsp2-Gal4+UAS-NLS-mCherry/+*
S5E	*; UAS-Arp2 RNAi 1; Lsp2-Gal4+UAS-NLS-mCherry/+*
S5F	*;; Lsp2-Gal4+UAS-NLS-mCherry/ UAS-Arp2 RNAi 2*
S5G	*;; Lsp2-Gal4+UAS-NLS-mCherry/UAS-Arp3 RNAi*

Journal of Cell Science • Supplementary information

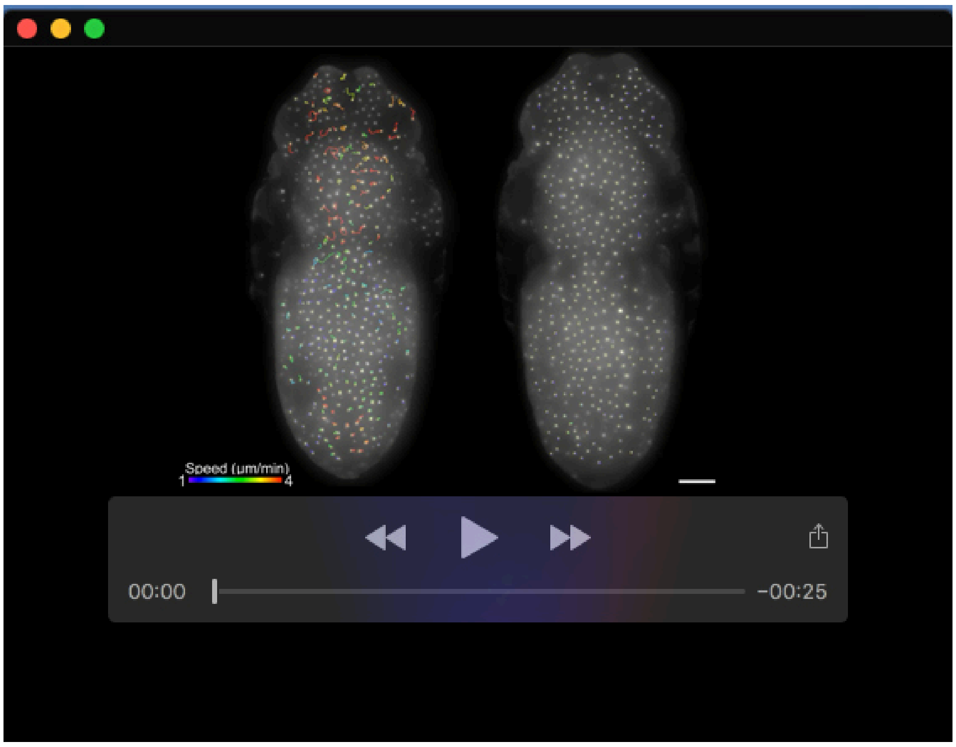

Movie 1. Fat body cells migrate in the whole pupa – Related to Figure 1A-B.

Widefield movies of the dorsal view of *Drosophila* pupae expressing Lsp2-Gal4+UAS-NLS-mCherry+control (A) or +UAS-Zipper-DN-GFP (B). Only continuous 1h30-3h long migration tracks with a dragon-tail are shown color-coded according to their mean speed. Elapsed time is in top left corner in minutes:seconds. Scale bar, 200 µm.

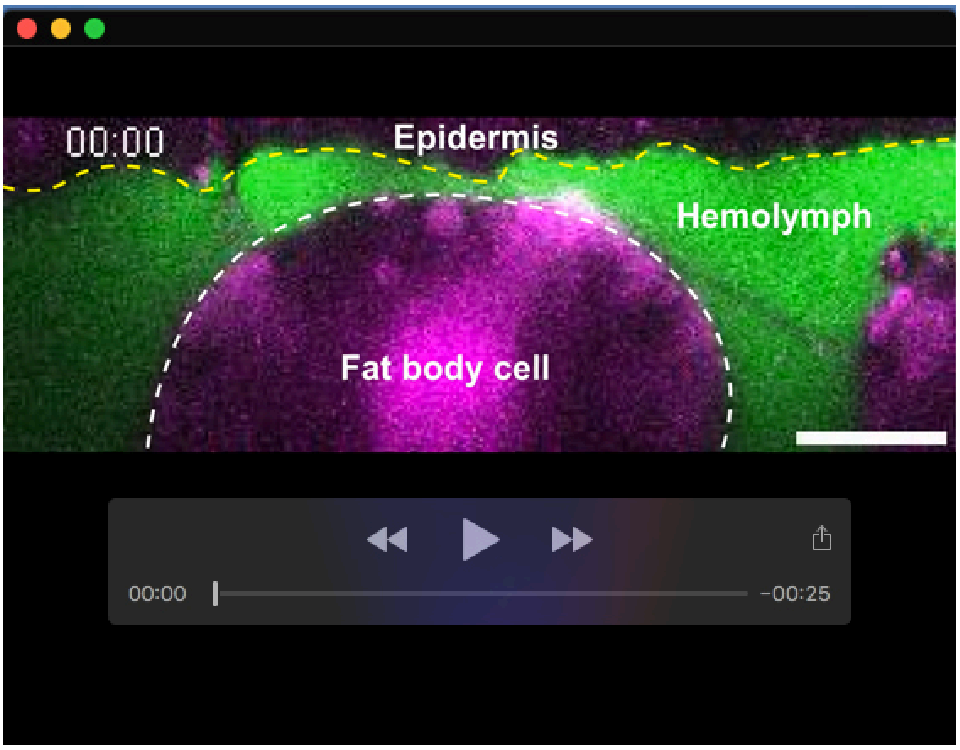

Movie 2. Fat body cells swim inside the hemolymph – Related to Fig. 1E.

Confocal movie of FBC swimming in the hemolymph near the epidermis in a Lpp-GFP+Ubi>CaaX-RFP+Lsp2-Gal4+UAS-NLS-mCherry pupa (hemolymph in green; epidermis in magenta on the top; FBCs in faint magenta with bright magenta nuclei). Elapsed time is in top left corner in minutes:seconds. Scale bar, 20 µm.

Journal of Cell Science • Supplementary information

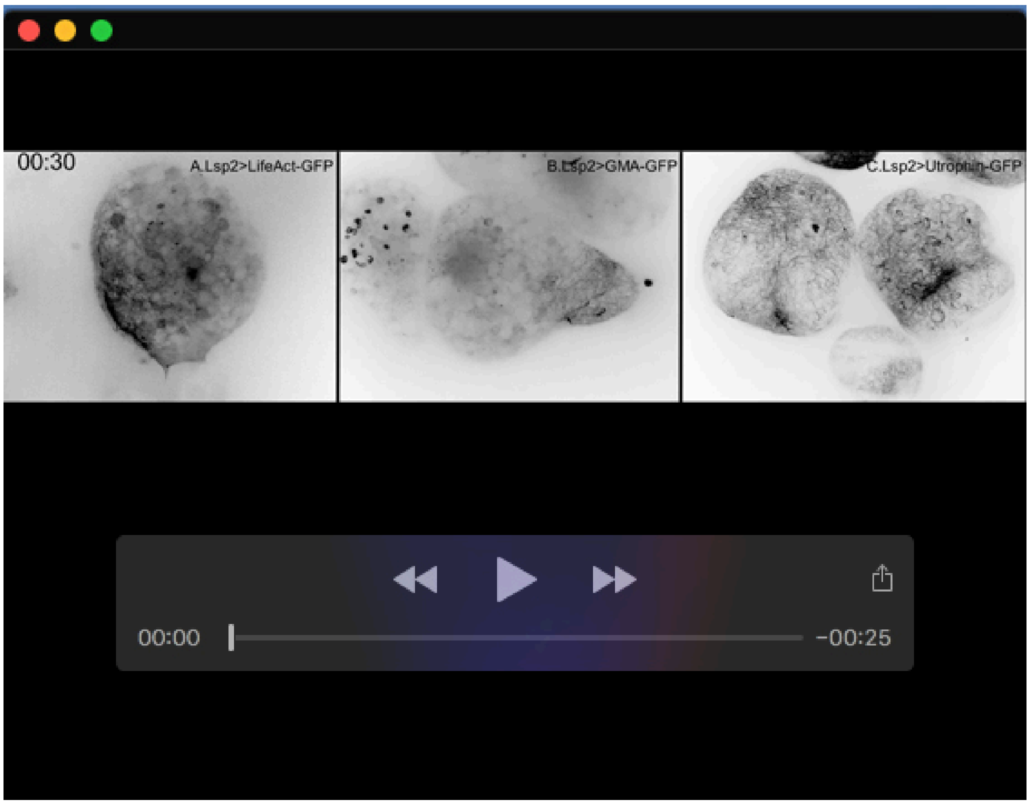

Movie 3. Actin markers – Related to Supplementary figure 2F-H.

Confocal movies of actin dynamics in migrating FBCs expressing Lsp2-Gal4+UAS-LifeAct-GFP (A), +UAS-GMA-GFP (B) and +UAS-Utrophin-GFP (C). Elapsed time shown in top left corner in minutes:seconds. Scale bar, 20 μm.

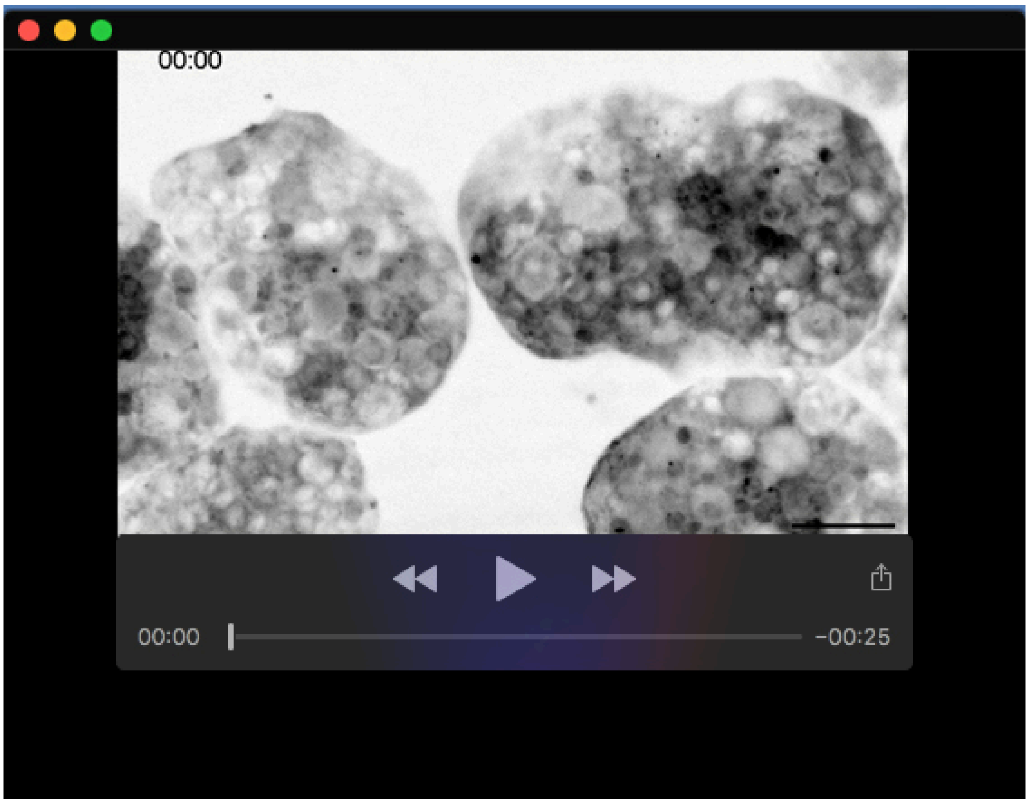

Movie 4. Periodic actin waves drive non-directed fat body cell swimming migration – Related to Fig. 2A.

Confocal movie of actin dynamics in migrating FBCs expressing Lsp2-Gal4+UAS-LifeAct-GFP. Red arrowheads point at consecutive actin waves in the rear of a migrating FBC. Elapsed time shown in top left corner in minutes:seconds. Scale bar, 20 μm.

Journal of Cell Science • Supplementary information

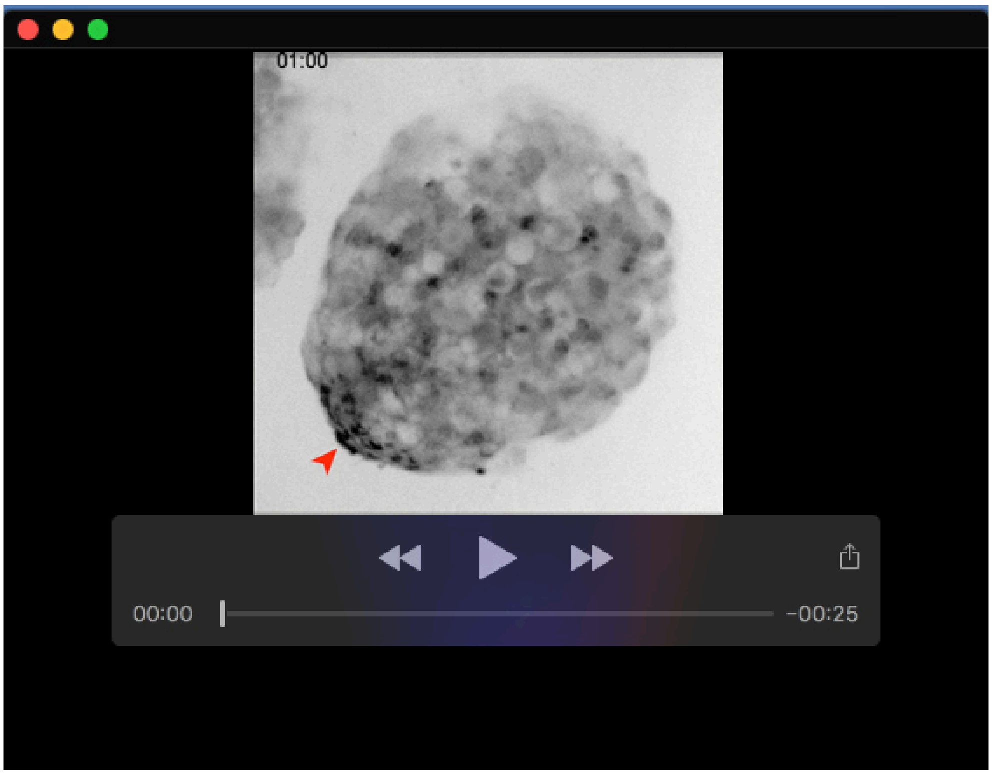

Movie 5. Examples of actin pulse and actin wave– Related to Supplementary Fig. 3.

Confocal movies of actin pulse (A) or wave (B) in migrating FBCs expressing Lsp2-Gal4 +UAS-LifeAct-GFP. Red arrowheads point at an actin pulse in the rear of a migrating FBC (A) or at an actin wave (B) travelling along a migratory FBC. Elapsed time shown in top left corner in minutes:seconds. Scale bar, 10 µm

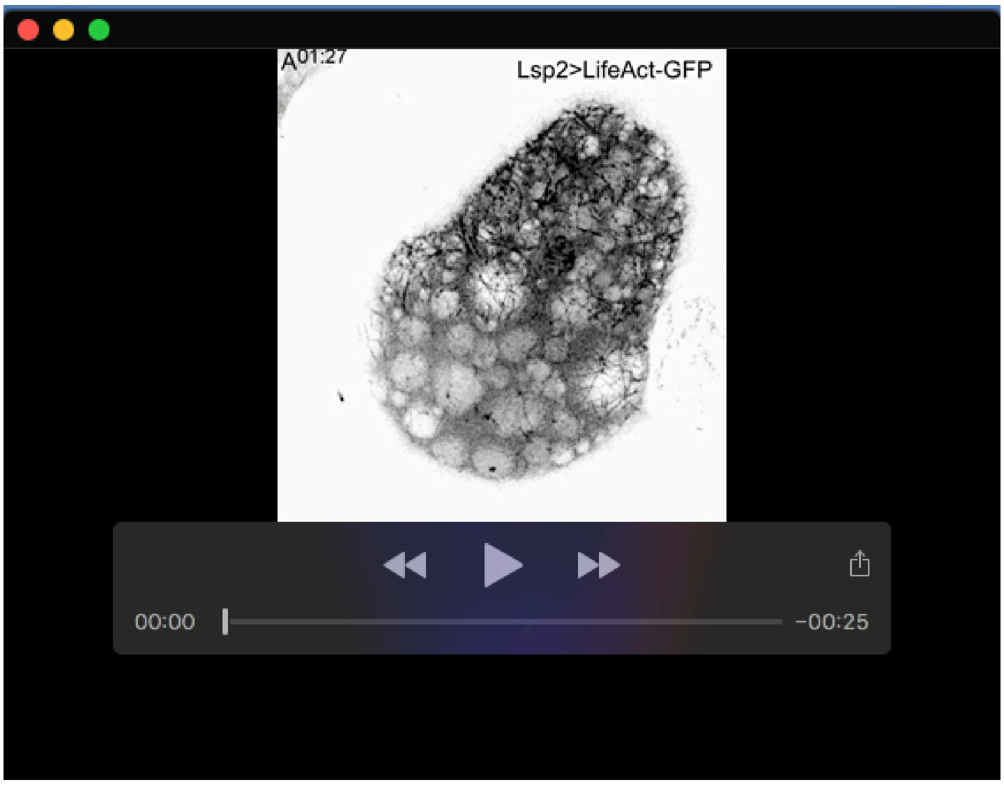

Movie 6. Super-resolution of actin wave – Related to Fig. 2C-D.

(A-B) Super-resolution confocal movies of a Lsp2-Gal4+UAS-LifeAct-GFP expressing FBC followed by magnified movies. Red arrowheads show actin meshwork and blue arrowheads show long actin bundles. Elapsed time shown in top left corner in minutes:seconds. Scale bar, 20 µm (full view), 5 µm (zoom).

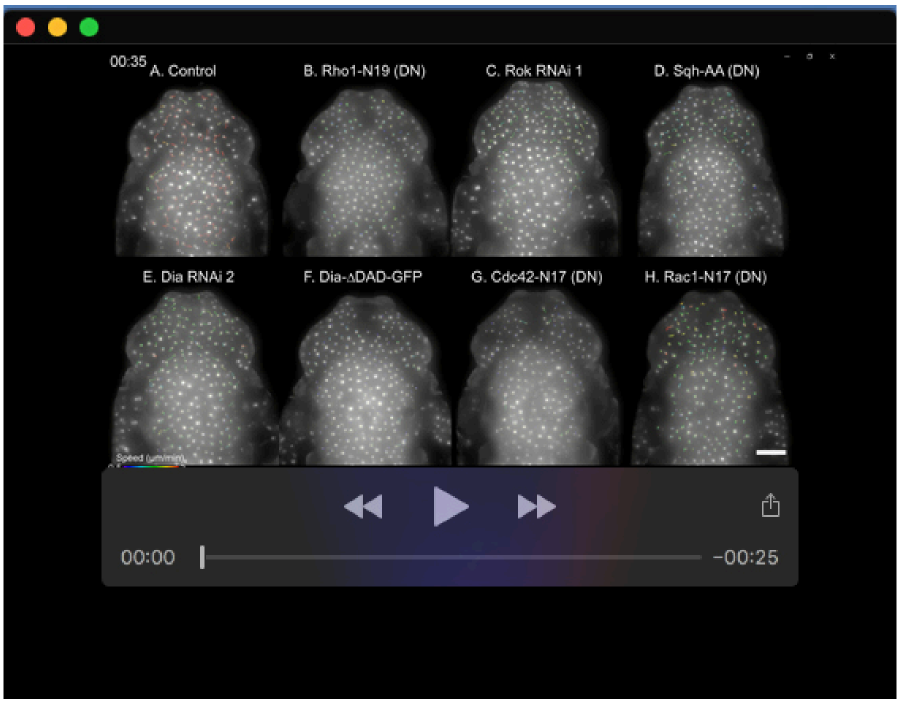

Movie 7. Tracking movies of pupae with genetic manipulations of actomyosin regulators – Related to Figs. 3A-B, 4B-D, 5C-D and 6B-F.

Widefield movies of the dorsal head and thorax of *Drosophila* pupae expressing Lsp2-Gal4 +UAS-NLS-mCherry+control (A), +UAS-Rho1-N19 (B), +UAS-Rok RNAi 1 (C), +UAS-Sqh-AA (DN) (D), +UAS-Dia RNAi 2 (E), +UAS-Dia-ΔDAD-GFP (CA) (F), +UAS-Cdc42-N17 (G) or +UAS-Rac1-N17 (DN) (H). Only continuous 1h30-3h long migration tracks with a dragon-tail are shown color-coded according to their mean speed. Elapsed time shown in top left corner in hours:minutes. Scale bar, 200 µm.

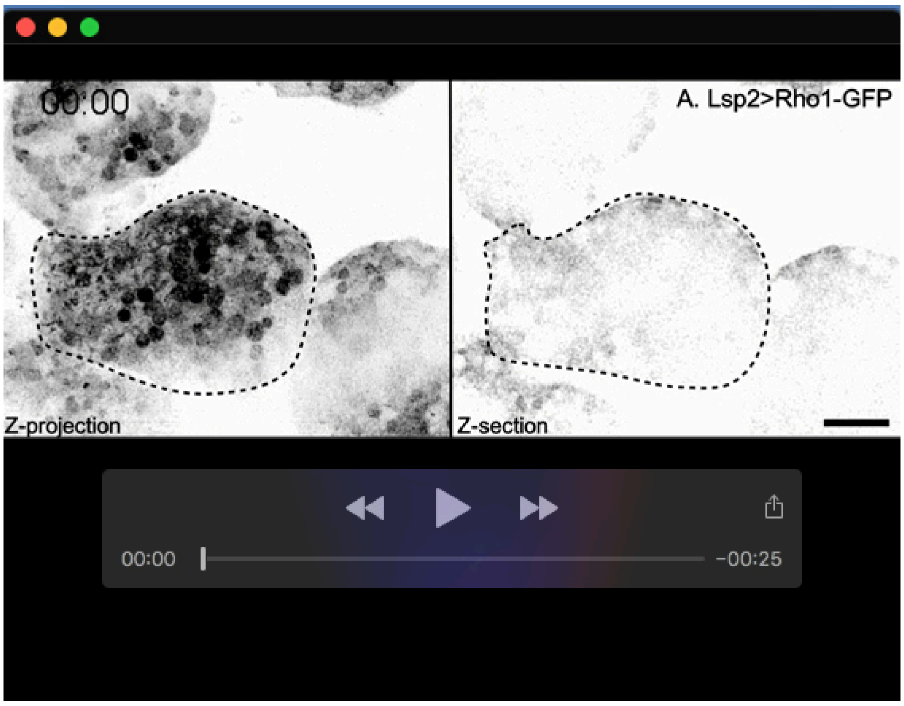

Movie 8. Rho1 accumulates in fat body cell rear during migration – Related to Fig. 3E-F.

Confocal movies of a swimming FBC expressing Lsp2-Gal4+UAS-Rho1-GFP (A) or Ani-RBD-GFP under the control of the ubiquitin p63E promoter (B). Red arrowhead points at the accumulation of Rho1-GFP or Ani-RBD-GFP as punctae in the rear of a migratory FBC. Yellow-dotted line outlines a hemocyte. Left panel is the maximum projection of the total Z-stack and right panel is one Z-section. Elapsed time shown in top left corner in minutes:seconds. Scale bar, 20 µm.

Journal of Cell Science • Supplementary information

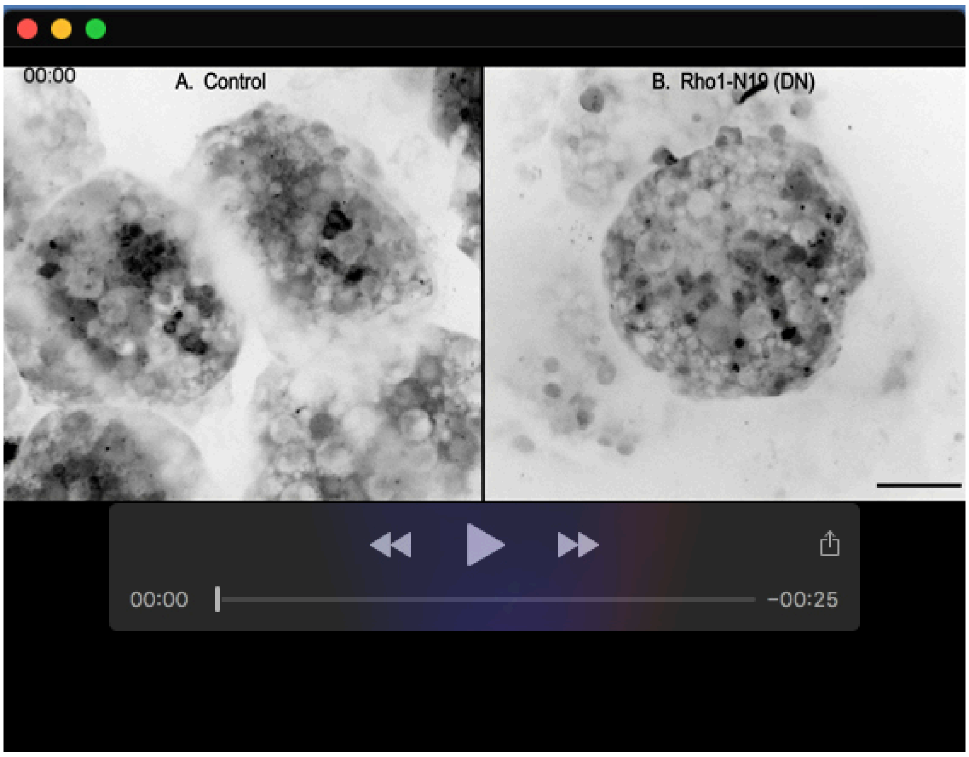

Movie 9. Rho1 is crucial for actin waves and fat body cell deformations – Related to Fig. 3G-J.

Confocal movies of actin dynamics and cell deformation from FBCs expressing Lsp2-Gal4+UAS-LifeAct-GFP+control (A) or +UAS-Rho1-N19 (B). Red arrowhead points at actin wave. Note that white line in (A) is the shadow formed by a cuticle fold. Elapsed time shown in top left corner in minutes:seconds. Scale bar, 20 µm.

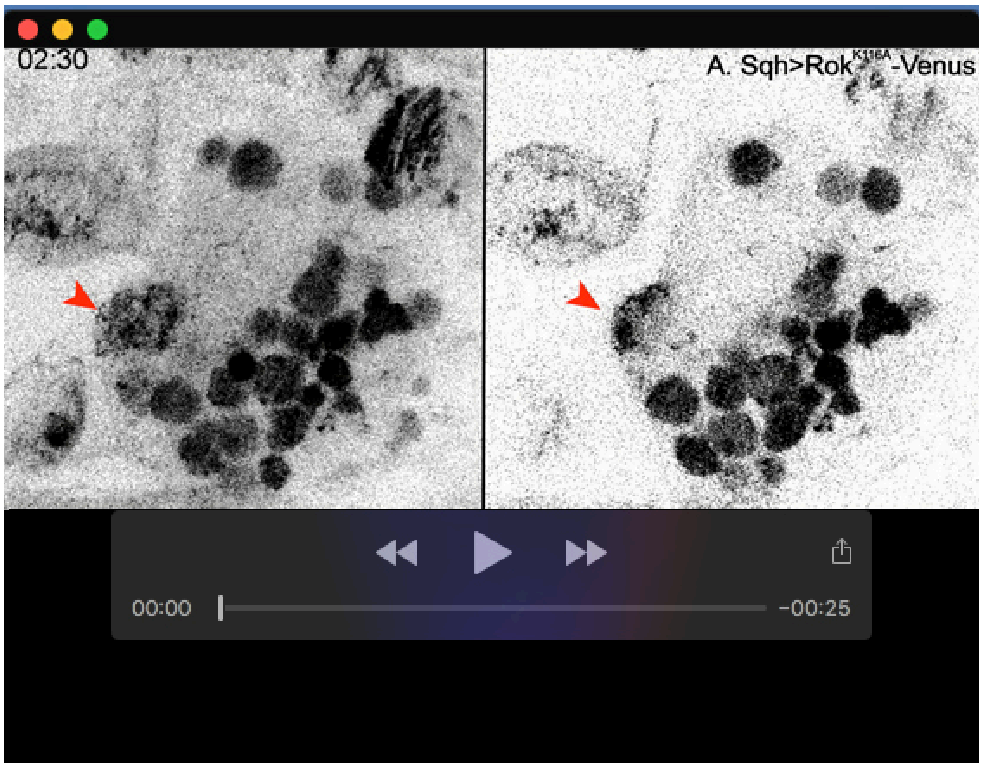

Movie 10. Rok and myosin II are found at the rear of a migrating fat body cell – Related to Fig. 4H-I.

Confocal movie of migrating FBCs expressing Rok^{K116A}-Venus under the sqh promoter (A) and Sqh-GFP under the sqh promoter in a sqh mutant background (B). Red arrowhead points the accumulation of Rok^{K116A}-Venus or Sqh-GFP in the rear of a migrating FBC. For Rok-Venus, left panel is the maximum projection of the total Z-stack and right panel is the maximum projection of 4 deeper Z-sections. Elapsed time shown in top left corner in minutes:seconds. Scale bar, 20 µm.

Journal of Cell Science • Supplementary information

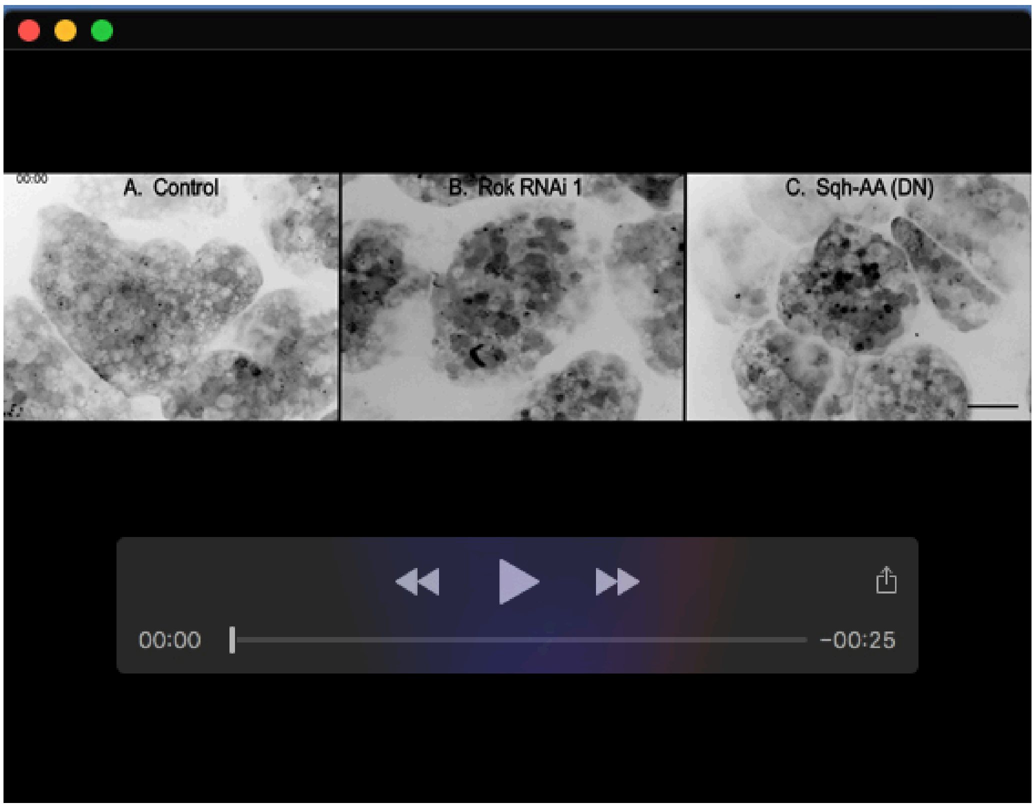

Movie 11. Rok and myosin II are needed for fat body cell deformations – Related to Fig. 4J-O.

Confocal movies of actin dynamics and cell deformation from FBCs expressing Lsp2-Gal4+UAS-LifeAct-GFP+control (A), +UAS-Rok RNAi1 (B) or +UAS-Sqh-AA (DN) (C). Red arrowhead points at actin wave. Elapsed time shown in top left corner in minutes:seconds. Scale bar, 20 µm.

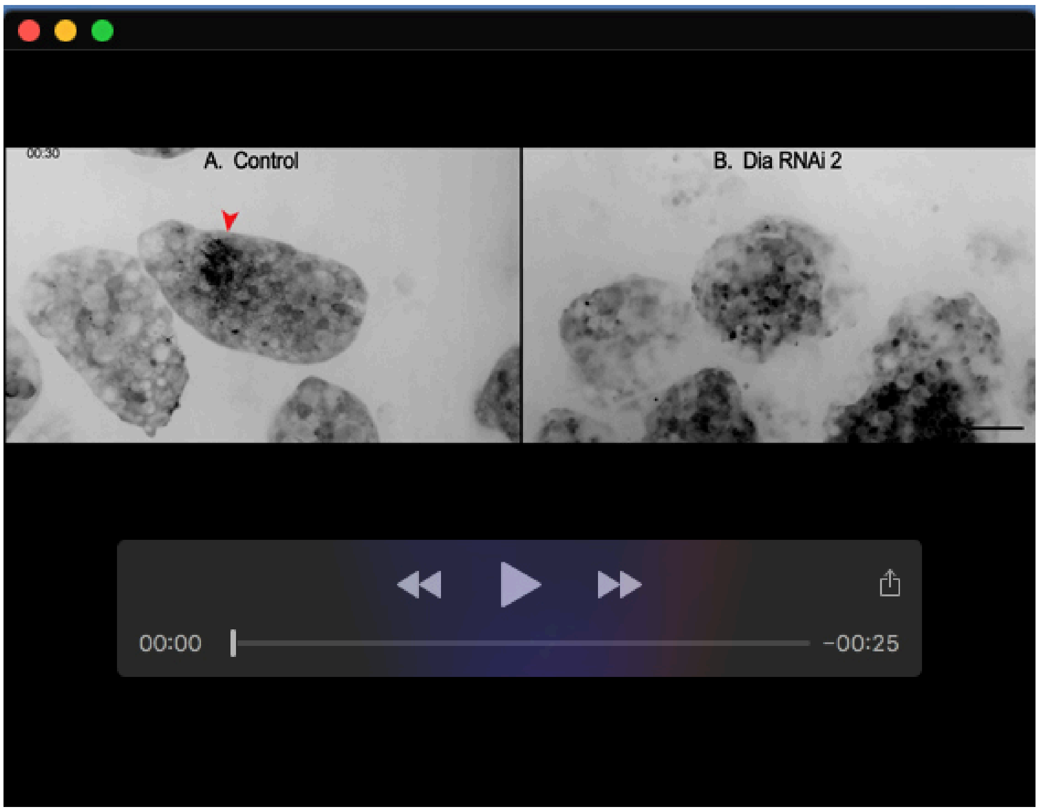

Movie 12. Dia is involved in actin wave formation – Related to Fig. 5F-I.

Confocal movies of actin dynamics and cell deformation from FBCs expressing Lsp2-Gal4+UAS-LifeAct-GFP+control (A) or +UAS-Dia RNAi2 (B). Red arrowhead points at actin wave. Elapsed time shown in top left corner in minutes:seconds. Scale bar, 20 µm.

Journal of Cell Science • Supplementary information

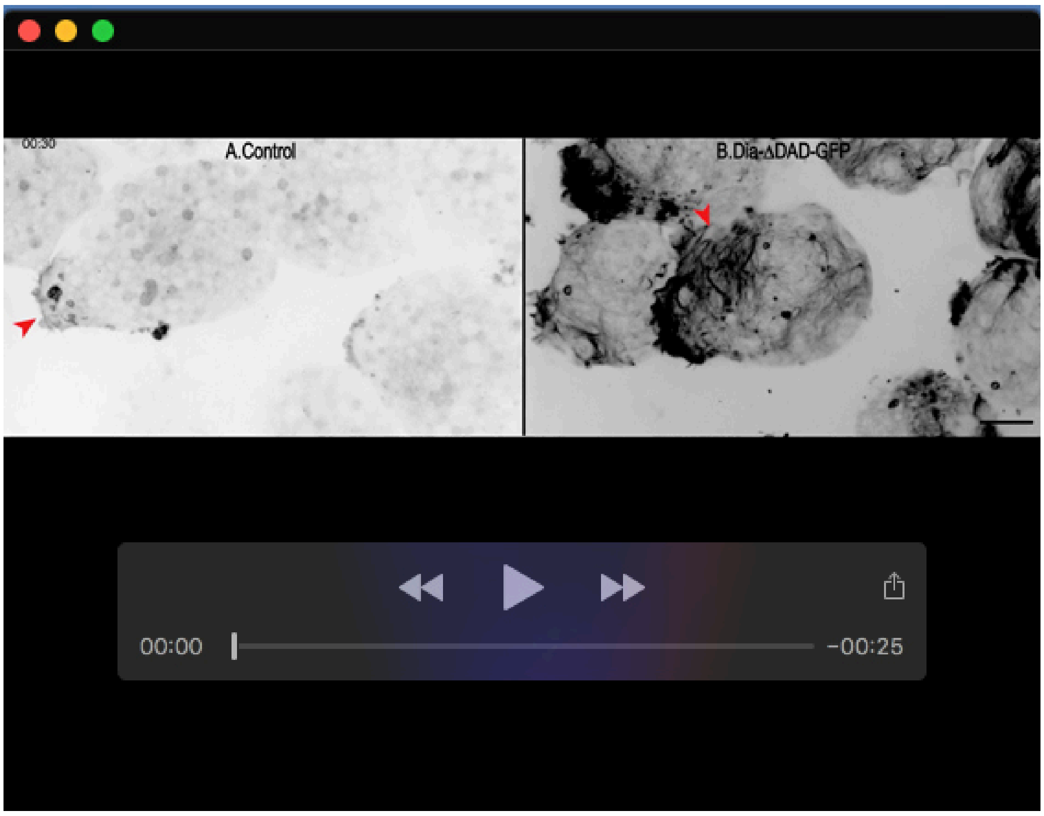

Movie 13. Dia overactivation increases cortical actin meshwork – Related to Fig. 5J-M.

Confocal movies of actin dynamics and cell deformation from FBCs expressing Lsp2-Gal4+UAS-LifeAct-Scarlet+control (A) or +UAS-Dia-ΔDAD-GFP (CA) (B). Red arrowhead points at actin waves (A) or highly dynamic actin swirls (B). Elapsed time shown in top left corner in minutes:seconds. Scale bar, 20 µm.

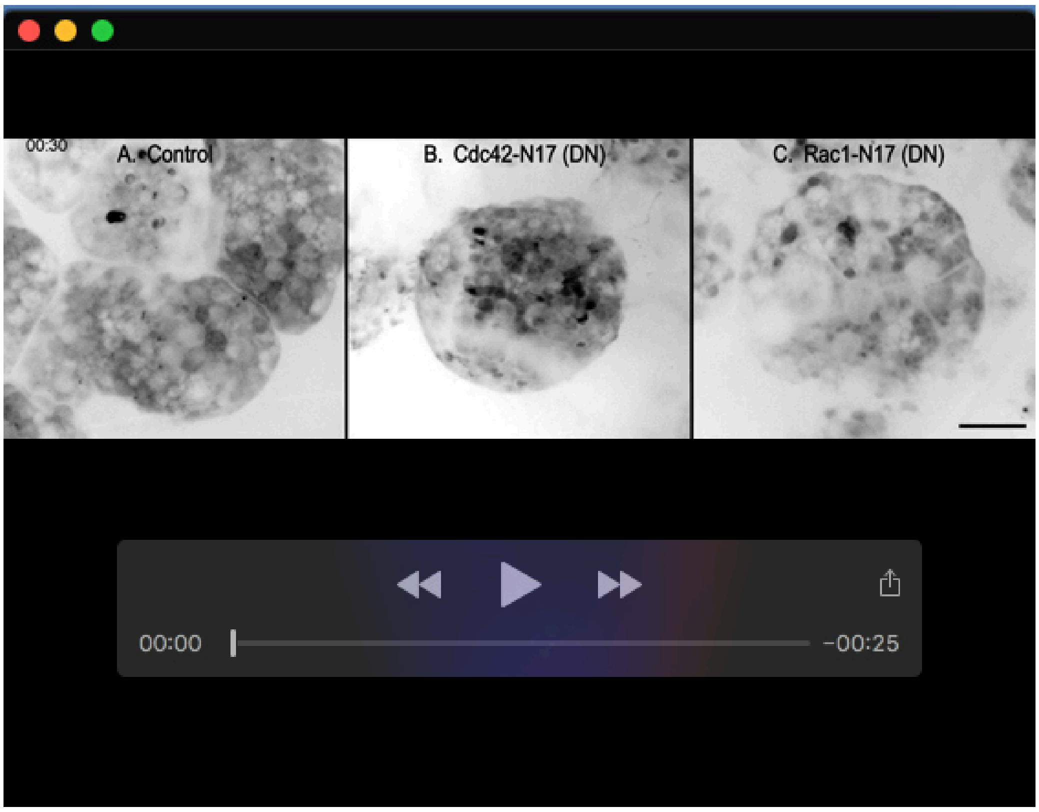

Movie 14. Cdc42 and Rac1 are involved in actin wave formation and fat body cell deformations – Related to Fig. 6I-N.

Confocal movies of actin dynamics and cell deformation from FBCs expressing Lsp2-Gal4+UAS-LifeAct-GFP+control (A), +UAS-Cdc42-N17 (DN) (B) or +UAS-Rac1-N17 (DN) (C). Red arrowhead points at actin wave. Elapsed time shown in top left corner in minutes:seconds. Scale bar, 20 µm.

Journal of Cell Science • Supplementary information

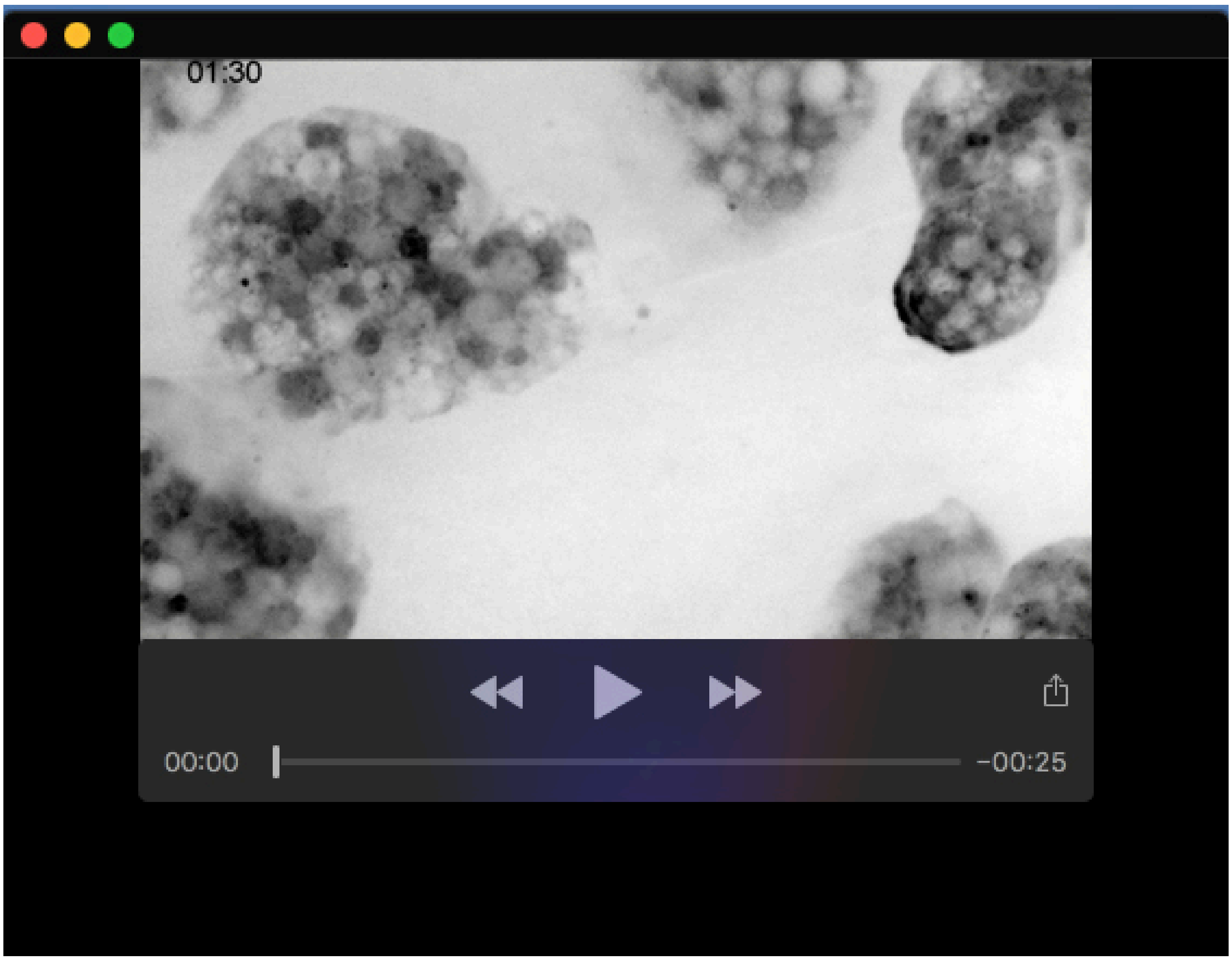

Movie 15. Actin wave leads to cell deformation of fat body cell – Related to Fig. 7A-D.

Confocal movie a Lsp2-Gal4+UAS-LifeAct-GFP expressing FBC. Red arrowheads show actin waves. Elapsed time shown in top left corner in minutes:seconds. Scale bar, 20 µm.

Journal of Cell Science • Supplementary information
