# Supplementary material for: Cell deformations generated by stochastic actomyosin waves drive in vivo random-walk swimming migration
Source: J Cell Sci. 2025 May 21;138(10):jcs263787. doi: 10.1242/jcs.263787 (PMC12148041; doi:10.1242/jcs.263787)
Supplement: Supplementary file 1 [file joces-138-263787_review_history.pdf]

Journal of Cell Science

Cell deformations generated by stochastic actomyosin waves drive *in vivo* random-walk swimming migration

Cyril Andrieu, Bren Hunyi Lee and Anna Franz
DOI: 10.1242/jcs.263787

Editor: Michael Way

Review timeline
Original submission: 11 December 2024
Editorial decision: 27 January 2025
First revision received: 26 March 2025
Accepted: 31 March 2025

Original submission

First decision letter

MS ID#: jcs.263787

MS TITLE: Cell deformations generated by stochastic actomyosin waves drive in vivo random-walk swimming migration

AUTHORS: Anna Franz; Cyril Andrieu; Bren Hunyi Lee
ARTICLE TYPE: Research Article

We have now reached a decision on the above manuscript.

To see the reviewers' reports and a copy of this decision letter, please go to:

As you will see, the reviewers raise a number of criticisms that prevent me from accepting the paper at this stage. They suggest, however, that a revised version might prove acceptable, if you can address their concerns. I feel many of the reviewers concerns can be addressed with text clarifications and will not require additional experiments. If you think that you can deal satisfactorily with the criticisms on revision, I would be pleased to see a revised manuscript.

Reviewer 1

Advance summary and potential significance to field

Here, Andrieu et al., interrogate the role of the key cytoskeletal players (Rho-GTPases, nucleators & actomyosin) in a distinct mode of cellular swimming, all within the in vivo context of the Drosophila pupa, revealed through exquisite live-imaging. In contrast to the mesenchymal migration historically studied in vitro, it is clear different cells can move in many weird and wonderful ways, undoubtedly reflecting the diversity and complexity encountered in vivo. In particular, the motility of "atypical" cells (such as the large, nonadherent fat body cells studied here) are understudied. Furthermore, the environmental context that cells find themselves in undoubtedly has huge influence on the way of they move, the complexities of which are impossible to fully recapitulate in vitro. Thus, the in vivo nature of these studies adds significant weight to the observations documented within this manuscript.

© 2025. Published by The Company of Biologists under the terms of the Creative Commons Attribution License (https://creativecommons.org/licenses/by/4.0/).

While the complete picture of how these cells convert their documented actin waves into locomotion ultimately remains out of reach here, this work does mechanistically define the involvement of the key cytoskeletal regulators in the actin waves and implicates their role within this unique mode of motility. Therefore, I believe this work to be of significant value to the field.

The results presented in this manuscript are well presented, if slightly laboriously detailed at times, but at no point overstated.

As such, I recommend this manuscript is suitable for publication in the Journal of Cell Science with minor corrections, detailed below:

Comments for the author

Major points:

1) In the various perturbations, what happens to overall fat body developmental dispersal? In the widefield tracking, it appears that while these perturbations often suppress motility, the dispersal of the FBCs into the head appears normal. If so, do the authors believe that this is due to partial suppression or related to the point below?

2) The inhibition of actomyosin suppresses motility by 50-65%, strongly implicating actomyosin in FBC motility. But what accounts for the remaining 50-35%? Is this residual motility passive? As part of their reasoning that wildtype FBC motility is active, the authors convincingly argue that the disparate direction of the tracks implies that the cells are not being moved passively by the hemolymph. What of the residual tracks when actomyosin is inhibited?

3) The term "super-resolution" is used very loosely within the field, particularly within the light microscopy community. It is not my place to define what is meant by this term, but I am not convinced the images presented here are "super-resolution". Beyond semantics, this is important because the authors argue they are directly visualising 'branched' actin', arising from the activity of the Arp2/3 complex. While not necessarily undercutting their ultimate conclusions, this statement is simply not true. At this resolution they are visualising (presumably) cross-linked actin bundles. While these are potentially in part derived from the remodelling of dendritic actin networks generated from the activity of the Arp2/3 complex (as suggested by the genetics), they are not the branched networks themselves.

In general, more care should be taken with word choice throughout so as to avoid confusion, as the field places special meaning on, at first glance, otherwise seemingly generic descriptive words:

- "Branched" actin (lines 201, 323) should be reserved for the dendritic network arising from the activity of the Arp2/3 complex. Instead, I suggest "mesh" or "criss-crossing" Even "cross-linked" implies the involvement of actin cross-linkers not determined here.

-"Parallel" actin filaments (line 296) should also be avoided as the orientation of F-actin (parallel vs. anti-parallel) relative to one another has important meaning and has not been determined here. Instead, I suggest "aligned".

4) It is unclear to me how many distinct actin networks the authors are proposing contribute to this motility. I.e. is it actomyosin alone, wherein the role of the nucleators is simply to generate/maintain a dynamic cortical mesh which is subsequently acted upon by myosin II to generate force? Or do the nucleators (such as Arp2/3) actively generate a wave of actin polymerisation which moves across and deforms the cell (akin to a protrusion) before reaching rear where confined actomyosin activity acts upon this actin to instigate a retraction? Or something inbetween, wherein the wave gradually accumulates myosin II which then is maximally activated when it reaches rear with highest RhoA activity? This might be simply addressed textually/in model (see below), but a valuable experiment might be to colocalise zipper-GFP with lifeact-scarlet in order to distinguish between the above possibilities.

© 2025. Published by The Company of Biologists under the terms of the Creative Commons Attribution License (https://creativecommons.org/licenses/by/4.0/).

5) The concluding diagram (Figure 7N) is slightly underwhelming and doesn't do credit to the work undertaken. While more detail is highlighted in the accompanying flow chart in Figure 7O, this doesn't convey the spatial pattern of (e.g.) RhoA activity the authors have documented.

6) As beautiful as they all are, not all of the accompanying movies are necessary. In particular, many of the low mag tracking movie panels could be dropped (e.g. movie 6: showing both the DN and RNAi is unnecessary. Likewise, movies 9, 12 and 15. Perhaps the most pertinent movies across all these could be combined into one movie).

Minor points:

1) This work focusses on the larval fat body. The adult fat body are also undergoing a morphologically distinct, developmental migration during metamorphosis and so, for clarity, it should be stated at least once at the start that this work focusses on the larval fat body cells.

2) Second panel of 3F it is difficult to make out the "1" of the time stamp

3) In Figure legend 7E & S4A, the indicated yellow areas are in fact green.

4) In their introduction to Rho kinase (line 256), it might be more accessible to firstly refer to mammalian "ROCK" before highlighting it's Drosophila homolog ("ROK").

5) "In contrast, swimming FBCs have no apparent front rear-polarity" (line 449) Given tightly localised RhoA activity documented (albeit transient), I'm not sure I agree with this. Furthermore, polarity is difficult define here given these cells are migrating randomly.

6) In methods, can measurements of "straightness" be better defined (i.e. is this equivalent of "directionality"?)

7) In analysis of "Actin wave number and FBC deformation" and elsewhere, it is stated this is done "blindly". I take this to mean the different z-projections of the various genotypes were blinded before analysis. If so, please do specify this for clarity.

8) Statistics Gaussian distribution was assessed to test normality of datasets.

9) Please add grant number to Bloomington acknowledgement (NIH P40OD018537). Bloomington asks to include following statement "Stocks obtained from the Bloomington Drosophila Stock Center (NIH P40OD018537)" and use of the exact phrase might help it gain the acknowledgement it deserves. Likewise, please cite Flybase if used in this study.

<u>Reviewer 2</u>

Advance summary and potential significance to field

This is an interesting and well put together paper that addresses the mechanisms underlying the fascinating motility exhibited by fat body cells in Drosophila pupae. The findings are novel and build upon the previous observations from this lab concerning the wound responsiveness of these cells in the fluid phase of the pupal circulation and their contributions to immune defences. It is a beautiful paper containing some lovely imaging across a range of microscopy systems and the figures are particularly nicely put together. There is some nice characterisation of the cell biology of fat body cells and the interesting "actin waves" they contain as they move around. A nice introduction places what could otherwise maybe a little bit of a niche observation in the context of the differing modes of migration we more commonly see. The results sections establish some new techniques/pipelines applied to the system before investigating some common and well characterised actin regulators and they impact they make on the movement of these cells and their actin dynamics. In general, the conclusions that are made seem well supported with the roles of these regulators addressed using the excellent tools available within the fly model. Some relatively minor changes or further experiments may well strengthen this paper further and enable the

© 2025. Published by The Company of Biologists under the terms of the Creative Commons Attribution License (https://creativecommons.org/licenses/by/4.0/).

conclusions and flow of the paper to be further improved but in my opinion this is a good paper already that would be suitable for this journal.

Comments for the author

Major points to consider

1. I found myself asking how the actin waves were tied to migration and productive movement as early as in Figure 1. Fig 1 features some really nice characterisation but when statements such as the following were made I wanted to know more:

"We found that the waves consistently peaked at the rear of the cell (Figure 2B, 91% of waves in rear half, front-rear axis determined from centroid displacement vector). This resulted in cell rear retraction and the fast propulsion of the cell body in the opposite direction."

This is actually addressed in Fig 7, and I did wonder whether some of the quantification that looks at this should come earlier in the paper, so the reader wasn't maybe thinking that FBC motility was purely as a result of the contraction at the rear, rather than via the cycling between the pear and spherical states that seems to be the model being proposed. I felt I was reading the paper from the wrong perspective and as a result was corrected by the data in Fig 7, so maybe 7A-G should come in an earlier figure as part of the initial characterisation?

In the early part of the paper there were some rather vague descriptions of the movies and figures, e.g., Line 197 that "sometimes clearly moved" where quantification such as correlations between actin waves and migration or frequency of events would be helpful.

Similarly for lines 195-207 - this is beautiful imaging, but the text here is a bit descriptive and could do with a little more quantification and an idea of the frequency of the events being described and potentially some quantitative information on how these observations impact migration / speed / directionality etc.

Obviously, this is addressed at the end of the paper to a good extent so it may be an idea to reorder a little (though I am not set upon this a change that would need to be made). Or perhaps reference could be made to the later data at an earlier point in the ms instead?

2. Line 209 - "devoid of actin" - the authors may need to be a bit more cautious here as this seems unlikely - presumably the filaments that can be visualised are bundles rather than single filaments and it is unlikely that these areas are devoid so some caution may be required as there is not the ultrastructural information that you can get from e.g. EM to prove this.

3. Rok RNAi decreased speed and altered aspect ratio, but the authors claim that Rok contributes to regulation of actin waves (line 276-277). Can this be clarified? Maybe this is due to lack of efficacy of RNAi here? Can other approaches be tried to check? Inhibitors? How effective is RNAi of Rok in these cells - has that been checked? Obviously Rok is doing something, but maybe its role is less associated with the actin waves or maybe knockdown isn't strong enough so this is a milder phenotype (though the speed change is comparable to sqh manipulation which has a strong effect on actin waves). It seems migratory parameters and actin waves can be uncoupled and this should be investigated more given the prime importance ascribed to the latter.

4. Reproducibility - the figure legends should be modified to inform reader of how many cells/movies/pupae the selected images are representative of. Where number of cells are reported it should also be reported how many movies/different pupae these are taken from.

5. Given the uncoupling of speed changes and an effect on actin waves for Rok RNAi, it would be good to see equivalent quantification of Cdc42 and Rac RNAi as per Fig 6 O and P, as it is good not to have an overreliance on dominant negative constructs.

6. Lines 321-327: the implication here is that these branches are Arp2/3 and or Wasp-dependent. It would therefore make this more sound if imaging of the actin network as per Figure 2 could be shown for knockdown of Arp2/3 and Wasp manipulations.

© 2025. Published by The Company of Biologists under the terms of the Creative Commons Attribution License (https://creativecommons.org/licenses/by/4.0/).

7. When talking about cell confinement in the discussion (lines 411-424), some discussion of the properties of the hemolymph should be added - if this was quite viscous for example, it could simulate a more confined environment and these sort of biophysical parameters are likely to impact some of the observations here. Could you purify these cells and watch them swim in different fluids with different viscosities (not a suggested experiment for this paper!) If the fluid is highly viscous it could enable chimneying to occur as the fluid cannot be pushed back so easily.

8. Does altered patrolling behaviour have any impact on organisam physiology? Can this be discussed, if only to remind the reader of the previous wound-orientated paper?

Minor corrections / points

133 extent rather than extend

Line 218 "in contrast" not needed here

Supplementary Fig 1b - straightness spelling error

Figure 2d perhaps some tracing of these structures (actin bindles / filaments) to make a schematic representation might show this network better?

Fig 3 k, m, o - Authors may want to add centroid + tracks to show movement more effectively?

521 Dm should be in italics as should genotypes

Fig 6F - the cells shown in this panel seems a bit of an odd example to show - the actin wave seems to move through the cell and isn't coupled to movement, or is it that this is actually two cells close together? Can a better example be found?

<u>Reviewer 3</u>

Advance summary and potential significance to field

In the manuscript 'Cell deformations generated by stochastic actomyosin waves drive in vivo random-walk swimming migration', Andrieu et al. study swimming migration by pupal Drosophila fat body cells (FBCs) in vivo. They show that stochastic actin activity leads to cell deformation that is involved in cell migration. The small RhoGTPases Rho1, Cdc42 and Rac1 are involved in this process, which also requires formin-driven actin polymerisation.

The authors show that Rho1 activity in the current rear of the cell leads to a contractile event and an actin wave. The leads both to cell elongation and cell rounding. This cell shape change is involved in cell migration.

This is a very interesting manuscript that describes FBC behaviour and explores the role of the molecular basis underlying FBC cell migration. FBCs use a very interesting mode of movement, which makes them a very good novel model to study non-mesenchymal cell migration. The observation that contraction can lead both to cell elongation and cell rounding is intriguing, as is the observation that the speed of migration correlates with the extent of cell aspect ratio change.

Comments for the author

1. From the presented data in Fig. 1F,G,H it is not sufficiently clear whether cells swim or whether they temporarily interact with a substratum to propel themselves forward. In the dorsal thorax, cells are quite crowded, so contact appears likely to occur. In the ventral thorax, the space is limited, so although cells are sparser, they may still encounter a substratum frequently.
- The sample size is rather small with n=7. More cells should be analysed. Also, how many pupae were analysed?

© 2025. Published by The Company of Biologists under the terms of the Creative Commons Attribution License (https://creativecommons.org/licenses/by/4.0/).

- Using % of surface area to assess contact is slightly misleading, as a small contact point may suffice to propel a cell forward.
- A short contact with the substratum may be sufficient to propel a cell forward. Thus, cells may not be in contact with a substratum for long time periods. Doing the analysis using live imaging would be more suitable to assess how often cells interact with their environment. In Movie 4 it appears as if FBCs slide along each other without much interaction - this could be explored more, also showing the interaction of FBCs with other cells in the haemolymph and with the epidermis.

2. I am not sure whether 'actin wave' is the best description for what the FBCs' actin network does. Indeed, I find it a little confusing. The authors show that the FBCs contract at their current back (Rho1, Rok and Sqh data). In combination with actin recruitment, this contraction could lead to a movement of part of the cells' actin network by convection (indeed, in Movie 5 one can see moving actin bundles). Alternatively, a contractile wave could move through the cell. Then, the most important aspect of the FBCs behaviour would be an actomyosin contraction rather than an actin wave.
I think that a more careful analysis of what the FBCs are doing would help to better understand the behaviour of their cytoskeleton:
- From the figure panels (Fig. 2) and the movies presented, it does not become sufficiently clear what a wave and what a pulse is. This could be better explained and quantified.
- Line 193 - 'This resulted in cell rear retraction and the fast propulsion of the cell body in the opposite direction' - this statement is not sufficiently supported by the data. The correlation of contractility and speed/direction of migration need to be analysed.
- Movie 5 - The phenomenology of the contractile event needs to be described in more detail and across more cells. Due to the variability of what FBCs do, presenting a few examples is not sufficient.
- Line 333 - 'the effects that actin waves had on cell shape appeared to be very complex and highly variable.' - It would be good to give the numbers for the various described phenotypes.
- At the beginning of Movie 5, it looks like a contractile cortical network is contracting and thereby sliding along the cell, squeezing it. This resembles the polarisation of the early C. elegans embryo (Munro et al., 2004). This appears to be different compared to the more local events that the authors describe. How often is this behaviour observed? Also, in Line 432 the authors say - 'First, the cortical actin network in FBCs rarely ever covers the whole cell surface, not even before wave formation'. - this is slightly confusing as the beginning of Movie 5 appears to show exactly this, suggesting that this is a more common occurrence.

3. In the discussion, the authors state that 'Waves can either lead to cell elongation or to rounding' (Line 452). Only here (and in Fig. 7N), it becomes clear that what appears to be happening is that a first contraction is elongating the cell and that a second contraction is rounding it up again. This behaviour should be visible in the data, when correlating actin activity with cell shape change for individual cells. This analysis should be included. In Fig. 7E, such a trend is visible as two actin peaks correlate with one peak and one trough of the aspect ratio data.

4. It remains unclear how the observed FBC behaviour (cell shape change) leads to cell movement. The authors show that dynamic cell deformations are involved in FBC migration (affecting migration speed), however, they do not uncover the mechanism by which the deformations propel the cells. Also, the authors cite Lim et al. (2013) to point out that 'nonreciprocal cell shape changes can theoretically drive force transmission in amoeboid migration', however, they do not discuss sufficiently how this relates to FBC movement.
- Line 330 - 'we next wanted to investigate how these waves drive cell propulsion by looking at the effects that waves have on cell shapes' - This sentence implies that waves are driving cell propulsion, but this has not been formally shown.
- Line 460 - 'Some might compress the rear of a round cell resulting in hydrostatic pressure driven cell elongation and cell propulsion.' - this sentence causally connects cell elongation and cell propulsion, however, this connection is not sufficiently made.
- It is possible that contraction at the rear drives membrane flow which in turn is propelling the cell. This could be discussed in more detail. Do the authors have any evidence that excludes or shows that membrane flow occurs?

Other comments:

© 2025. Published by The Company of Biologists under the terms of the Creative Commons Attribution License (https://creativecommons.org/licenses/by/4.0/).

Line 324 - 'In agreement with this, we found that RNAi against Wasp as well as against Arp2 or Arp3 led to a significant decrease in FBC migration speed (Suppl.Figure 3). This suggests that Wasp and the Arp2/3 complex are involved in FBC migration and might be the effectors of Cdc42.' - This is not sufficient evidence to suggest that Wasp and Arp2/3 may be the effectors of Cdc42. Also, it would be good to also look at the LifeActin-GFP phenotype in these experiments. How is the actin cytoskeleton affected?
Overall, the role of Rac1 and Cdc42 in the process remains largely unclear.

Line 182 - 'Using LifeAct-GFP to study actin dynamics in swimming FBCs, we found that periodic cortical actin waves were present in all FBCs in the pupal head' - How many cells were assessed?

The third results paragraph states that 'Random-walk FBC migration is driven by periodic cortical actin waves at the cell rear'. This is in contradiction with the statement in the discussion that 'swimming FBCs have no apparent front rear-polarity' (Line 449). This needs to be made clearer - do the authors mean that a contraction is establishing a new rear?

Minor comments
The first introduction paragraph could have a reference relating more specifically to amoeboid migration.

The following sentence could have references that more specifically makes the point stated (maybe a suitable review on amoeboid migration) - line 45 'Since amoeboid migration does not feature lamellipodia or filopodia, it is thought to be mostly independent of Rac1, Cdc42 or Arp2/3-driven branched actin formation, which mediate the generation of these front protrusions in mesenchymal migration (Ridley, 2001, Ridley, 2015)'.

Line 91 - 'completely' is not necessary.

Line 540 - what is the resolution of the super-resolution used?

First revision

Author response to reviewers' comments

Reviewer 1: SUMMARY OF THE ADVANCE MADE IN THIS PAPER AND ITS POTENTIAL SIGNIFICANCE TO THE FIELD

Here, Andrieu et al., interrogate the role of the key cytoskeletal players (Rho-GTPases, nucleators & actomyosin) in a distinct mode of cellular swimming, all within the in vivo context of the Drosophila pupa, revealed through exquisite live-imaging. In contrast to the mesenchymal migration historically studied in vitro, it is clear different cells can move in many weird and wonderful ways, undoubtedly reflecting the diversity and complexity encountered in vivo. In particular, the motility of "atypical" cells (such as the large, nonadherent fat body cells studied here) are understudied. Furthermore, the environmental context that cells find themselves in undoubtedly has huge influence on the way of they move, the complexities of which are impossible to fully recapitulate in vitro. Thus, the in vivo nature of these studies adds significant weight to the observations documented within this manuscript.

While the complete picture of how these cells convert their documented actin waves into locomotion ultimately remains out of reach here, this work does mechanistically define the involvement of the key cytoskeletal regulators in the actin waves and implicates their role within this unique mode of motility. Therefore, I believe this work to be of significant value to the field.

The results presented in this manuscript are well presented, if slightly laboriously detailed at times, but at no point overstated.

As such, I recommend this manuscript is suitable for publication in the Journal of Cell Science with

© 2025. Published by The Company of Biologists under the terms of the Creative Commons Attribution License (https://creativecommons.org/licenses/by/4.0/).

minor corrections, detailed below:

SUGGESTIONS TO AUTHORS Major points:

1) In the various perturbations, what happens to overall fat body developmental dispersal? In the widefield tracking, it appears that while these perturbations often suppress motility, the dispersal of the FBCs into the head appears normal. If so, do the authors believe that this is due to partial suppression or related to the point below?

Thanks for enquiring about this. The various genetic manipulations that strongly block fat body migration do not appear to affect the viability of the animals, which survive into adulthood. However, we would expect them to have a defect in the recruitment of FBCs to wounds in the pupa, as we have previously shown this for expression of DN-Zip-YFP with Lpp-Gal4 in the fat body (Franz et al. 2018). We have now added this point to the discussion (line 410-415):

'What is the function of fat body migration during metamorphosis? Blocking fat body cell migration through the various genetic manipulations used in our study resulted in a strong reduction in speed. Yet this did not result in lethality since animals survived into adulthood, suggesting that FBC migration is not essential for pupal development. However, we would expect migration defects to result in defects in recruitment of FBCs to wounds, as we showed before for Lpp-Gal4+UAS-DN-Zip-YFP expression (Franz et al., 2018).'

We further think that it would be important to look at whether blocking migration of FBCs might negatively affect other processes/organs, but this is beyond the scope of this paper.

2) The inhibition of actomyosin suppresses motility by 50-65%, strongly implicating actomyosin in FBC motility. But what accounts for the remaining 50-35%? Is this residual motility passive? As part of their reasoning that wildtype FBC motility is active, the authors convincingly argue that the disparate direction of the tracks implies that the cells are not being moved passively by the hemolymph. What of the residual tracks when actomyosin is inhibited?

Thanks for pointing this out. Our data suggest that most of the movement we see of FBC migration is active since blocking any of the key actin regulators leads to a strong speed reduction of around 54-74%. We suspect that the remaining movement is likely due to either incomplete knockdown/gene disruption and/or due to passive movement of cells driven by hemolymph flows. We have now added this to the results (lines 142-143).

'We suspect that the remaining movement might be due to incomplete block of gene function or/and hemolymph flows. This suggests that the observed movement of FBCs in all areas of the pupal body is largely driven by active cell migration.'

Moreover, we have noticed in some of the conditions that cells move in synchrony in certain areas of the pupa (e.g in the thorax upon RhoDN expression) in support of the presence of local hemolymph flows that can move migration-defective FBCs.

3) The term "super-resolution" is used very loosely within the field, particularly within the light microscopy community. It is not my place to define what is meant by this term, but I am not convinced the images presented here are "super-resolution". Beyond semantics, this is important because the authors argue they are directly visualising 'branched' actin', arising from the activity of the Arp2/3 complex. While not necessarily undercutting their ultimate conclusions, this statement is simply not true. At this resolution they are visualising (presumably) cross-linked actin bundles. While these are potentially in part derived from the remodelling of dendritic actin networks generated from the activity of the Arp2/3 complex (as suggested by the genetics), they are not the branched networks themselves.

We apologise for not being more careful with our choice of words and agree that this might be misleading. We have now changed the wording (see next point). We agree that our type of super resolution imaging cannot reveal branched actin filaments. However, our results showing that

© 2025. Published by The Company of Biologists under the terms of the Creative Commons Attribution License (https://creativecommons.org/licenses/by/4.0/).

Arp2/3 knockdown reduces migration speed together with our newly added results showing that Arp2RNAi reduces actin wave numbers (Suppl Figure 5K) suggests a role of branched actin.

Regarding the term super-resolution, we used an NSPARC microscope which clearly provided a more detailed structural information of the actin network than our confocal microscopes. Calculating the point spread function from fluorescent microspheres we found the optimal resolution on this NSPARC microscope to be 110nm. The smallest actin structures that we observed in our 'super-resolution' images and movies of FBCs were 180nm. We have now added more information regarding the parameters used for super-resolution in the methods (lines 560-571).

In general, more care should be taken with word choice throughout so as to avoid confusion, as the field places special meaning on, at first glance, otherwise seemingly generic descriptive words:

- "Branched" actin (lines 201, 323) should be reserved for the dendritic network arising from the activity of the Arp2/3 complex. Instead, I suggest "mesh" or "criss-crossing" Even "cross-linked" implies the involvement of actin cross-linkers not determined here.

We agree. As suggested by the reviewer, we have now changed the text and just use the word mesh instead of branched (lines 206, 332 and figure legend 2 in line 686).

-"Parallel" actin filaments (line 296) should also be avoided as the orientation of F-actin (parallel vs. anti-parallel) relative to one another has important meaning and has not been determined here. Instead, I suggest "aligned".

Again, we agree and refer to it now as 'aligned' instead of 'parallel' (line 303).

4) It is unclear to me how many distinct actin networks the authors are proposing contribute to this motility. I.e. is it actomyosin alone, wherein the role of the nucleators is simply to generate/maintain a dynamic cortical mesh which is subsequently acted upon by myosin II to generate force? Or do the nucleators (such as Arp2/3) actively generate a wave of actin polymerisation which moves across and deforms the cell (akin to a protrusion) before reaching rear where confined actomyosin activity acts upon this actin to instigate a retraction? Or something inbetween, wherein the wave gradually accumulates myosin II which then is maximally activated when it reaches rear with highest RhoA activity? This might be simply addressed textually/in model (see below), but a valuable experiment might be to colocalise zipper-GFP with lifeact-scarlet in order to distinguish between the above possibilities.

We agree that it is currently still unclear how exactly the dynamic actin network becomes organised in migrating FBCs. Theoretically, it could be that actin is evenly distributed in the whole cortex and local myosin activation through Rho1 at the future rear contracts the network and hence pulls the cortical actin to the rear and away from the front. However, we do not see such actin behaviour very often, at least when looking at the network of actin bundles using super resolution microscopy. Moreover, 'actin waves' usually travel along the cortex before peaking somewhere which is difficult to reconcile with this model. We currently favour a model where a denser actin meshwork forms locally (through increased/new actin filament nucleation and/or actin bundling and/or myosin-activation). This denser actin area ('wave') might then travel across the cortex by promoting new nucleation/bundling/myosin activation on one side and depolymerisation/unbundling on the other side. The wave then travels along the cortex until it reaches a site where myosin strongly contracts the actin wave to cause a cell deformation. However, we suspect that myosin binding and weaker contraction already happens while the wave travels. Indeed, in some cases we have seen a belt of actin constricting the cell as it moved rearwards in a peristaltic behaviour (e.g. new movie 15 minute14-17.30).

To try to gain a better understanding of this process, we have tried to do super-resolution microscopy of Sqh-GFP (Sqh-GFP protein trap line or Sqh>Sqh-GFP in sqh mutant background) together with Lsp2-Gal4+UAS-Lifeact-GFP in FBCs. The fluorescent signal of Sqh-GFP was unfortunately too weak to achieve the desired resolution (super-resolution). This prevented us

© 2025. Published by The Company of Biologists under the terms of the Creative Commons Attribution License (https://creativecommons.org/licenses/by/4.0/).

from analysing actin together with myosin dynamics over time as the actin wave forms and travels using super resolution. However, when we used higher resolution confocal microscopy, we consistently saw Sqh-GFP at the cell rear where the actin wave peaked, showing at least partial colocalisation. Hence, at the moment our results do not point to a particular mode of wave formation. We suspect that multiple pathways play a role in regulating actin dynamics, wave formation and contraction. The complexity we see might be related to the influence that Rac1 and Cdc42 have on wave behaviour. The details of this will need to be dissected through future work, in addition to the thorough characterisation for the actin and myosin behaviour.

5) The concluding diagram (Figure 7N) is slightly underwhelming and doesn't do credit to the work undertaken. While more detail is highlighted in the accompanying flow chart in Figure 7O, this doesn't convey the spatial pattern of (e.g.) RhoA activity the authors have documented.

Thanks for the suggestion. We have now adjusted the model in Figure 7N and included the proposed localisation of RhoA and Rok at the rear.

6) As beautiful as they all are, not all of the accompanying movies are necessary. In particular, many of the low mag tracking movie panels could be dropped (e.g. movie 6: showing both the DN and RNAi is unnecessary. Likewise, movies 9, 12 and 15. Perhaps the most pertinent movies across all these could be combined into one movie).

We have taken this suggestion on board and have now removed some movies, where several different lines were used to block the function of the same gene. We removed the original movies 6C, movie 9C, D and E, movie 12B, movie 15C and F. Moreover, we have now merged most of the tracking movies (original movies 6, 9, 12 and 15) into one new movie 7A-H.

Minor points:

1) This work focusses on the larval fat body. The adult fat body are also undergoing a morphologically distinct, developmental migration during metamorphosis and so, for clarity, it should be stated at least once at the start that this work focusses on the larval fat body cells.

We apologise for not pointing this out in our original manuscript. We have now clarified this in the beginning of the results section in lines 110-113:

'For this, we focussed on the population of FBCs derived from the mesoderm during embryogenesis, the 'larval FB population' rather than the adult fat body population which undergoes a distinct, developmental migration during metamorphosis (Lei et al., 2023, Tsuyama et al., 2023).'

2) Second panel of 3F it is difficult to make out the "1" of the time stamp

We have adjusted this now in Figure 3F

3) In Figure legend 7E & S4A, the indicated yellow areas are in fact green.

We apologise for this mistake. We have adjusted this now in Figure legend 7E (line 792) & new Suppl. Figure 6A.

4) In their introduction to Rho kinase (line 256), it might be more accessible to firstly refer to mammalian "ROCK" before highlighting it's Drosophila homolog ("ROK").

Thanks for pointing this out correctly. In line 262, we now refer to mammalian ROCK first as suggested by the reviewer. In line 264, we added that ROK is the Drosophila homolog of ROCK.

© 2025. Published by The Company of Biologists under the terms of the Creative Commons Attribution License (https://creativecommons.org/licenses/by/4.0/).

'RhoA is generally known to regulate actomyosin contractility through Rho kinase (ROCK) and myosin II (Ridley, 2001, Ridley, 2015). We observed a strong reduction in FBC migration speed when we blocked Rok, the Drosophila homolog of ROCK (with two RNAi lines), myosin II light chain (with dominant-negative Sqh (Sqh-AA) and a Sqh-RNAi line), and myosin II heavy chain (with Zipper-DN-GFP; Figure 4A-G, Movie 7C-D).'

5) "In contrast, swimming FBCs have no apparent front rear-polarity" (line 449) Given tightly localised RhoA activity documented (albeit transient), I'm not sure I agree with this. Furthermore, polarity is difficult define here given these cells are migrating randomly.

We agree that polarity is difficult to define here. We have now toned this down in the text the following way:

The original version: 'In contrast, swimming FBCs have no apparent front rear-polarity. This is potentially due to a lack of cell-substrate adhesion that could instruct polarity. Maybe because of this, FBCs show a strikingly stochastic cell behaviour.'

The new version (lines 466-468): 'In contrast, swimming FBCs migrate by random walk. This is potentially due to a lack of cell-substrate adhesion that could reinforce polarity. Maybe because of this, FBCs show a strikingly stochastic cell behaviour.'

6) In methods, can measurements of "straightness" be better defined (i.e. is this equivalent of "directionality"?)

In the methods section (lines 591-594), we have now given more details on how the straightness was measured: 'The straightness was calculated by dividing total track displacement (shortest path from the first time point to the last time point) by actual track length (total track length from the first to the last point).'

Straightness measures the directional persistence. In some papers straightness has also been referred to as migration directionality ratio or meandering index.

7) In analysis of "Actin wave number and FBC deformation" and elsewhere, it is stated this is done "blindly". I take this to mean the different z-projections of the various genotypes were blinded before analysis. If so, please do specify this for clarity.

We have now given further detail in lines 608-609:

'Visual assessment of actin wave numbers was done blindly (by blinding the Z projections images of various genotypes before analysis).'

8) Statistics Gaussian distribution was assessed to test normality of datasets.

We have now added in line 639: 'Gaussian distribution was assessed to test normality of datasets.'

9) Please add grant number to Bloomington acknowledgement (NIH P40OD018537). Bloomington asks to include following statement "Stocks obtained from the Bloomington Drosophila Stock Center (NIH P40OD018537)" and use of the exact phrase might help it gain the acknowledgement it deserves. Likewise, please cite Flybase if used in this study.

Thanks for reminding us of this. We have now added in the methods in lines 532-533 'Stocks obtained from the Bloomington Drosophila Stock Center (NIH P40OD018537) were used in this study' *and in lines 545-546:* 'We used FlyBase (releases2020_06-2024_02) to find information on phenotypes/function/stocks/gene expression (etc).'

© 2025. Published by The Company of Biologists under the terms of the Creative Commons Attribution License (https://creativecommons.org/licenses/by/4.0/).

Reviewer 2:

This is an interesting and well put together paper that addresses the mechanisms underlying the fascinating motility exhibited by fat body cells in Drosophila pupae. The findings are novel and build upon the previous observations from this lab concerning the wound responsiveness of these cells in the fluid phase of the pupal circulation and their contributions to immune defences. It is a beautiful paper containing some lovely imaging across a range of microscopy systems and the figures are particularly nicely put together. There is some nice characterisation of the cell biology of fat body cells and the interesting "actin waves" they contain as they move around. A nice introduction places what could otherwise maybe a little bit of a niche observation in the context of the differing modes of migration we more commonly see. The results sections establish some new techniques/pipelines applied to the system before investigating some common and well characterised actin regulators and they impact they make on the movement of these cells and their actin dynamics. In general, the conclusions that are made seem well supported with the roles of these regulators addressed using the excellent tools available within the fly model. Some relatively minor changes or further experiments may well strengthen this paper further and enable the conclusions and flow of the paper to be further improved but in my opinion this is a good paper already that would be suitable for this journal.

Major points to consider

1. I found myself asking how the actin waves were tied to migration and productive movement as early as in Figure 1. Fig 1 features some really nice characterisation but when statements such as the following were made I wanted to know more:

"We found that the waves consistently peaked at the rear of the cell (Figure 2B, 91% of waves in rear half, front-rear axis determined from centroid displacement vector). This resulted in cell rear retraction and the fast propulsion of the cell body in the opposite direction."

This is actually addressed in Fig 7, and I did wonder whether some of the quantification that looks at this should come earlier in the paper, so the reader wasn't maybe thinking that FBC motility was purely as a result of the contraction at the rear, rather than via the cycling between the pear and spherical states that seems to be the model being proposed. I felt I was reading the paper from the wrong perspective and as a result was corrected by the data in Fig 7, so maybe 7A-G should come in an earlier figure as part of the initial characterisation?

In the early part of the paper there were some rather vague descriptions of the movies and figures, e.g., Line 197 that "sometimes clearly moved" where quantification such as correlations between actin waves and migration or frequency of events would be helpful.

Similarly for lines 195-207 - this is beautiful imaging, but the text here is a bit descriptive and could do with a little more quantification and an idea of the frequency of the events being described and potentially some quantitative information on how these observations impact migration / speed / directionality etc.

Obviously, this is addressed at the end of the paper to a good extent so it may be an idea to reorder a little (though I am not set upon this a change that would need to be made). Or perhaps reference could be made to the later data at an earlier point in the ms instead?

Thanks for this feedback. We agree that the initial characterisation of the actin waves in Figure 2 does not fully describe the complexity of the actin dynamics that we observe but rather provide a few representative examples of the type of behaviour that we see by confocal and super resolution microscopy. Importantly our quantifications in Figure 2B clearly show that actin waves very consistently peak at the rear of the cell (in relation to where the cell moves 2min later) suggesting that these waves at the rear are likely linked to the migration. We then show that these waves are indeed essential for driving migration since blocking regulators of the actin network block actin wave formation and contraction and strongly reduce migration speed. Finally, we characterise in much more detail how actin waves regulate cell shape and cell shape changes showing that the latter are key for the migration. In our opinion, the current order of the figures works best for the flow of the paper. Moreover, we are hesitant to refer to later parts of the results section already

© 2025. Published by The Company of Biologists under the terms of the Creative Commons Attribution License (https://creativecommons.org/licenses/by/4.0/).

in the earlier parts since this would be highly unusual to do. We hope the reviewer understands our hesitation of changing the order of the story.

2. Line 209 - "devoid of actin" - the authors may need to be a bit more cautious here as this seems unlikely - presumably the filaments that can be visualised are bundles rather than single filaments and it is unlikely that these areas are devoid so some caution may be required as there is not the ultrastructural information that you can get from e.g. EM to prove this.

We agree. We have now toned this down in the text in line 213-216 by saying instead: 'Overall, the meshwork of actin bundles seemed highly dynamic and usually did not cover the entire cortex of the cell, with parts of the surface being seemingly devoid of actin bundles. Actin waves moved along the cell surface to nearby areas previously devoid of actin bundles (Figure 2D'', Movie 6B).'

3. Rok RNAi decreased speed and altered aspect ratio, but the authors claim that Rok contributes to regulation of actin waves (line 276-277). Can this be clarified? Maybe this is due to lack of efficacy of RNAi here? Can other approaches be tried to check? Inhibitors? How effective is RNAi of Rok in these cells - has that been checked? Obviously Rok is doing something, but maybe its role is less associated with the actin waves or maybe knockdown isn't strong enough so this is a milder phenotype (though the speed change is comparable to sqh manipulation which has a strong effect on actin waves). It seems migratory parameters and actin waves can be uncoupled and this should be investigated more given the prime importance ascribed to the latter..

We agree that it remains puzzling why Rok RNAi reduces speed and cell aspect ratio, while it does not appear to reduce the number of actin waves while blocking myosin does. We suspect that Rok RNAi might be less effective in blocking Rok than Sqh-AA expression is in blocking myosin II function. It is hard to assess the efficiency of Rok RNAi, since we currently do not have a protocol to perform Q-PCRs on FBCs as the tissue cannot be dissected out of the animal.

We have already tried to explain this in the text (lines 280-283): 'This difference between Rok-RNAi and Sqh-AA, both in reducing speed (Figure 4G) and in reducing actin waves, could be explained either by myosin II getting activated by Rok as well as another kinase, or by the loss of function effect of Sqh-AA being stronger than the one of Rok-RNAi.'

However, to be more correct with our wording, we have removed from the text the suggestion that Rok regulates actomyosin contractions. The new version is found in lines 283-284 'Together, these data suggest that actomyosin contraction, regulated by myosin II, at least in part, contributes to the regulation of actin waves. ' *and in lines 289-291* 'In summary, actomyosin contractions in the cell rear regulated by myosin II are crucial for FBC deformations and contribute, to a lesser extent, to actin wave regulation during swimming migration.'

4. Reproducibility - the figure legends should be modified to inform reader of how many cells/movies/pupae the selected images are representative of. Where number of cells are reported it should also be reported how many movies/different pupae these are taken from.

We thank the reviewer for pointing out this. We have added the number of pupae in the figure legends.

5. Given the uncoupling of speed changes and an effect on actin waves for Rok RNAi, it would be good to see equivalent quantification of Cdc42 and Rac RNAi as per Fig 6 O and P, as it is good not to have an overreliance on dominant negative constructs.

Thanks for this suggestion. We have now quantified actin wave numbers, mean aspect ratio and absolute aspect ratio changes for Cdc42 RNAi and Rac1 RNAi which showed a significant reduction for all these parameters. This is now included in new Supp Fig 4 and mentioned in the text (lines 322-328 and 385-388).

© 2025. Published by The Company of Biologists under the terms of the Creative Commons Attribution License (https://creativecommons.org/licenses/by/4.0/).

'Moreover, we found that Cdc42-N17 (Figure 6I, K, O, Movie 14A and B) and Cdc42 RNAi-expression (Suppl. Figure 4A) decreased the number of actin waves in FBCs dramatically which led to cell rounding (Figure 6J, L and P and Suppl. Figure 4B). Rac1-N17- or Rac1 RNAi-expression also resulted in a moderate reduction in the number of actin waves (Figure 6I, M, O, Movie 14A and C and Suppl. Figure 4A) and cell rounding (Figure 6J, N and P and Suppl. Figure 4B) although the effects were less severe than upon Cdc42-N17- or Cdc42 RNAi-expression. '

'Finally, we tested whether these cell deformations were caused by contractile actin waves. Indeed, we found that disrupting the gene function of the key regulators of actin wave formation and contraction, Rho1, Rok, myosin II, Dia, Rac1 and Cdc42, strongly reduced mean absolute aspect ratio changes (Figure 7H-L and Suppl. Figure 4C).'

6. Lines 321-327: the implication here is that these branches are Arp2/3 and or Wasp-dependent. It would therefore make this more sound if imaging of the actin network as per Figure 2 could be shown for knockdown of Arp2/3 and Wasp manipulations.

We agree that our current data are not sufficient to demonstrate that branched actin is present in the actin waves. Our tracking data suggest that Wasp and Arp2/3 play a role in FBC migration. Moreover, we have some new data using confocal microscopy, showing that actin wave numbers are significantly reduced upon Wasp or Arp2 RNAi (new Suppl figure 5J, K-mentioned in lines 335-336), suggesting that Wasp and Arp2 play a role in regulating actin wave formation during FBC migration.

'Moreover, Wasp RNAi and Arp2 RNAi-expression reduced the number of actin waves (Suppl. Figure 5J, K). '

Unfortunately, the reviewer's suggestion of characterising the actin meshwork properties and potential changes upon Wasp or Arp2 RNAi was not possible. Doing this at high throughput using super- resolution microscopy is very time consuming due to the following limitations. When doing super resolution microscopy, we can only image a single cell at a time and can only image the top third of the cell to achieve the desired resolution. Unfortunately, this results in cells often leaving the field of view within less than a minute which prevents us from analysing a larger number of cells for quantifications. Moreover, assessing changes to the actin meshwork would require developing a new protocol to find the appropriate parameters that might best describe the meshwork properties. We also believe that this quantification would ultimately not answer whether real actin branching is present in WT and potentially reduced in Arp2 RNAi. Since we cannot give more direct evidence of branched actin being involved here, we have now adjusted this paragraph to tone down the wording. We have now changed the text and just use the word mesh instead of branched (lines 332).

7. When talking about cell confinement in the discussion (lines 411-424), some discussion of the properties of the hemolymph should be added - if this was quite viscous for example, it could simulate a more confined environment and these sort of biophysical parameters are likely to impact some of the observations here. Could you purify these cells and watch them swim in different fluids with different viscosities (not a suggested experiment for this paper!) If the fluid is highly viscous it could enable chimneying to occur as the fluid cannot be pushed back so easily.

Thanks for this interesting comment and suggestions. We have now added a sentence to the discussion to speculate on the potential effects of hemolymph viscosity on FBC migration (lines 437-438): 'Instead of confinement, the viscosity of the hemolymph might dictate the biophysical properties of the environment and hence enable fat body cell migration.'

So far we have focused our work on in vivo settings. However, as suggested, we are planning combining this with some ex vivo experiments for our future work.

8. Does altered patrolling behaviour have any impact on organisam physiology? Can this be discussed, if only to remind the reader of the previous wound-orientated paper?

© 2025. Published by The Company of Biologists under the terms of the Creative Commons Attribution License (https://creativecommons.org/licenses/by/4.0/).

We have already responded to this (see major point 1 from reviewer 1).

Minor corrections / points

133 extent rather than extend

We have corrected this now.

Line 218 "in contrast" not needed here

We removed "in contrast"

Supplementary Fig 1b - straightness spelling error

We have corrected this now.

Figure 2d perhaps some tracing of these structures (actin bindles / filaments) to make a schematic representation might show this network better?

Thanks for the suggestion. However, we are hesitant to do this. We think that the real data best reflect the actin network, the details of which are clearly visible, especially in the accompanying movie.

Fig 3 k, m, o - Authors may want to add centroid + tracks to show movement more effectively?

We are not sure if the reviewer is referring to Figure 4 K, M, O? We have tried adding this but due to the strongly reduced movement of cells in M and O the centroids there mostly overlap.

521 Dm should be in italics as should genotypes

We have written Drosophila melanogaster in italics in line 531.

Fig 6F - the cells shown in this panel seems a bit of an odd example to show - the actin wave seems to move through the cell and isn't coupled to movement, or is it that this is actually two cells close together? Can a better example be found?

We are not sure what the reviewer is referring to here since Figure 6F shows a tracking image.

Reviewer 3: SUMMARY OF THE ADVANCE MADE IN THIS PAPER AND ITS POTENTIAL SIGNIFICANCE TO THE FIELD

In the manuscript 'Cell deformations generated by stochastic actomyosin waves drive in vivo random- walk swimming migration', Andrieu et al. study swimming migration by pupal Drosophila fat body cells (FBCs) in vivo. They show that stochastic actin activity leads to cell deformation that is involved in cell migration. The small RhoGTPases Rho1, Cdc42 and Rac1 are involved in this process, which also requires formin-driven actin polymerisation.

The authors show that Rho1 activity in the current rear of the cell leads to a contractile event and an actin wave. The leads both to cell elongation and cell rounding. This cell shape change is involved in cell migration.

This is a very interesting manuscript that describes FBC behaviour and explores the role of the molecular basis underlying FBC cell migration. FBCs use a very interesting mode of movement, which makes them a very good novel model to study non-mesenchymal cell migration. The

© 2025. Published by The Company of Biologists under the terms of the Creative Commons Attribution License (https://creativecommons.org/licenses/by/4.0/).

observation that contraction can lead both to cell elongation and cell rounding is intriguing, as is the observation that the speed of migration correlates with the extent of cell aspect ratio change.

SUGGESTIONS TO AUTHORS

1. From the presented data in Fig. 1F,G,H it is not sufficiently clear whether cells swim or whether they temporarily interact with a substratum to propel themselves forward. In the dorsal thorax, cells are quite crowded, so contact appears likely to occur. In the ventral thorax, the space is limited, so although cells are sparser, they may still encounter a substratum frequently.
- The sample size is rather small with n=7. More cells should be analysed. Also, how many pupae were analysed?
- Using % of surface area to assess contact is slightly misleading, as a small contact point may suffice to propel a cell forward.
- A short contact with the substratum may be sufficient to propel a cell forward. Thus, cells may not be in contact with a substratum for long time periods. Doing the analysis using live imaging would be more suitable to assess how often cells interact with their environment. In Movie 4 it appears as if FBCs slide along each other without much interaction - this could be explored more, also showing the interaction of FBCs with other cells in the haemolymph and with the epidermis.

We apologise for the relatively low sample size in Figure 1 G, H. We have now increased the n number of our quantifications significantly and adjusted this in Figure 1F, G, H (dorsal thorax: 424 cell outlines from 6 pupae, ventral thorax: 265 cell outlines from 8 pupae). We believe that these data give a relatively accurate reflection of the cell-cell contacts. Importantly, it highlights that most of the contacts are between neighbouring FBCs. We appreciate the suggestion to additionally look at cell- cell contacts by live imaging. However, this approach comes with its own limitations, since we can't image the cell fully in 3D and would miss contacts on the bottom half. Moreover, it is not possible to label all the different cell types separately.

We agree with the reviewer that cell-cell contacts might affect FBC migration and have already clearly pointed this out in the text. However, in our opinion we do not believe that these contacts are essential for force transmission during FBC migration due to the following reasons: First, it is hard to imagine how traction can be generated efficiently by one swimming FBC pulling on another swimming FBC rather than pulling on a stable substrate. Second, neighbouring cells clearly move independently in different directions. Third, FBCs don't adhere to each other to move as small groups in the same direction. Fourth, having looked at hundreds of WT movies, we have not seen any obvious signs of FBC-FBC contact events being used for traction (e.g. cells speeding up upon contact or signs of physical change/deformation at the contact site). All in all, we currently favour the model that cells mainly use a different mechanism for force transmission and do not use each other to push/pull on each other. Whether and how cell-cell contacts might still play a role will require a more thorough investigation in the future.

2. I am not sure whether 'actin wave' is the best description for what the FBCs' actin network does. Indeed, I find it a little confusing. The authors show that the FBCs contract at their current back (Rho1, Rok and Sqh data). In combination with actin recruitment, this contraction could lead to a movement of part of the cells' actin network by convection (indeed, in Movie 5 one can see moving actin bundles). Alternatively, a contractile wave could move through the cell. Then, the most important aspect of the FBCs behaviour would be an actomyosin contraction rather than an actin wave.

I think that a more careful analysis of what the FBCs are doing would help to better understand the behaviour of their cytoskeleton:
- From the figure panels (Fig. 2) and the movies presented, it does not become sufficiently clear what a wave and what a pulse is. This could be better explained and quantified.

We apologise for this not becoming clear. We describe in lines 192-195:

'Most times, these actin waves travelled a certain distance over the surface of the cell (86% travelling waves, 14% local pulses, n=342; for simplicity, travelling waves and pulses hereafter jointly referred to as 'actin waves')'.

© 2025. Published by The Company of Biologists under the terms of the Creative Commons Attribution License (https://creativecommons.org/licenses/by/4.0/).

To make it clearer, we have now added an example of a wave and a pulse in supplementary figure 3 and new movie 5 and refer to it in line195.

Since many of the pulses are seen on the side of the cell, we suspect that many of the pulses are in fact also traveling waves that came into the field of view from the other side of the FBC that we can't image as they peaked.

- Line 193 - 'This resulted in cell rear retraction and the fast propulsion of the cell body in the opposite direction' - this statement is not sufficiently supported by the data. The correlation of contractility and speed/direction of migration need to be analysed.

We apologise for this not having become clear enough. Our data show that all cells form repetitive actin waves (when imaged over 1h) and 90% of these actin waves form near the cell rear. In other words, the location in the cell where the actin wave peaks is on the opposite side of the cell compared to where the cell moves within the next 2min (as determined by the vector of centroid displacement from this time point and the time point next 2min later). This is what we mean by 'this resulted in rear retraction and propulsion of the cell body in the opposite direction' which tends to be at a high speed, hence 'fast'. Our current data do not provide any direct evidence that waves directly cause increases in speed. We have tried correlating waves with speed increases, but this has proven tricky. Our quantifications have shown that it is not as simple as that, potentially due to other factors such as cells bumping into obstacles. However, despite this, we still believe that our data presented in this manuscript (localisation of actin and myosin at the rear, requirement of actin regulators and myosin for migration and cell deformations) strongly support the idea that actomyosin contraction happens at the rear of the cell resulting in cell movement in the opposite direction.

We have now toned our wording down here to make a more accurate statement by changing ''This resulted in cell rear retraction and the fast propulsion of the cell body in the opposite direction' *to* ''This resulted in cell locomotion in the opposite direction.' *in lines 197-198.*

Moreover, since we have realised that it might have not come across clearly enough in the text and in Figure 2B that actin waves consistently peak at the rear followed by movement in the opposite direction, we have now added a schematic to Figure 2 B to show what we measured (angles of actin waves in relation to cell movement).

- Movie 5 - The phenomenology of the contractile event needs to be described in more detail and across more cells. Due to the variability of what FBCs do, presenting a few examples is not sufficient.

It is unfortunately not straightforward to correlate contractility directly with speed or direction during migration using super resolution microscopy due to the already mentioned limitations (only few cells can be images and only over shorter time periods).We have managed to obtain super-resolution movies of the actin network in 15 WT cells so far. In these, we tend to see that the cell areas where actin bundles concentrate to form a denser actin mesh tend to retract the adjacent cell surface within the next few minutes (as seen for most actin waves in the second super-resolution movie in movie 5). This behaviour is difficult to quantify though due to the complex behaviour and the movement of cells in 3D. In the future it might be possible to image bigger fields of view at this super-resolution over longer time periods and to address some of the questions the reviewer has. We apologise for not providing any further data regarding this.

- Line 333 - 'the effects that actin waves had on cell shape appeared to be very complex and highly variable.' - It would be good to give the numbers for the various described phenotypes.

We agree that it would be important to have a more comprehensive understanding of the types of cell deformations that contractile actin waves can induce. However, this is a very challenging task since the behaviour is very complex and variable and would require studying very large numbers of

© 2025. Published by The Company of Biologists under the terms of the Creative Commons Attribution License (https://creativecommons.org/licenses/by/4.0/).

cells using super resolution microscopy. This is unfortunately currently not possible due to the limitations of super resolution microscopy we already mentioned.

- At the beginning of Movie 5, it looks like a contractile cortical network is contracting and thereby sliding along the cell, squeezing it. This resembles the polarisation of the early C. elegans embryo (Munro et al., 2004). This appears to be different compared to the more local events that the authors describe. How often is this behaviour observed? Also, in Line 432 the authors say - 'First, the cortical actin network in FBCs rarely ever covers the whole cell surface, not even before wave formation'. - this is slightly confusing as the beginning of Movie 5 appears to show exactly this, suggesting that this is a more common occurrence.

As we have just explained above, due to our limitations, we have only been able to image actin by super-resolution in 15 WT cells over a 3-10 minutes time period. We saw this type of behaviour of the actin mesh sliding along the whole surface as shown in movie 5 part 1 in two of these. However, since in both of these movies the cells were imaged when the actin movement had already started, it is hard to know whether at the start there might have been bundles covering the whole cortex.

As mentioned in response to reviewer 1, since we cannot be sure whether areas that seem devoid of actin where we don't see visible bundles indeed are devoid of cortical actin, we have now toned down the wording here too, from the original version: 'First, the cortical actin network in FBCs rarely ever covers the whole cell surface, not even before wave formation'. *To the new version* 'First, the cortical network of actin bundles in FBCs rarely ever covers the whole cell surface, not even before wave formation.' *in lines 450-452.*

3. In the discussion, the authors state that 'Waves can either lead to cell elongation or to rounding' (Line 452). Only here (and in Fig. 7N), it becomes clear that what appears to be happening is that a first contraction is elongating the cell and that a second contraction is rounding it up again. This behaviour should be visible in the data, when correlating actin activity with cell shape change for individual cells. This analysis should be included. In Fig. 7E, such a trend is visible as two actin peaks correlate with one peak and one trough of the aspect ratio data.

We have tried to look at this by looking at the effects of consecutive waves on cell shape. While we can frequently see rounding following an elongation, it does not generally keep on alternating. The behaviour is quite variable. We often see other types of sequences including elongation-elongation-rounding or elongation-rounding-rounding or even elongation-elongation-rounding -rounding. At this stage all we know is that it's fundamental that they change shape frequently and strongly. Our observed variability in deformation behaviour might be due to other factors playing a role in this complex environment, such as cells hitting obstacles or receiving new signals or there being sub population of cells.

4. It remains unclear how the observed FBC behaviour (cell shape change) leads to cell movement. The authors show that dynamic cell deformations are involved in FBC migration (affecting migration speed), however, they do not uncover the mechanism by which the deformations propel the cells. Also, the authors cite Lim et al. (2013) to point out that 'nonreciprocal cell shape changes can theoretically drive force transmission in amoeboid migration', however, they do not discuss sufficiently how this relates to FBC movement.

Our manuscript provides an initial characterisation and description of the actin wave behaviour and the cell deformations caused by actomyosin contractions which we show are key for the swimming migration. Our manuscript does not uncover the mechanism of force transmission, which is an intriguing question, but which is beyond the scope of this paper. Purcell's work in the 70s showed that symmetric/cyclic deformations of a scallop-type shape cannot result in swimming at low Reynold's numbers. Lim et al 2013, following up on this, suggested that nonreciprocal deformations induced during bleb formation and retraction are compatible with swimming at low Reynold's numbers. We feel that it's important to point this out in our discussion since this theoretical model is of interest to the readers. Our findings clearly show that FBCs do not undergo symmetrical cell

© 2025. Published by The Company of Biologists under the terms of the Creative Commons Attribution License (https://creativecommons.org/licenses/by/4.0/).

shape changes during their swimming migration, meaning a deformation being the exact reversal of the previous deformation. Cells rather use more complex and more varied cell deformations including cell elongation and rounding (to different extents) which we would argue are hence nonreciprocal. We currently do not understand the cell deformations that FBCs undergo in enough detail, nor do we know the properties of the hemolymph including its viscosity, to propose a more exact model of what is happening during FBC migration and are planning to address this in collaboration with mathematical modelers in the future.

- Line 330 - 'we next wanted to investigate how these waves drive cell propulsion by looking at the effects that waves have on cell shapes' - This sentence implies that waves are driving cell propulsion, but this has not been formally shown.

Thanks for this comment. We believe that our findings that a wide range of manipulations which leads to a strong reduction in actin waves also leads to a strong reduction in speed, implies that actin waves drive migration and hence cell propulsion. However, to be more cautious in our phrasing of this, we have decided to change this sentence to : 'we next wanted to investigate how these waves drive cell <u>locomotion</u> by looking at the effects that waves have on cell shapes' *in line 340-341.*

- Line 460 - 'Some might compress the rear of a round cell resulting in hydrostatic pressure driven cell elongation and cell propulsion.' - this sentence causally connects cell elongation and cell propulsion, however, this connection is not sufficiently made.

We acknowledge the reviewer's concern and therefore have changed the text to make clear that this is a speculative hypothesis, that we intend on following up in future work. We have changed this to:
'Some might compress the rear of a round cell <u>which we speculate may</u> result in hydrostatic pressure-driven cell elongation and cell propulsion.' *in lines 478-479.*

- It is possible that contraction at the rear drives membrane flow which in turn is propelling the cell.
This could be discussed in more detail. Do the authors have any evidence that excludes or shows that membrane flow occurs?

While this is an interesting hypothesis, we currently have no data in support of this.

Other comments:

Line 324 - 'In agreement with this, we found that RNAi against Wasp as well as against Arp2 or Arp3 led to a significant decrease in FBC migration speed (Suppl.Figure 3). This suggests that Wasp and the Arp2/3 complex are involved in FBC migration and might be the effectors of Cdc42.' - This is not sufficient evidence to suggest that Wasp and Arp2/3 may be the effectors of Cdc42.

We agree. We have now removed from the text the speculation that Wasp and Arp2/3 may be effectors of Cdc42: Lines 336-337: 'This suggests that Wasp and the Arp2/3 complex are involved in FBC migration.'

Also, it would be good to also look at the LifeActin-GFP phenotype in these experiments. How is the actin cytoskeleton affected?

Thanks for this suggestion. See above response to point 6 from reviewer 2. We have quantified actin wave numbers for Wasp and Arp2 RNAi which are significantly reduced and have added these data to Suppl Fig 5J and K.

© 2025. Published by The Company of Biologists under the terms of the Creative Commons Attribution License (https://creativecommons.org/licenses/by/4.0/).

Overall, the role of Rac1 and Cdc42 in the process remains largely unclear.

Line 182 - 'Using LifeAct-GFP to study actin dynamics in swimming FBCs, we found that periodic cortical actin waves were present in all FBCs in the pupal head' - How many cells were assessed?

Thanks for this comment. 194 cells from 12 different pupae imaged over 60 minutes were assessed. We have now added this number in the text in line 188.

The third results paragraph states that 'Random-walk FBC migration is driven by periodic cortical actin waves at the cell rear'. This is in contradiction with the statement in the discussion that 'swimming FBCs have no apparent front rear-polarity' (Line 449). This needs to be made clearer - do the authors mean that a contraction is establishing a new rear?

See above response to minor point 5 from reviewer 1

Minor comments
The first introduction paragraph could have a reference relating more specifically to amoeboid migration.

We have now added a reference (Paluch et al) in line 27.

The following sentence could have references that more specifically makes the point stated (maybe a suitable review on amoeboid migration) - line 45 'Since amoeboid migration does not feature lamellipodia or filopodia, it is thought to be mostly independent of Rac1, Cdc42 or Arp2/3-driven branched actin formation, which mediate the generation of these front protrusions in mesenchymal migration (Ridley, 2001, Ridley, 2015)'.

We apologise for not having added an appropriate reference here in the original version. We have now added a new reference (Garcia-Arcos et al) to line 47.

Line 91 - 'completely' is not necessary.

We have now removed the word "completely" here.

Line 540 - what is the resolution of the super-resolution used?
As mentioned above in response to major point 3 from reviewer 1:
Calculating the point spread function from fluorescent microspheres we found the optimal resolution on this NSPARC microscope to be 110nm. The smallest actin structures that we observed in our 'super-resolution' images and movies of FBCs were 180nm. We have now added more information regarding the parameters used for super-resolution in the methods (lines 560-571).

Second decision letter

MS ID#: jcs.263787R1

MS TITLE: Cell deformations generated by stochastic actomyosin waves drive in vivo random-walk swimming migration

AUTHORS: Anna Franz; Cyril Andrieu; Bren Hunyi Lee
Article Type: Research Article

I am happy to tell you that your manuscript has been accepted for publication in Journal of Cell Science, pending standard publication integrity checks.

© 2025. Published by The Company of Biologists under the terms of the Creative Commons Attribution License (https://creativecommons.org/licenses/by/4.0/).
